# Evolutionary dynamics in the two-locus two-allele model with weak selection

**DOI:** 10.1007/s00285-017-1140-7

**Published:** 2017-05-25

**Authors:** Martin Pontz, Josef Hofbauer, Reinhard Bürger

**Affiliations:** 1 Institut für Mathematik, Universität Wien, Oskar-Morgenstern-Platz 1, 1090 Wien, Austria; 2 Vienna Graduate School of Population Genetics, Vienna, Austria

**Keywords:** Selection, Recombination, Epistasis, Linkage disequilibrium, Equilibrium structure, Phase portrait, 92D15, 37C25

## Abstract

Two-locus two-allele models are among the most studied models in population genetics. The reason is that they are the simplest models to explore the role of epistasis for a variety of important evolutionary problems, including the maintenance of polymorphism and the evolution of genetic incompatibilities. Many specific types of models have been explored. However, due to the mathematical complexity arising from the fact that epistasis generates linkage disequilibrium, few general insights have emerged. Here, we study a simpler problem by assuming that linkage disequilibrium can be ignored. This is a valid approximation if selection is sufficiently weak relative to recombination. The goal of our paper is to characterize all possible equilibrium structures, or more precisely and general, all robust phase portraits or evolutionary flows arising from this weak-selection dynamics. For general fitness matrices, we have not fully accomplished this goal, because some cases remain undecided. However, for many specific classes of fitness schemes, including additive fitnesses, purely additive-by-additive epistasis, haploid selection, multilinear epistasis, marginal overdominance or underdominance, and the symmetric viability model, we obtain complete characterizations of the possible equilibrium structures and, in several cases, even of all possible phase portraits. A central point in our analysis is the inference of the number and stability of fully polymorphic equilibria from the boundary flow, i.e., from the dynamics at the four marginal single-locus subsystems. The key mathematical ingredient for this is index theory. The specific form of epistasis has both a big influence on the possible boundary flows as well as on the internal equilibrium structure admitted by a given boundary flow.

**Electronic supplementary material:**

The online version of this article (doi:10.1007/s00285-017-1140-7) contains supplementary material, which is available to authorized users.

## Introduction

One of the central goals of the pioneers of population genetics was to demonstrate that the inheritance and evolution of continuously varying traits could be explained on the basis of Mendelian genetics (Fisher 1918, 1930). Haldane (1931) and Wright (1935) were apparently the first who formulated explicit dynamical models for the evolution of gene frequencies if selection acts on more than one locus. Under various assumptions about dominance, Haldane considered two loci at each of which a wild type and a deleterious variant segregate. Motivated by empirical examples, he assumed that if both variants occur in the same genotype, they have a selective advantage. Wright investigated a model in which finitely many loci contribute to a quantitative trait that is under quadratic selection toward an intermediate optimum. Both Haldane and Wright assumed that gene frequencies at the loci are probabilistically independent, i.e., they are in linkage equilibrium, and derived the stable equilibrium states as well as other properties of their models.

In an investigation designed to show that selection can lead to tighter linkage, Kimura (1956) derived and studied a full two-locus two-allele model, i.e., one that takes into account linkage disequilibrium. The general (deterministic) two-locus two-allele model for the evolutionary dynamics under selection and recombination was derived and investigated by Lewontin and Kojima (1960). They deduced both general properties as well as properties of special cases, such as additive gene action. Among others, they showed that strong epistasis together with linkage disequilibrium can lead to significantly different outcomes than would occur for independent loci.

Extensive analyses of a special class of fitness patterns, the so-called symmetric viability model (which originated from Wright’s and from Kimura’s work), were performed by Bodmer and Felsenstein (1967) and Feldman and Karlin (1970). The latter authors derived all fifteen possible equilibria and determined their stability for several special cases. Later, Feldman and Liberman (1979) showed that as many as four boundary equilibria and two polymorphic equilibria can be simultaneously stable, and Hastings (1985) demonstrated that up to four stable internal equilibria may coexist. The complexity of this model is also underlined by the finding of Ewens (1968) that there is a gap in the range of recombination rates for which a pair of internal equilibria is stable. A comprehensive review of this model and its extension to multiple loci can be found in Christiansen (1999).

Other important special classes of fitness patterns are the additive and the multiplicative model, in which fitnesses of multilocus genotypes are obtained by adding or multiplying the fitnesses of the constituent single-locus genotypes. In the former, additive epistasis is absent, in the latter, multiplicative epistasis is absent. For additive fitnesses, mean fitness is a Lyapunov function (Ewens 1969), all equilibria are in linkage equilibrium, and, generically, every trajectory converges to an equilibrium point (Karlin and Liberman 1978, 1990; Nagylaki et al. 1999). The multiplicative model is much more complicated, although the linkage-equilibrium manifold is invariant. In this model and away from the linkage-equilibrium manifold, mean fitness may decrease and, for intermediate recombination rates, asymptotically stable equilibria may exist that are in linkage disequilibrium (e.g., Moran 1964, 1968; Bodmer and Felsenstein 1967; Nagylaki 1977; Karlin and Feldman 1978; Hastings 1981a). A detailed review of the theory of two-locus and multilocus models is given in Bürger (2000, Chap. 2).

With general fitnesses, the dynamics in the two-locus two-allele model can be complex. The existence of stable limit cycles has been demonstrated both for the continuous-time model (Akin 1979, 1982) and the discrete-time model (Hastings 1981b; Hofbauer and Iooss 1984). Such complex behavior cannot occur if loci are assumed to be independent, i.e., if linkage equilibrium is imposed. Then the dynamics is gradient-like and mean fitness is a global Lyapunov function (Nagylaki 1989).

However, even the case of two independent, diallelic loci has never been analyzed systematically. Although a Lyapunov function exists, the equilibrium structure can still be quite complicated. For instance, Moran (1963) showed that, apart from degenerate cases, the maximum number of internal (polymorphic) equilibria is five, and up to three can be asymptotically stable.

In this study we perform a systematic analysis of the two-locus two-allele model with constant fitnesses under the assumption of linkage equilibrium. The goal is to determine and classify all possible equilibrium structures and phase portraits (Sects. 3, 4). We assume continuous time for reasons outlined below. We have not fully accomplished our goal, however, we identified all 42 possible (equivalence classes of) boundary flows and 190 potentially possible extended boundary flows, i.e., flows at or close to the boundary. Of these 190 extended boundary flows, the existence of 185 could be proved; the other cases remain undecided. In Section S1 of the Supplementary Information (SI), we present corresponding phase portraits. A large number of extended boundary flows admits not only several non-equivalent phase portraits, but also more than one equilibrium structure, as characterized by the number and stability of boundary and internal equilibria.

We use this general analysis to obtain a detailed classification of equilibrium structures and phase portraits for a number of important special cases that have received considerable attention in the literature. These include the case of marginal overdominance or underdominance (Sect. 5); linear isoclines, which turn out to exist if and only if fitnesses among loci are additive or the only epistatic interactions are additive-by-additive (Sect. 6); multilinear epistasis (Sect. 7); equivalent loci (Sect. 8); and the symmetric viability model (Sect. 9). These results provide considerable insight into the interplay of dominance and epistasis in maintaining genetic polymorphism.

The analysis of this simplified model has immediate implications for the full two-locus two-allele model. This is a consequence of a general theorem by Nagylaki et al. (1999), which applies to multilocus systems. These authors proved under weak technical assumptions that if selection is much weaker than recombination, then after an evolutionarily short period, in which linkage disequilibrium decays to close to zero, the dynamics of the full model (either in discrete or in continuous time) is governed by this weak-selection limit. The model investigated in this paper is the weak-selection limit of the two-locus two-allele model with selection and recombination.

## Model

We start with the standard two-locus two-allele model with viability selection and discrete time. Thus, we assume a randomly mating, diploid population with discrete and non-overlapping generations in which viability selection acts on two diallelic, recombining loci. Therefore, gametes are in Hardy–Weinberg proportions. Mutation, random drift, and other evolutionary forces are absent.

Let $A_1$ and $A_2$ be the alleles at locus *A*, and $B_1$ and $B_2$ those at locus *B*. Let the frequencies of the four gametes $A_1B_1$, $A_1B_2$, $A_2B_1$, and $A_2B_2$ be denoted by $x_1$, $x_2$, $x_3$, and $x_4$, respectively, where $\sum _{i=1}^4{x_i}=1$, and let $w_{ij}>0$ denote the (constant) viability of an individual with genotype *ij*. In addition to positing absence of sex effects, i.e., $w_{ij}=w_{ji}$, we posit absence of position effects, i.e., $w_{14}=w_{23}$. The recombination probability is denoted by *r*. The frequencies of alleles $A_1$ and $B_1$ are denoted by $p=x_1+x_2$ and $q=x_1+x_3$, respectively. Letting $D=x_1x_4-x_2x_3$ be the classical measure of linkage disequilibrium, we obtain

2.1 $$\begin{aligned} \begin{aligned} x_1&=pq+D, \\ x_2&=p(1-q)-D, \\ x_3&=(1-p)q-D, \\ x_4&=(1-p)(1-q)+D. \end{aligned} \end{aligned}$$

Under the above symmetry assumptions, the fitnesses of genotypes are completely specified by the following matrix:2.2Then evolution of gamete frequencies is given by (Lewontin and Kojima 1960)

2.3 $$\begin{aligned} x'_i=\frac{x_iw_i -\eta _i w_{14}rD}{\bar{\omega }}, \quad i=1,2,3,4, \end{aligned}$$

where $\eta _1=\eta _4=-\eta _2=-\eta _3=1$, $w_i=\sum _{j=1}^4{w_{ij}x_j}$ is the marginal fitness of gamete *i*, and $\bar{\omega }=\sum _{j=1}^4{w_jx_j}$ is the mean fitness of the population. This is a dynamical system on the simplex $S_4$ which has received much attention in the literature but is well understood only in special cases (see Sect. 1). For a review consult Chapter 2 in Bürger (2000).

If the assumption of linkage equilibrium, i.e., $D=0$, is imposed, the dynamics (2.3) simplifies to the following system of difference equations defined on the unit square $[0,1]\times [0,1]$,

2.4a $$\begin{aligned} \Delta p&= p(1-p) \frac{1}{2{\bar{w}}}\frac{\partial {\bar{w}}}{\partial p}, \end{aligned}$$

2.4b $$\begin{aligned} \Delta q&= q(1-q) \frac{1}{2{\bar{w}}}\frac{\partial {\bar{w}}}{\partial q}, \end{aligned}$$

where by (2.1) mean fitness ${\bar{w}}=\bar{\omega }(D=0)$ is only a function of *p* and *q*; cf. Haldane (1931) and Wright (1935, 1942). As shown more generally for multiple multiallelic loci by Nagylaki (1989), ${\bar{w}}$ is monotone increasing along trajectories of (2.4) and constant only at equilibria. Therefore, if all equilibria are isolated points, every trajectory converges to an equilibrium.

In general, the manifold $D=0$ is not invariant under (2.3). However, assuming weak selection, i.e., setting $w_{ij}=1+ sm_{ij}$, rescaling time according to $t=\lfloor \tau /s\rfloor$, and letting $s\downarrow 0$, the so called *weak-selection limit* of (2.3) is obtained:

2.5a $$\begin{aligned} \dot{p}&= p(1-p) \frac{1}{2}\frac{\partial {\bar{m}}}{\partial p}, \end{aligned}$$

2.5b $$\begin{aligned} \dot{q}&= q(1-q) \frac{1}{2}\frac{\partial {\bar{m}}}{\partial q}. \end{aligned}$$

Here,

2.6 $$\begin{aligned} {\bar{m}}= m_1pq + m_2p(1-q) + m_3(1-p)q + m_4(1-p)(1-q) \end{aligned}$$

is the mean (Malthusian) fitness of the population and

2.7 $$\begin{aligned} m_i = m_{i1}pq + m_{i2}p(1-q) + m_{i3}(1-p)q + m_{i4}(1-p)(1-q) \end{aligned}$$

the marginal (Malthusian) fitness of gamete *i*. We note that the dynamics (2.5) remains unchanged if the same constant is added to every $m_{ij}$. If every $m_{ij}$ is multiplied by the same positive constant, only a change in time scale results. Therefore, in (2.5) we could substitute $w_{ij}$ for $m_{ij}$ without changing the phase portrait. In particular, two of the nine parameters in the fitness scheme (2.2) could be set to fixed, different values.

Nagylaki et al. (1999) proved (for multiple multiallelic loci) that if $r>0$ is given, selection is sufficiently weak, i.e., $s>0$ is sufficiently small, and if all equilibria of (2.5) are hyperbolic, then every trajectory of (2.3) converges to an equilibrium point on an invariant manifold, $\Lambda _s$, which is contained in an *O*(*s*) neighborhood of the linkage-equilibrium manifold $D=0$. The dynamics on $\Lambda _s$ is a small perturbation of the time-*s* map of the weak-selection limit (2.5), which is gradient-like. In particular, it is easy to show directly for (2.5), but also follows from a result by Nagylaki (1989) for (2.4), that $\dot{{\bar{m}}}\ge 0$ for every $(p,q)\in [0,1]^2$ and $\dot{{\bar{m}}}=0$ if and only if (*p*, *q*) is an equilibrium. Therefore, ${\bar{m}}$ is a strict Lyapunov function for (2.5).

Because ${\bar{w}}=1+s{\bar{m}}$, the equilibria of (2.4) and (2.5) are the same, and so are their stability properties (since ${\bar{w}}\ge \min _{i,j}w_{ij}>0$). Therefore, if selection is sufficiently weak and after some (usually short) time has passed (Nagylaki 1993), the dynamics of the full two-locus system (2.3) is closely approximated by the dynamics of the weak-selection limit (2.5). Therefore, each solution of (2.3) converges to an equilibrium point, and this equilibrium point is in an *O*(*s*) neighborhood of an equilibrium of (2.5) (Theorem 3.1 in Nagylaki et al. 1999).

## Equilibria and their stability

**Fig. 1:**
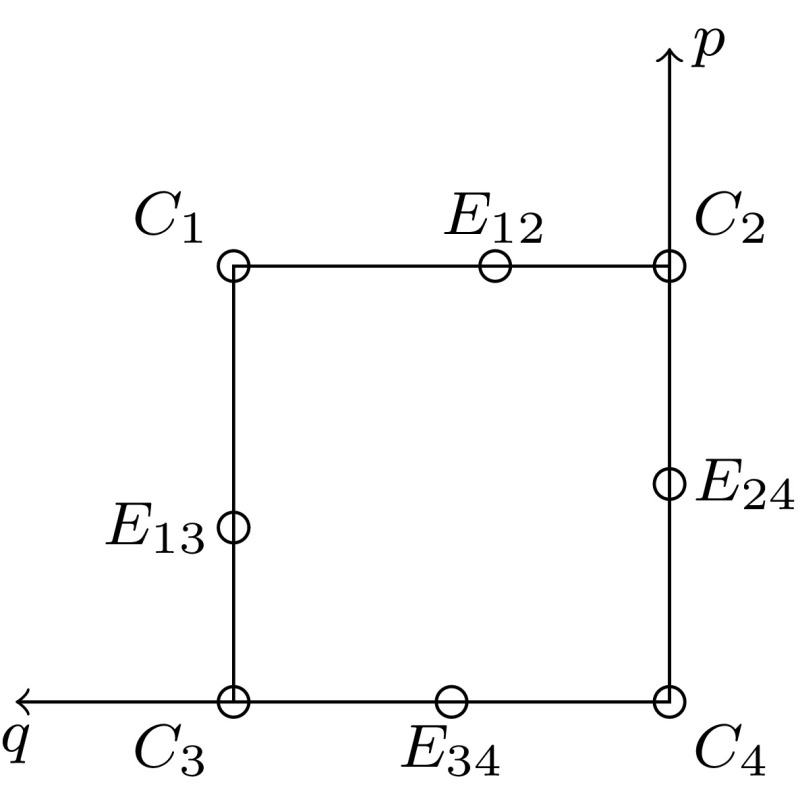
The state space with all possible boundary equilibria. The orientation is such that it corresponds to the fitness matrix in (2.2)

The four monomorphic equilibria $C_i$ of (2.5), corresponding to fixation of gamete *i*, exist always. They represent the corners of the state space $[0,1]^2$ (Fig. 1). The eigenvalues of the Jacobian at $C_i$ are easily calculated and are as follows:3.1An equilibrium is called linearly stable, or a sink, if all eigenvalues (of its Jacobian) have negative real part. If at least one eigenvalue has positive real part, it is linearly unstable. It is called a source if all eigenvalues have positive real part, and it is a saddle if eigenvalues with positive and negative real part occur. Obviously, the corner equilibrium $C_i$ is linearly stable if and only if the fitness of the homozygous genotype *ii* is higher than each of the two ‘neighboring’ single-locus heterozygous genotypes *ij*, where *j* differs from *i* by a single allele.

Next there may exist up to four equilibria at which one locus is polymorphic, so-called single-locus polymorphisms (SLPs). They are located on the edges of the state space. We denote the equilibrium on the edge connecting $C_i$ with $C_j$ by $E_{ij}$ (Fig. 1). The coordinates of these edge equilibria are easily calculated and are given by

3.2a $$\begin{aligned} E_{ij}{:}\ q&=\frac{m_{ij}-m_{jj}}{2m_{ij}-m_{ii}-m_{jj}} \ \text { and } p=0 \text { if } ij=34, \text { and } p=1 \text { if } ij=12; \end{aligned}$$

3.2b $$\begin{aligned} E_{ij}{:}\ p&=\frac{m_{ij}-m_{jj}}{2m_{ij}-m_{ii}-m_{jj}} \ \text { and } q=0 \text { if } ij=24, \text { and } q=1 \text { if } ij=13. \end{aligned}$$

Therefore, the edge equilibrium $E_{ij}$ exists, i.e., is in the interior of the edge, if and only if $(m_{ij}-m_{ii})(m_{ij}-m_{jj})>0$. Obviously, this is just the well-known condition of either overdominance or underdominance applied to a one-locus boundary system. To distinguish this notion of overdominance (underdominance) from that of marginal overdominance (underdominance) introduced below, we call this overdominance (underdominance) on an edge.

The eigenvalues of the edge equilibria, hence their stability, can also be determined quite straightforwardly, because the Jacobian is in triangular form, so that the eigenvalues appear on the diagonal. One eigenvalue, called internal, determines stability of this equilibrium within its edge. For $E_{ij}$, it is given by

3.3 $$\begin{aligned} \mu _{ij} = \frac{(m_{ij}-m_{ii})(m_{ij}-m_{jj})}{m_{ii}+m_{jj}-2m_{ij}}. \end{aligned}$$

Therefore, as is well known, the SLP $E_{ij}$ is linearly stable (unstable) within its edge if and only if there is overdominance (underdominance) on this edge.

The other eigenvalue, called external, determines stability of the edge equilibrium transversal to the boundary. If the external eigenvalue is negative, the equilibrium is called *strictly* *saturated* or *externally stable* (Hofbauer and Sigmund 1998; Karlin 1980). This has the interpretation that the allele missing on this edge cannot invade the population near this boundary equilibrium. The external eigenvalues are of more complicated form than the internal eigenvalues and are presented in “Appendix A.1”. The distinction between external and internal eigenvalues will be essential for our analysis.

Thus, there are at least four boundary equilibria, but there may be up to eight. We ignore the degenerate cases, in which every point on an edge is an equilibrium. This occurs if and only if $m_{ii}=m_{ij}=m_{jj}$, where *i* and *j* are gametes differing by one allele.

Finally, we turn to the internal, or fully polymorphic, equilibria. By (2.5), they are the solutions of the two equations $\partial {\bar{m}}/\partial p=0$ and $\partial {\bar{m}}/\partial q=0$ that satisfy $0<p<1$ and $0<q<1$. Equivalently, these equations can be written as

3.4a $$\begin{aligned} p&=f(q)=\frac{m_{A_1A_2}-m_{A_2A_2}}{2m_{A_1A_2}-m_{A_1A_1}-m_{A_2A_2}}, \end{aligned}$$

3.4b $$\begin{aligned} q&=g(p)=\frac{m_{B_1B_2}-m_{B_2B_2}}{2m_{B_1B_2}-m_{B_1B_1}-m_{B_2B_2}}, \end{aligned}$$

where

3.5a $$\begin{aligned} m_{A_1A_1}&=m_{11}q^2+2m_{12}q(1-q)+m_{22}(1-q)^2, \end{aligned}$$

3.5b $$\begin{aligned} m_{A_1A_2}&=m_{13}q^2+2m_{14}q(1-q)+m_{24}(1-q)^2, \end{aligned}$$

3.5c $$\begin{aligned} m_{A_2A_2}&=m_{33}q^2+2m_{34}q(1-q)+m_{44}(1-q)^2, \end{aligned}$$

and

3.6a $$\begin{aligned} m_{B_1B_1}&=m_{11}p^2+2m_{13}p(1-p)+m_{33}(1-p)^2, \end{aligned}$$

3.6b $$\begin{aligned} m_{B_1B_2}&=m_{12}p^2+2m_{14}p(1-p)+m_{34}(1-p)^2, \end{aligned}$$

3.6c $$\begin{aligned} m_{B_2B_2}&=m_{22}p^2+2m_{24}p(1-p)+m_{44}(1-p)^2, \end{aligned}$$

are the marginal fitnesses of the one-locus genotypes at *A* and *B*, respectively. (In the presence of linkage disequilibrium, the expressions in (3.5) and (3.6) need to be normalized; see Ewens and Thomson 1977.) Thus, the internal equilibria are the intersection points of the isoclines $p=f(q)$ and $q=g(p)$.

The following results were proved by Moran (1963). We will give a slightly different proof of the first statement.

### Theorem 3.1

- If all equilibria are isolated, then (2.5) has at most five internal equilibria.
- Five internal equilibria can be realized, and up to three can be sinks.
- Sinks correspond to local maxima of ${\bar{m}}$.

### Proof

- To determine the intersection points of the isoclines (3.4a) and (3.4b), we have to solve the fixed point equation
  3.7 $$\begin{aligned} p=f(g(p)). \end{aligned}$$
  Because the numerators and denominators of *f*(*q*) and *g*(*p*) are polynomials of degree two or less, numerator and denominator of the rational function *f*(*g*(*p*)) are polynomials of degree four or less. Therefore the intersection points, hence the internal equilibria, are the zeros of a polynomial of degree five or less.
- See Moran (1963) and panel 6 in Fig. S1d of the SI, which shows the phase portrait of Moran’s example. (c) is also shown by Moran (1963). It follows immediately from the fact that ${\bar{m}}$ is a strict Lyapunov function.

To determine the stability of an internal equilibrium, we need the Jacobian.

### Lemma 3.2

The Jacobian at an internal equilibrium (*p*, *q*) is given by

3.8 $$\begin{aligned} J = J(p,q) = \begin{pmatrix} p(1-p)m_A&{}\quad 2p(1-p)\tilde{m}\\ 2q(1-q)\tilde{m}&{}\quad q(1-q)m_B \end{pmatrix}, \end{aligned}$$

where $m_A=m_{A_1A_1}+m_{A_2A_2}-2m_{A_1A_2}$, $m_B=m_{B_1B_1}+m_{B_2B_2}-2m_{B_1B_2}$, and $\tilde{m}=m_1-m_2-m_3+m_4$. The eigenvalues of *J* are real.

### Proof

A simple calculation shows that

3.9 $$\begin{aligned} \frac{1}{2}\frac{\partial {\bar{m}}}{\partial p} = p m_A + m_{A_1A_2}-m_{A_2A_2}. \end{aligned}$$

Therefore, (2.5a) yields

3.10 $$\begin{aligned} \frac{\partial \dot{p}}{\partial p} = (1-2p)\frac{1}{2}\frac{\partial {\bar{m}}}{\partial p} + p(1-p)\frac{1}{2}\frac{\partial ^2{\bar{m}}}{\partial p^2} = p(1-p)m_A, \end{aligned}$$

because $\frac{\partial {\bar{m}}}{\partial p}=0$ at an internal equilibrium and $m_A=\frac{1}{2}\frac{\partial ^2{\bar{m}}}{\partial p^2}$. The other derivatives in *J* are calculated in a similar way.

Finally, it is straightforward to compute the discriminant of *J*, which is the square of the trace minus four times the determinant:

$$\begin{aligned} ({\text {tr}}J)^2 - 4\det J =[p(1-p)m_A-q(1-q)m_B]^2+16pq(1-p)(1-q)\tilde{m}^2 > 0. \end{aligned}$$

The inequality holds because $(p,q)\in (0,1)^2$. Therefore, all eigenvalues are real. $\square$

For the rest of this paper, we impose the assumption 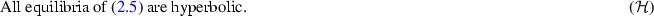


Therefore, eigenvalues at equilibria are negative or positive, but not zero. This assumptions excludes not only curves of equilibria, but also complete dominance or recessivity of an allele.

The stability of an internal equilibrium is most easily determined by employing the planar Routh–Hurwitz criterion. It states that an equilibrium is (i) a saddle point if $\det J<0$, (ii) a sink if $\det J>0$ and ${\text {tr}}J<0$, and (iii) a source if $\det J>0$ and ${\text {tr}}J>0$.

Motivated by Lewontin and Kojima (1960), we say that a locus (e.g., *A*) exhibits marginal, or induced, overdominance at (*p*, *q*) if the inequalities

3.11 $$\begin{aligned} m_{A_1A_2}>m_{A_1A_1}\; \text { and }\; m_{A_1A_2}>m_{A_2A_2} \end{aligned}$$

hold. If both inequality signs are reversed, one obtains marginal, or induced, underdominance. The following result was proved by Kojima (1959). We will give a similar, but more direct, proof.

### Corollary 3.3

An internal equilibrium (*p*, *q*) is linearly stable, i.e., a sink, if and only if both loci exhibit marginal overdominance at (*p*, *q*) and $m_A m_B > 4{{\tilde{m}}}^2$.

### Proof

By the Routh–Hurwitz criterion, the equilibrium (*p*, *q*) is a sink if and only if $p(1-p)m_A+q(1-q)m_B<0$ and

3.12 $$\begin{aligned} \det J(p,q) = pq(1-p)(1-q)(m_Am_B - 4\tilde{m}^2) >0. \end{aligned}$$

Therefore, both $m_A$ and $m_B$ must be negative. Because $0<p<1$ must hold, (3.4) implies that $m_A$, $m_{A_2A_2}-m_{A_1A_2}$, and $m_{A_1A_1}-m_{A_1A_2}$ all have the same sign. This together with an analogous argument for locus *B* proves that marginal overdominance is necessary. The sufficiency condition follows immediately from (3.12). $\square$

### Remark 3.4

(i) As pointed out by Kojima (1959), (3.12) is equivalent to the condition that the geometric mean of the dominance variances of each locus exceeds the additive-by-additive variance of the two-locus system.
(ii) The proof of Corollary 3.3 shows that, at equilibrium, locus *A* displays marginal overdominance if and only if (cf. Hastings 1982)
  3.13 $$\begin{aligned} m_{A_1A_2}^2 > m_{A_1A_1}m_{A_2A_2}. \end{aligned}$$

## Equilibrium structure and flows

Since the internal equilibria are obtained from the solutions of the quintic polynomial equation (3.7), it is generally difficult to determine their number, position, or stability. Therefore, we first study the possible flows on the boundary of the state space. Subsequently, we extend the flows to a neighborhood of the boundary. Using index theory, we will be able to shed light on the equilibrium structure, i.e., the number and stability properties of equilibria (for a precise definition, see Sect. 4.3), and on the possible phase portraits, i.e., the topological structures of the flow (2.5). Finally, we exclude several potentially possible equilibrium structures and generate phase portraits for most of the remaining cases.

To facilitate the characterization of equilibrium structures and phase portraits, we identify flows that are topologically equivalent or obtained by symmetry operations corresponding to a relabeling of alleles at a locus ($A_1$ or $A_2$, $B_1$ or $B_2$) or of the loci (*A* or *B*). More precisely, we call two systems of the form (2.5), or the corresponding fitness schemes *M* and ${\tilde{M}}$ in ${\mathsf {R}}^{3 \times 3}$ (2.2), or the flows, *equivalent* if there exists an edge-preserving homeomorphism *h* of $[0,1]^2$ onto itself that maps orbits of (2.5) generated by *M* onto orbits generated by ${\tilde{M}}$ (preserving the arrow of time), i.e., the phase portraits are topologically equivalent. Here, edge-preserving means that each of the four edges is mapped onto an edge, not necessarily onto itself.

An edge-preserving homeomorphism is a composition of one of the eight symmetry operations of the square with a ‘proper’ or ‘pinned’ homeomorphism of the square, i.e., a homeomorphism that leaves the four corners fixed and maps each edge onto itself. The symmetry group of the square, the dihedral group $D_4$, consists of four reflections and four rotations (including the identity). Therefore, each equivalence class is invariant under rotations by multiples of $90^\circ$, reflections about the diagonal or antidiagonal, and reflections about the middle vertical or horizontal axis of the matrix (2.2), or of the unit square (Fig. 1).

Finally, we call a fitness scheme *M* [or its induced flow (2.5), or its phase portrait] *robust* if it has a neighborhood of equivalents in ${\mathsf {R}}^{3 \times 3}$. This is essentially the concept of structural stability, adapted to the selection Eq. (2.5). For the single-locus two-allele model, i.e., on every edge, there are three robust equivalence classes: they correspond to the classical selection patterns of overdominance, underdominance, or intermediate dominance (directional selection).

The goal of our paper is to find all robust (equivalence classes of) phase portraits arising from (2.5). A necessary condition for robustness is that all equilibria are hyperbolic, i.e., condition (${\mathcal {H}}$) holds. A classical characterization of structural stability in two-dimensional systems due to Andronov and Pontryagin (1937) implies:

*If (* 2.5 *) satisfies (* ${\mathcal {H}}$ *) and there is no saddle connection in the interior of* $[0,1]^2$, *then this system is robust.*

A saddle connection is an orbit which has one saddle as its $\alpha$-limit and an other as its $\omega$-limit. Most phase portraits shown in Section S1 have no saddle connection. Exceptions occur for the symmetry classes ${\mathsf {s}}$, ${\mathsf {b}}$, and ${\mathsf {e}}$ (defined below), where the phase portraits were generated by matrices satisfying the corresponding symmetry condition. In all these cases, however, breaking the symmetry yields phase portraits that are members of the same class. Thus, they are robust in this sense. We now explain how to obtain all phase portraits. This requires three steps.

### Flows on the boundary

**Fig. 2:**
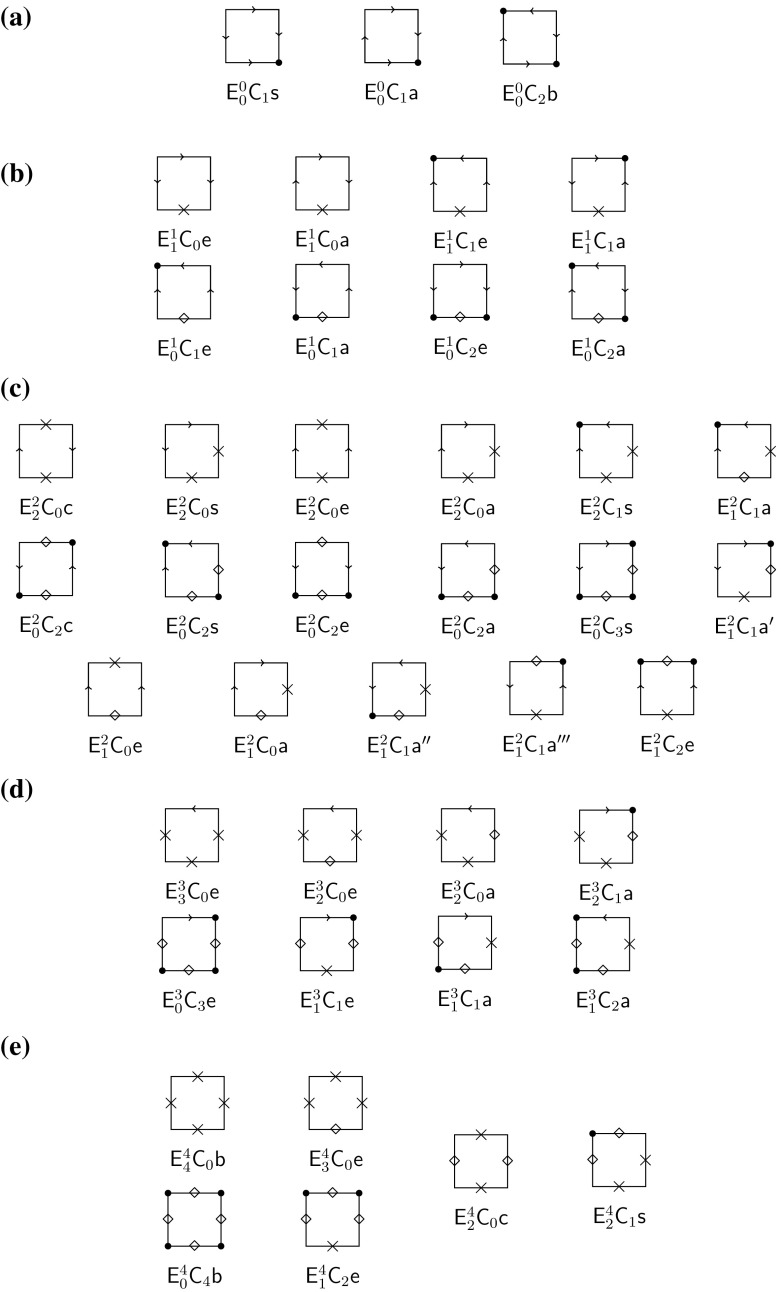
The 42 possible boundary flows, or boundary-flow equivalence classes, for (2.5). The 16 flow-reversal pairs are arranged *vertically*. For instance, ${\mathsf {E}}^2_0{\mathsf {C}}_2{\mathsf {c}}$ is the reversed boundary flow of ${\mathsf {E}}^2_2{\mathsf {C}}_0{\mathsf {c}}$, whereas ${\mathsf {E}}^2_1{\mathsf {C}}_0{\mathsf {e}}$ is self inverse. Internal stability is indicated by a *solid dot* (at a *corner*) or a cross (at an edge equilibrium). A *rhombus* indicates an internally unstable edge equilibrium

As shown in Sect. 3, on each edge there is either no or one equilibrium, and each edge equilibrium can be internally stable or unstable. This gives $4^4 = 256$ different types of (nondegenerate) flows on the boundary. This type can be easily determined from the selection scheme (2.2), by observing the order relations in each boundary row and boundary column, i.e., overdominance, underdominance, or intermediate dominance. Applying the symmetry operations, their number drops to 42 different boundary flows or, more precisely, boundary-flow equivalence classes. They are displayed in Fig. 2.

We recall that a matrix is called centrosymmetric if it is invariant under reflections about the central entry, and bisymmetric if it is symmetric and centrosymmetric. A centrosymmetric fitness matrix (2.2) gives rise to the well-studied symmetric viability model (Sect. 9). A symmetric fitness matrix (2.2) represents a model in which the loci *A* and *B* are equivalent (Sect. 8).

The following code was used to label boundary flows: ${\mathsf {E}}^n_m{\mathsf {C}}_k{{\mathsf {x}}}$ represents a flow of symmetry class ${{\mathsf {x}}}\in \{{\mathsf {b}},{\mathsf {c}},{\mathsf {s}},{\mathsf {e}},{\mathsf {a}}\}$ with *n* edge equilibria, *m* of them internally stable, and *k* linearly stable corner equilibria. Here, ${\mathsf {b}}$, ${\mathsf {c}}$, or ${\mathsf {s}}$ means that at least one flow in this class is generated by a bisymmetric, centrosymmetric, or symmetric matrix, respectively. This does not imply that all matrices generating a flow in such a class have the respective symmetry property. The letter ${\mathsf {e}}$ refers to a flow for which the flows at (at least) one pair of opposite edges belong to the same single-locus class and have the same direction if there is intermediate dominance. This is the case if the entries in the two boundary columns (or rows) of the fitness scheme have the same order relation. The letter ${\mathsf {a}}$ indicates asymmetry, i.e., the boundary flow cannot be generated by a matrix with one of the properties ${\mathsf {b}}$, ${\mathsf {c}}$, ${\mathsf {s}}$, or ${\mathsf {e}}$. In one case, ${\mathsf {E}}^2_1{\mathsf {C}}_1{\mathsf {a}}$, the above code is not sufficient to identify the equivalence class; therefore, we used ${\mathsf {E}}^2_1{\mathsf {C}}_1{\mathsf {a}}'$, ${\mathsf {E}}^2_1{\mathsf {C}}_1{\mathsf {a}}''$, ${\mathsf {E}}^2_1{\mathsf {C}}_1{\mathsf {a}}'''$.

By reversing all arrows in a flow, the reversed flow is obtained. Equivalently, the signs of all entries in the fitness matrix are reversed. It turns out that 10 of the 42 cases are invariant under flow reversal. In Fig. 2, the other 32 cases are placed such that pairs obtained by reversal are arranged vertically.

### Extended boundary flows

**Fig. 3:**
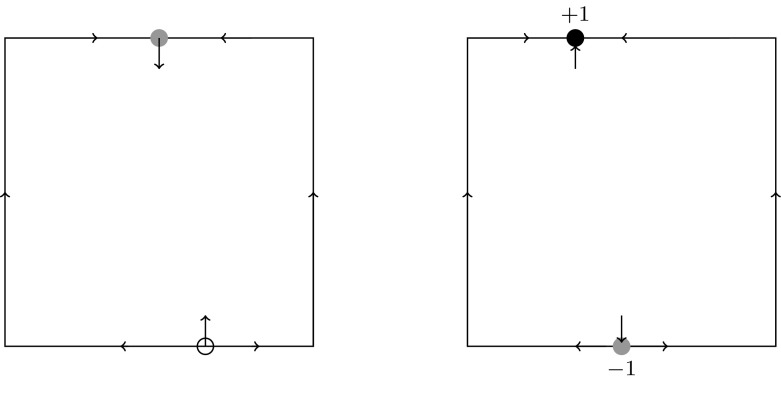
The boundary-flow class ${\mathsf {E}}^2_1{\mathsf {C}}_0{\mathsf {e}}$ with positive (*left*) and negative (*right*) external eigenvalues at the edge equilibria. In the *left panel*, the edge equilibria are not saturated. In the *right panel*, $E_{12}$ is asymptotically stable and saturated with index $+1$, and $E_{34}$ is a saturated saddle with index $-1$. Therefore, $\delta =0$ in both panels. If the flow in the *left panel* is reversed, i.e., all *arrows* are reversed, the flow in the *right panel* is obtained after a rotation by $180^\circ$. Extended boundary flows, where the flow reversal has the same boundary flow and $\delta$ but a different external stability, can occur only for boundary-flow classes that are invariant under flow reversal

Now we consider flows not only on the boundary, but in a sufficiently small neighborhood of the boundary. Here, external stability of the edge equilibria plays a central role (Fig. 3). We need some preparation and recall from below Eq. (3.3) when an equilibrium in our two-dimensional model is strictly saturated. Because we impose the hyperbolicity assumption (${\mathcal {H}}$) throughout, we simply use the term saturated equilibrium.

Therefore, a corner equilibrium is *saturated* if and only if both eigenvalues are negative. An edge equilibrium is saturated if and only if the external eigenvalue is negative. An internal equilibrium is, by definition, always saturated. For a hyperbolic equilibrium $\hat{x}$, the index is defined by

4.1 $$\begin{aligned} {\text {ind}}(\hat{x}) = {\text {sgn}}(\det (-J_{\hat{x}})) = (-1)^k, \end{aligned}$$

where *k* denotes the number of positive eigenvalues of the Jacobian $J_{\hat{x}}$ at $\hat{x}$. It follows, that in a planar system an equilibrium with index $-1$ is a saddle point; sources and sinks have index $+1$. The sum of the indices of all saturated boundary equilibria is called the *boundary index sum* and is denoted by $\delta$. If there are no saturated boundary equilibria, then $\delta =0$.

In general, $\delta$ is not uniquely determined by the boundary flow because external eigenvalues of edge equilibria may be positive or negative for a given boundary flow (cf. Fig. 3). However, $\delta$ is uniquely determined by the *extended boundary flow*, which we use as a short hand for the equivalence class (in the above sense) of flows with a given boundary flow (class) together with the signs of the external eigenvalues of all edge equilibria. Different extended boundary flows of the same boundary-flow class may have the same $\delta$.

#### Lemma 4.1

For a boundary flow of type ${\mathsf {E}}^n_m{\mathsf {C}}_k$, the boundary index sum $\delta$ can assume at most the (integer) values satisfying

4.2 $$\begin{aligned} k+m-n \le \delta \le k+m. \end{aligned}$$

Every boundary flow in Fig. 2 satisfies $k+m-n\ge -2$ and $k+m\le 4$.

#### Proof

We have to sum the indices of the saturated equilibria. Obviously, there are *k* stable, hence saturated, corner equilibria and at most *m* saturated, internally stable edge equilibria. This yields the upper bound $k+m$. Because the number of saturated, internally unstable edge equilibria is at most $n-m$, the lower bound $k-(n-m)$ results.

The second statement follows easily by checking Fig. 2. $\square$

By systematic construction of the extended boundary flows for each of the 42 boundary flows, we obtain 200 potential extended boundary flows (see Table S1). It is important to recall that for given $\delta$ more than one extended boundary flow may exist. In the following we show that not all of these 200 extended boundary flows can be realized for (2.5).

#### Theorem 4.2

Assume (2.5) and ($${\mathcal {H}}$$).

- The boundary index sum $\delta =-2$ cannot occur.
- The boundary index sum $\delta =-1$ cannot occur for any of the boundary flows ${\mathsf {E}}^4_2{\mathsf {C}}_0{\mathsf {c}}$, ${\mathsf {E}}^3_2{\mathsf {C}}_0{\mathsf {e}}$, ${\mathsf {E}}^3_1{\mathsf {C}}_1{\mathsf {e}}$, ${\mathsf {E}}^2_1{\mathsf {C}}_0{\mathsf {e}}$, or ${\mathsf {E}}^2_1{\mathsf {C}}_0{\mathsf {a}}$.
- The boundary index sum $\delta =0$ can neither occur for ${\mathsf {E}}^3_2{\mathsf {C}}_0{\mathsf {e}}$ nor ${\mathsf {E}}^4_2{\mathsf {C}}_0{\mathsf {c}}$ if the edge equilibria $E_{34}$ and $E_{24}$ are saturated and the others are not.
- The boundary index sum $\delta =0$ cannot occur for ${\mathsf {E}}^3_1{\mathsf {C}}_1{\mathsf {e}}$ if the edge equilibrium $E_{13}$ is saturated and the others are not.

The proof of (a) is given in “Appendix A.2”. The other statements can be proved in a similar manner.

#### Remark 4.3

- The extended boundary flow described in Theorem 4.2(d) is the reversed flow of the first extended boundary flow in Theorem 4.2(c).
- Table S1 informs us that there is exactly one potential extended boundary flow for each of the cases excluded by Theorem 4.2, except for ${\mathsf {E}}^4_2{\mathsf {C}}_0{\mathsf {c}}$ and $\delta =-1$. Then the two potentially possible extended boundary flows are the reversed flows of each other and thus both are excluded by Theorem 4.2.
- We could not exclude the boundary index sum $\delta =-1$ for the cases ${\mathsf {E}}^3_2{\mathsf {C}}_0{\mathsf {a}}$, ${\mathsf {E}}^3_1{\mathsf {C}}_1{\mathsf {a}}$, ${\mathsf {E}}^4_2{\mathsf {C}}_1{\mathsf {s}}$, ${\mathsf {E}}^4_3{\mathsf {C}}_0{\mathsf {e}}$, and ${\mathsf {E}}^4_1{\mathsf {C}}_2{\mathsf {e}}$. However, we conjecture that $\delta =-1$ does not occur (see Sect. 4.3).
- As a consequence of Theorem 4.2 and statement (b) of this remark, the number of potentially possible extended boundary flows reduces to 190.
- It is important to note that statements (c) and (d) of Theorem 4.2 exclude the existence of specific extended boundary flows and do not imply that the boundary index sum $\delta =0$ does not occur in these cases.
- The above theorem constrains the range (4.2) of possible values of $\delta$ to
  4.3 $$\begin{aligned} \max \{-1,k+m-n\} \le \delta \le k+m. \end{aligned}$$

An important tool for drawing conclusions about internal equilibria is the following index theorem:

#### Theorem 4.4

(Hofbauer 1990) Assume (2.5) and (${\mathcal {H}}$). Then

4.4 $$\begin{aligned} \sum _{\hat{x}\ \mathrm {saturated}} {\text {ind}}(\hat{x}) = +1, \end{aligned}$$

where the sum runs over all saturated equilibria.

This theorem has a number of important consequences. The simple proofs are left to the reader.

#### Corollary 4.5

Assume (2.5) and ($${\mathcal {H}}$$).

- The number of saturated equilibria is odd;
- If $\delta$ is odd (even), the number of internal equilibria is even (odd);
- The number of internal equilibria is at least $|1-\delta |$ and at most five;
- If $\delta >1$, there are at least $\delta -1$ internal saddle points;
- If $\delta <1$, the total number of sinks and sources is at least $1-\delta$;
- The boundary index sum $\delta$ is invariant under flow reversal.

### Established equilibrium structures and phase portraits

We show that 185 of the 190 theoretically possible extended boundary flows exist. This task is simplified by taking flow reversals into account.

Importantly, flow reversal neither changes the number of internal equilibria nor their index. Therefore, the boundary index sum $\delta$ is invariant under flow reversal; cf. Corollary 4.5(f). If we identify flow-reversal pairs, it is sufficient to consider $16+10$ boundary flow classes. Here, 16 is the number of representatives of boundary flows in Fig. 2 that are not invariant under flow reversal, and 10 is the number of boundary flows that are invariant. These 16 flows give rise to 79 theoretically possible extended boundary flows (SI, Table S1). The 10 self-inverse boundary flows give rise to 42 extended boundary flows, 22 of which form flow-reversal pairs (Table S1). Thus, up to flow reversal, there are $79+31$ different extended boundary flows. By Theorem 4.2, $2+5$ of these boundary flows cannot exist. Overall, we present phase portraits for $75+25$ extended boundary flows (Section S1 of the SI). Applying flow reversal, this proves existence of $2\times 75+35=185$ different extended boundary flows. All these flows have boundary index sum $\delta \ge 0$.

We could not realize $2+1$ potential extended boundary flows (five, including flow reversal), all of them having boundary index sum $\delta =-1$. Therefore, their existence remains undecided. For the following reasons, we conjecture that they do not exist. (i) Theorem 4.2(b) excludes the other five extended boundary flows with $\delta =-1$. (ii) A boundary index sum of $\delta =-1$ requires that the number of saddles in the interior is less than the total number of sinks and sources in the interior minus one. Despite extensive search no such equilibrium pattern could be found (see Section S1 of the SI).

Two flows yielding the same extended boundary flow (up to equivalence in the sense defined above) may still differ in their *equilibrium structure* because they differ in the number of internal equilibria or, if they have the same number of internal equilibria, in the number of sinks, sources, or saddles. In addition, some equilibrium structures can be realized by non-equivalent phase portraits; see below.

We show existence of an extended boundary flow or equilibrium structure by presenting a numerical fitness matrix ($m_{ij}$) yielding a flow on the entire state space, $[0,1]^2$, that has the given extended boundary flow (Section S1 of the SI). We represent each equivalence class of flows on $[0,1]^2$ by a phase portrait. In general, the phase portrait is not uniquely determined by the extended boundary flow, because already number and stability of internal equilibria are often not uniquely determined.

We present more than one phase portrait for several extended boundary flows. We do this for cases for which we found more than one equilibrium structure. In addition, we give examples of fitness matrices that yield the same equilibrium structure but non-equivalent phase portraits. One particularly interesting case is ${\mathsf {E}}^3_2{\mathsf {C}}_1{\mathsf {a}}$ with $\delta =1$, which admits three extended boundary flows and seven different phase portraits. One of these extended boundary flows has two equilibrium structures, each with two phase portraits (Fig. S2i, panels 1, 2, 3, 4). Another remarkable case is ${\mathsf {E}}^4_3{\mathsf {C}}_0{\mathsf {e}}$ with $\delta =1$, which admits four different extended boundary flows (Fig. S2j, panels 2, 3, 4, 5). However, there is also a considerable number of boundary flows for which the value of $\delta$ uniquely determines the extended boundary and also the equilibrium structure and the phase portrait (up to equivalence). See Table S1 and the phase portraits in Section S1 for detailed information.

### Permanence

In mathematical modeling of biological systems, the notion of *permanence* is very important. The dynamical system (2.5) is permanent if there exists a compact subset of $(0,1)^2$ such that every solution starting at $(p_0,q_0) \in (0,1)^2$ enters this subset and remains there (consult Hofbauer and Sigmund 1998 for an account on permanence theory). Permanence is equivalent to the boundary being a repeller. It can be shown that it is sufficient that all stationary points on the boundary are repelling.

We want to identify which extended boundary flows can be permanent. First, $\delta \ne 0$ implies that at least one boundary equilibrium is saturated and, therefore, attracts at least one orbit from the interior. Second, not all extended boundary flows with $\delta =0$ can be permanent, because there are extended boundary flows for which one equilibrium on the boundary is saturated with index $-1$ and an other has index $+1$. Therefore, an extended boundary flow is permanent if and only if no boundary equilibrium is saturated. Thus, we obtain:

#### Theorem 4.6

A full flow consistent with a boundary flow of type ${\mathsf {E}}_i^j{\mathsf {C}}_0{\mathsf {x}}$ is permanent if and only if no edge equilibrium is saturated.

#### Corollary 4.7

Assume (2.5) is permanent. Then

- the extended boundary flow is of one of the 14 classes ${\mathsf {E}}^1_1{\mathsf {C}}_0{\mathsf {e}}$, ${\mathsf {E}}^1_1{\mathsf {C}}_0{\mathsf {a}}$, ${\mathsf {E}}^2_2{\mathsf {C}}_0{\mathsf {c}}$, ${\mathsf {E}}^2_2{\mathsf {C}}_0{\mathsf {s}}$, ${\mathsf {E}}^2_2{\mathsf {C}}_0{\mathsf {e}}$, ${\mathsf {E}}^2_2{\mathsf {C}}_0{\mathsf {a}}$, ${\mathsf {E}}^2_1{\mathsf {C}}_0{\mathsf {e}}$, ${\mathsf {E}}^2_1{\mathsf {C}}_0{\mathsf {a}}$, ${\mathsf {E}}^3_3{\mathsf {C}}_0{\mathsf {e}}$, ${\mathsf {E}}^3_2{\mathsf {C}}_0{\mathsf {e}}$, ${\mathsf {E}}^3_2{\mathsf {C}}_0{\mathsf {a}}$, ${\mathsf {E}}^4_4{\mathsf {C}}_0{\mathsf {b}}$, ${\mathsf {E}}^4_3{\mathsf {C}}_0{\mathsf {e}}$, or ${\mathsf {E}}^4_2{\mathsf {C}}_0{\mathsf {c}}$, and has no saturated edge equilibrium;
- there exists an internal equilibrium that is a sink, for example the global maximum of the mean fitness.

#### Proof

- follows from Theorem 4.6 upon comparison with Fig. 2.
- follows because $\bar{m}$ is a (strict) Lyapunov function.$\square$

We note that each of the extended boundary flows in Corollary 4.7 could give rise to one, three, or five internal equilibria. However, the boundary flow ${\mathsf {E}}_4^4{\mathsf {C}}_0{\mathsf {b}}$ is the only one for which we found a fitness matrix such that (2.5) has five internal equilibria (see Fig. S1d, panel 6).

## Continuous isoclines: marginal overdominance or underdominance

In this and the next section we study which equilibrium structures and phase portraits are obtained by imposing specific properties on the isoclines. The structure of the two isoclines is important because the internal equilibria are their intersection points. As special cases, we will encounter well known models.

We start by investigating the equilibrium structure if the isoclines are continuous and map [0, 1] into (0, 1). From (3.11), we recall the definitions of marginal overdominance and underdominance.

### Lemma 5.1

The isocline *f*(*q*) (*g*(*p*)), defined in (3.4), is continuous and maps [0, 1] into (0, 1) if and only if locus *A* (*B*) exhibits either marginal overdominance or underdominance for every $q\in [0,1]$ $(p\in [0,1])$.

### Proof

From the definition of the isocline $p=f(q)$, we obtain that *f*(*q*) is continuous if and only if its denominator ($2m_{A_1A_2}-m_{A_1A_1}-m_{A_2A_2}$) does not change sign on [0, 1]. The requirement $0<f(q)<1$ is then satisfied if and only if both $m_{A_1A_2}-m_{A_1A_1}$ and $m_{A_1A_2}-m_{A_2A_2}$ have the same sign as the denominator. The argument for *g*(*p*) is analogous. $\square$

This lemma implies that the graph of *f*(*q*) intersects the boundary of the state space (precisely) at the equilibria $E_{24}$ and $E_{13}$, and the graph of *g*(*p*) does so at $E_{34}$ and $E_{12}$. If both isoclines are continuous and map [0, 1] into (0, 1), we conclude from Lemma 5.1 and Theorem 3.1 that the number of interior equilibria is between one and five, and both bounds can be assumed.

Next we want to derive the possible equilibrium structures if both loci exhibit marginal overdominance everywhere, i.e., at every $(p,q)\in [0,1]^2$. Obviously, this is a much stronger assumption than marginal overdominance at an equilibrium, as used in Corollary 3.3. We will need the following lemma:

### Lemma 5.2

Locus *A* exhibits marginal overdominance everywhere if and only if

5.1 $$\begin{aligned} m_{13}>\max \{m_{11},m_{33}\} \ \text {and}\ m_{24}>\max \{m_{22},m_{44}\}, \end{aligned}$$

i.e., overdominance holds on the edges $q=0$ and $q=1$, and

5.2 $$\begin{aligned} m_{14} >&\max \Bigl \{ m_{12} - \sqrt{(m_{13}-m_{11})(m_{24}-m_{22})}, \nonumber \\&m_{34} - \sqrt{(m_{13}-m_{33})(m_{24}-m_{44})} \Bigr \}. \end{aligned}$$

### Proof

We observe that

5.3 $$\begin{aligned} ax^2+2bx(1-x)+cx^2>0 \text { for every } x\in [0,1] \end{aligned}$$

if and only if $a>0$, $c>0$, and $b>-\sqrt{ac}$. Therefore, the conditions in the lemma follow immediately because $m_{A_1A_2}-m_{A_1A_1}$ and $m_{A_1A_2}-m_{A_2A_2}$ can be written in the form of (5.3) and need to be positive. $\square$

### Theorem 5.3

Assume that both loci exhibit marginal overdominance everywhere. Then the following holds:

- The boundary flow is of type ${\mathsf {E}}^4_4{\mathsf {C}}_0{\mathsf {b}}$.
- No boundary equilibrium is saturated, whence $\delta =0$ and the system is permanent.
- There may exist one, three, or five internal equilibria.
- If there is a unique internal equilibrium, it is globally asymptotically stable.
- If there are three internal equilibria, two are sinks and one is a saddle.
- If there are five internal equilibria, three are sinks and two are saddles.

### Proof

Statement (a) is clear from Lemma 5.2. Combining (3.11) with (A.1) and Theorem 4.6 yields (b). Statement (b) together with Corollary 4.5(b) gives claim (c). Theorem 4.4 yields the desired numbers of saddles for (d), (e), and (f). Because the trace of *J* (3.2) is negative due to (3.11), the internal equilibria with index $+1$ are sinks. $\square$

Phase portraits with one or three internal equilibria are shown in Fig. S1d, panels 4 and 5. We could neither find an example for case (f) in Theorem 5.3 nor could we exclude it.

### Theorem 5.4

Assume that both loci exhibit marginal underdominance everywhere. Then the following holds:

- The boundary flow is of type ${\mathsf {E}}^4_0{\mathsf {C}}_4{\mathsf {b}}$.
- All boundary equilibria are saturated, whence $\delta =0$.
- There may exist one, three, or five internal equilibria.
- If there is a unique internal equilibrium, it is a source.
- If there are three internal equilibria, two are sources and one is a saddle.
- If there are five internal equilibria, three are sources and two are saddles.

### Remark 5.5

The possible flows on $[0,1]^2$ for Theorem 5.4 are obtained by flow reversal from flows occurring by Theorem of 5.3.

Finally, the following can be shown. We leave the simple proof to the reader.

### Theorem 5.6

Assume one locus exhibits marginal overdominance everywhere and the other marginal underdominance everywhere.

- The boundary flow is of type ${\mathsf {E}}^4_2{\mathsf {C}}_0{\mathsf {c}}$.
- The two internally stable edge equilibria are saturated, whence $\delta =2$.
- There exists a unique internal equilibrium, and it is a saddle.

### Remark 5.7

It seems interesting that there exist fitness matrices that do not satisfy the assumption of the above theorem and generate a phase portrait with three internal equilibria and the boundary flow ${\mathsf {E}}^4_2{\mathsf {C}}_0{\mathsf {c}}$. Thus, the assumptions of marginal overdominance and underdominance everywhere do not only constrain the potential boundary flows substantially, but also the phase portraits that can occur for a given boundary flow.

## Linear isoclines

Here, we study the dynamics of systems obtained by fitness matrices yielding linear isoclines. This leads to a system of differential equations that has been investigated by Schuster et al. (1981) to study the evolution of two strategies in asymmetric animal contests, for example between two species. It also turns out to be equivalent to a model used by Zhivotovsky and Gavrilets (1992) to study quantitative genetic variation under epistatic selection. A number of well known models emerge as special cases, for instance the additive model or the haploid model.

It will be convenient to reparameterize the fitnesses $m_{ij}$ as follows:6.1Here, $a_i$ are allelic effects, $d_i$ dominance effects, and $e_{ij}$ epistatic effects. According to the remark below (2.7), one additional parameter could be specified (e.g., by setting $a_1=1$), but we refrain from doing so. The general assumption (${\mathcal {H}}$) implies that we exclude complete dominance or recessivity of an allele, i.e., $\left| d_i\right| \ne a_i$ for $i=1,2$, and analogous restrictions apply to $e_{12}$ and $e_{21}$.

A straightforward calculation shows that the dynamics (2.5) becomes

6.2a $$\begin{aligned} \dot{p}&= p(1-p) [a_1 + d_1(1-2p) + 2e_{22}q + 2(e_{12}-2e_{22})pq + (e_{21}-2e_{22})q^2 \nonumber \\&\qquad + (e_{11}-2e_{12}-2e_{21}+4e_{22})pq^2], \end{aligned}$$

6.2b $$\begin{aligned} \dot{q}&= q(1-q) [a_2 + d_2(1-2q) + 2e_{22}p + 2(e_{21}-2e_{22})pq + (e_{12}-2e_{22})p^2 \nonumber \\&\qquad + (e_{11}-2e_{12}-2e_{21}+4e_{22})p^2q]. \end{aligned}$$

We note that the right-hand side of (6.2a) is simply the additive effect of allele $A_1$ multiplied by *p*, and analogously the right-hand side of (6.2b) yields the additive effect of allele $B_1$.

### Remark 6.1

A standard decomposition of the total genetic variance (e.g., Kempthorne 1955) shows that dominance-by-dominance interactions are absent if and only if $e_{11}-2e_{12}-2e_{21}+4e_{22}=0$, additive-by-dominance interactions are absent if and only if

6.3 $$\begin{aligned} e_{11}=2e_{12}=2e_{21}=4e_{22}, \end{aligned}$$

additive-by-additive interactions are absent if and only if $e_{11}=e_{12}=e_{21}=e_{22}=0$, and dominance is absent if and only if $d_1=d_2=0$ and (6.3) holds.

We infer from (6.2) that both isoclines are linear if and only if (6.3) holds. With this assumption, (6.2) simplifies to

6.4a $$\begin{aligned} \dot{p}&= p(1-p) (a_1 + d_1 - 2d_1p + 2e_{22}q), \end{aligned}$$

6.4b $$\begin{aligned} \dot{q}&= q(1-q) (a_2 + d_2 - 2d_2q + 2e_{22}p). \end{aligned}$$

Interestingly, this is a model that was studied independently and in different contexts by Schuster et al. (1981) and Zhivotovsky and Gavrilets (1992). The latter authors introduced an *n*-locus version of the fitness scheme (6.1) with a generalized version of constraint (6.3), as ‘the simplest generalization of the additive model to include dominance and pairwise additive-by-additive epistasis’. Schuster et al. (1981) identified all possible (non-degenerate) phase portraits for (6.4). Because they were interested in a game-theoretical context, they studied a more general model in which one of the coefficients $e_{22}$ in (6.4) was substituted by a sixth, independent coefficient. Then limit cycles can occur because the corresponding $4\times 4$ fitness matrix $(m_{ij})$ is not symmetric. Here, we present the results of Schuster et al. (1981) that apply to our model (6.4) in our terminology and complement them. They also follow directly from our results in Sect. 4.

Since the isoclines are linear, there is either no or one internal equilibrium. Therefore, by Corollary 4.5(c), the boundary index sum can assume only the values $\delta =0,1$, or 2. Furthermore, if two edge equilibria exist on opposite edges, they must have the same internal stability. This rules out the following 10 of the 42 boundary flows in Fig. 2: ${\mathsf {E}}^2_1{\mathsf {C}}_0{\mathsf {e}}$, ${\mathsf {E}}^2_1{\mathsf {C}}_1{\mathsf {a}}'''$, ${\mathsf {E}}^2_1{\mathsf {C}}_2{\mathsf {e}}$, ${\mathsf {E}}^3_2{\mathsf {C}}_0{\mathsf {a}}$, ${\mathsf {E}}^3_2{\mathsf {C}}_1{\mathsf {a}}$, ${\mathsf {E}}^3_1{\mathsf {C}}_1{\mathsf {a}}$, ${\mathsf {E}}^3_1{\mathsf {C}}_2{\mathsf {a}}$, ${\mathsf {E}}^4_3{\mathsf {C}}_0{\mathsf {e}}$, ${\mathsf {E}}^4_1{\mathsf {C}}_2{\mathsf {e}}$, and ${\mathsf {E}}^4_2{\mathsf {C}}_1{\mathsf {s}}$. In addition, the following boundary flows are easily ruled out: ${\mathsf {E}}^1_1{\mathsf {C}}_0{\mathsf {a}},\ {\mathsf {E}}^1_0{\mathsf {C}}_1{\mathsf {a}},\ {\mathsf {E}}^2_2{\mathsf {C}}_0{\mathsf {a}},\ {\mathsf {E}}^2_0{\mathsf {C}}_2{\mathsf {a}},\ {\mathsf {E}}^2_1{\mathsf {C}}_0{\mathsf {a}}$ and ${\mathsf {E}}^2_1{\mathsf {C}}_1a''.$ Finally, the planar Routh–Hurwitz criterion implies that the internal equilibrium $(\hat{p},\hat{q})$ resulting from (6.4) is

- a saddle if $$d_1d_2<e_{22}^2$$;
- a sink if $d_1d_2>e_{22}^2$ and $d_1\hat{p}(1-\hat{p})+d_2\hat{q}(1-\hat{q})>0$; and
- a source if $d_1d_2>e_{22}^2$ and $d_1\hat{p}(1-\hat{p})+d_2\hat{q}(1-\hat{q})<0$.

We summarize the results:

### Corollary 6.2

Assume (6.4).

- The following eight boundary flows can have $\delta =0$: ${\mathsf {E}}^2_2{\mathsf {C}}_0{\mathsf {c}}$, ${\mathsf {E}}^2_2{\mathsf {C}}_0{\mathsf {s}}$, ${\mathsf {E}}^3_3{\mathsf {C}}_0{\mathsf {e}}$, ${\mathsf {E}}^4_4{\mathsf {C}}_0{\mathsf {b}}$ (in each case the internal equilibrium is globally attracting); ${\mathsf {E}}^2_0{\mathsf {C}}_2{\mathsf {c}}$, ${\mathsf {E}}^2_0{\mathsf {C}}_2{\mathsf {s}}$, ${\mathsf {E}}^3_0{\mathsf {C}}_3{\mathsf {e}}$, ${\mathsf {E}}^4_0{\mathsf {C}}_4{\mathsf {b}}$ (in each case the internal equilibrium is a source). The last four cases are the flow reversals of the first four cases.
- The following ten boundary flows and their flow reversals can have $\delta =1$: ${\mathsf {E}}^1_1{\mathsf {C}}_0{\mathsf {e}}$, ${\mathsf {E}}^1_1{\mathsf {C}}_1{\mathsf {e}}$, ${\mathsf {E}}^1_1{\mathsf {C}}_1{\mathsf {a}}$, ${\mathsf {E}}^2_2{\mathsf {C}}_0{\mathsf {c}}$, ${\mathsf {E}}^2_2{\mathsf {C}}_0{\mathsf {s}}$, ${\mathsf {E}}^2_2{\mathsf {C}}_0{\mathsf {e}}$ (if only $E_{12}$ is saturated), ${\mathsf {E}}^2_2{\mathsf {C}}_1{\mathsf {s}}$, ${\mathsf {E}}^2_1{\mathsf {C}}_1{\mathsf {a}}$ (if both edge equilibria are not saturated), ${\mathsf {E}}^3_3{\mathsf {C}}_0{\mathsf {e}}$ (if only $E_{13}$ is saturated), ${\mathsf {E}}^3_2{\mathsf {C}}_0{\mathsf {e}}$ (if only $E_{13}$ is saturated). In addition, ${\mathsf {E}}^0_0{\mathsf {C}}_1{\mathsf {s}}$ and ${\mathsf {E}}^0_0{\mathsf {C}}_1{\mathsf {a}}$ have $\delta =1$. In all these 22 cases, an internal equilibrium does not exist.
- The following five boundary flows and their flow reversals can have $\delta =2$: ${\mathsf {E}}^1_1{\mathsf {C}}_1{\mathsf {a}}$, ${\mathsf {E}}^2_2{\mathsf {C}}_0{\mathsf {c}}$, ${\mathsf {E}}^2_2{\mathsf {C}}_0{\mathsf {s}}$, ${\mathsf {E}}^2_1{\mathsf {C}}_1{\mathsf {a}}$, ${\mathsf {E}}^3_2{\mathsf {C}}_0{\mathsf {e}}$. In addition, ${\mathsf {E}}^0_0{\mathsf {C}}_2{\mathsf {b}}$ and ${\mathsf {E}}^4_2{\mathsf {C}}_0{\mathsf {c}}$ have $\delta =2$. In each of these twelve cases, the internal equilibrium is a saddle.

Therefore, a phase portrait is uniquely determined by its boundary flow and boundary index sum $\delta$. However, for 12 boundary flows, $\delta$ is not uniquely determined by the boundary flow. For ${\mathsf {E}}^2_2{\mathsf {C}}_0{\mathsf {c}}$, ${\mathsf {E}}^2_0{\mathsf {C}}_2{\mathsf {c}}$, ${\mathsf {E}}^2_2{\mathsf {C}}_0{\mathsf {s}}$, and ${\mathsf {E}}^2_0{\mathsf {C}}_2{\mathsf {s}}$, $\delta$ can assume all three possible values, 0, 1, and 2.

Zhivotovsky and Gavrilets (1992) derived results about the maintenance of a multilocus polymorphism, i.e., an internal equilibrium, and showed that several models investigated previously, mainly in a quantitative-genetic context, can be obtained as special cases of their model. Among others, they deduced the conditions for the existence and for the stability of a fully polymorphic equilibrium if all loci have equal effects on fitness and selection is sufficiently weak that linkage disequilibrium can be ignored. The assumption of equal effects is equivalent to a symmetric fitness matrix (2.2), i.e., $a_i=a$ and $d_i=d$ for every *i*.

A more detailed study of this symmetric model was performed by Gavrilets (1993). If the fitness matrix is symmetric ($a_i=a$ and $d_i=d$), the conditions for the existence and stability of an internal equilibrium become very simple. An internal (nondegenerate) equilibrium exists if and only if $0<\frac{a+d}{2(d-e_{22})}<1$. It exists and is linearly stable if and only if $-d<e_{22}<(d-a)/2$. This condition can be satisfied only if $d>a/3$. It also implies that there are internally stable equilibria on the edges $p=1$ and $q=1$. The corresponding boundary flow is ${\mathsf {E}}^2_2{\mathsf {C}}_0{\mathsf {s}}$ with $\delta =0$. We will briefly return to this model in Sect. 8, where we treat general symmetric fitness matrices.

Now we turn to two important special cases of the model (6.4).

### The additive fitness model

This classical model (Bodmer and Felsenstein 1967; Ewens 1969) assumes that loci contribute additively to fitness. Thus there is no epistasis, but dominance is admitted. It is obtained from (6.1) by assuming

6.5 $$\begin{aligned} e_{11} = e_{12} = e_{21} = e_{22} = 0. \end{aligned}$$

In fact, this model has been analyzed without ignoring linkage disequilibrium, i.e., the full four-gamete system (2.3), and indeed for any number of loci. Ewens (1969) proved for an arbitrary number of multiallelic loci that mean fitness is a Lyapunov function. Moreover, every equilibrium is in linkage equilibrium (for non-zero recombination, as we assume here). For diallelic loci, an internal equilibrium exists if and only if at every locus there is either overdominance or underdominance. An internal equilibrium is unique if it is isolated; it is globally asymptotically stable if there is overdominance on both loci (Karlin and Liberman 1978, 1990). All trajectories converge exponentially to an equilibrium on the linkage-equilibrium manifold $D=0$ (Lyubich 1992). For a more detailed review, see Bürger 2000 (pp. 48–50, 76–78). Therefore, the flows derived by assuming $D=0$ are representative for the full dynamics after a sufficiently long time has passed and also for every trajectory that starts close to $D=0$.

The isocline $\dot{p}=0$ simplifies to a horizontal straight line (in a representation as in Fig. 1), and the isocline $\dot{q}=0$ to a vertical straight line. It follows that locus *A* (*B*) exhibits marginal overdominance if and only if it exhibits overdominance on one of the respective edges, i.e., if and only if $d_1>a_1$ ($d_2>a_2$); analogously for underdominance. Therefore, in accordance with the above mentioned results, we obtain:

#### Corollary 6.3

For the additive fitness model the following boundary flows with boundary index sum $\delta$ occur:

- ${\mathsf {E}}^0_0{\mathsf {C}}_1{\mathsf {s}}$ with $\delta =1$ if dominance is intermediate at both loci, i.e., $-a_i<d_i<a_i$;
- ${\mathsf {E}}^2_2{\mathsf {C}}_0{\mathsf {e}}$ $({\mathsf {E}}^2_0{\mathsf {C}}_2{\mathsf {e}})$ with $\delta =1$ if dominance is intermediate at one locus and the other locus is overdominant (underdominant);
- ${\mathsf {E}}^4_4{\mathsf {C}}_0{\mathsf {b}}$ $({\mathsf {E}}^4_0{\mathsf {C}}_4{\mathsf {b}})$ with $\delta =0$ if both loci are overdominant (underdominant);
- ${\mathsf {E}}^4_2{\mathsf {C}}_0{\mathsf {c}}$ with $\delta =2$ if one locus is overdominant, the other underdominant.

An internal equilibrium exists and is unique for the boundary flows ${\mathsf {E}}^4_4{\mathsf {C}}_0{\mathsf {b}}$, ${\mathsf {E}}^4_0{\mathsf {C}}_4{\mathsf {b}}$, and ${\mathsf {E}}^4_2{\mathsf {C}}_0{\mathsf {c}}$. It is globally attracting, a source, and a saddle, respectively. Each of the above boundary flows admits only one phase portrait.

### The haploid model

If selection acts on haploids instead of diploids, fitnesses can be assigned directly to gametes. Denoting the fitness of gamete *i* by $v_i$, straightforward calculations show that under the assumption $D=0$ the following dynamics is obtained (cf. Haldane 1931):

6.6a $$\begin{aligned} \dot{p}&= p(1-p) [v_2-v_4 + (v_1-v_2-v_3+v_4)q], \end{aligned}$$

6.6b $$\begin{aligned} \dot{q}&= q(1-q) [v_3-v_4 + (v_1-v_2-v_3+v_4)p]. \end{aligned}$$

Comparison with (6.4) reveals that this is obtained from the fitness scheme (6.1) if one sets $d_1=d_2=0$, $v_4=0$ (without loss of generality), $v_3=a_2$, $v_2=a_1$, $v_1=a_1+a_2+2e_{22}$, and assumes (6.3). Therefore, there is additive-by-additive epistasis but no dominance.

The isocline $\dot{p}=0$ is given by the vertical line $q=a_1/(2e_{22})$, and the isocline $\dot{q}=0$ is given by the horizontal line $p=a_2/(2e_{22})$. Hence, there are no edge equilibria, but there may exist one internal equilibrium given by these values. The only possible boundary flows are ${\mathsf {E}}^0_0{\mathsf {C}}_1{\mathsf {s}}$, ${\mathsf {E}}^0_0{\mathsf {C}}_1{\mathsf {a}}$, and ${\mathsf {E}}^0_0{\mathsf {C}}_2{\mathsf {b}}$. Their boundary index sum is uniquely determined and $\delta =1$, 1, and 2, respectively. It is easy to show that all three cases can be realized. An internal equilibrium exists only in the third case, and it is a saddle. These results are in accordance with results obtained previously for the following more general models.

In game theory, (6.6) is known as the replicator dynamics for $2\times 2$ partnership games, which is generalized by the replicator dynamics for $2\times 2$ bimatrix games. There, the coefficients of *p* and *q* in (6.6) may differ. Schuster and Sigmund (1981) proved that periodic orbits occur if these coefficients have opposite signs (see Hofbauer and Sigmund 1998, Sections 10 and 11, for a treatment of bimatrix games and the replicator dynamics).

The complete haploid selection model, i.e., without the assumption of linkage equilibrium, was investigated by Felsenstein (1965), Feldman (1971), Rutschman (1994), and Bank et al. (2012). The latter authors proved that there exists at most one internal equilibrium and, if it exists, it is unstable. Then two vertex equilibria are asymptotically stable. This corresponds to the boundary flow ${\mathsf {E}}^0_0{\mathsf {C}}_2{\mathsf {b}}$. Otherwise, one vertex is globally asymptotically stable and the boundary flow is either ${\mathsf {E}}^0_0{\mathsf {C}}_1{\mathsf {s}}$ or ${\mathsf {E}}^0_0{\mathsf {C}}_1{\mathsf {a}}$. Bank et al. (2012) also identified the parameter combinations that lead to the respective equilibrium structures.

## The multilinear epistasis model

Hansen and Wagner (2001) introduced a model of gene interaction that assumes that the effect of gene substitutions due to changes in the genetic background (the other loci) can be described by a linear transformation. Although their model is formulated in terms of genetic effects on quantitative traits, it can be applied to our context if we consider fitness as the trait. A two-locus version of this model, and its applications to the maintenance of genetic variation, was studied by Hermisson et al. (2003). Following their formulation, the fitness of the two-locus genotype $A_iA_jB_kB_\ell$ can be written as

7.1 $$\begin{aligned} w(ij,k\ell ) = \mu + \alpha (ij) + \beta (k\ell ) + \gamma \alpha (ij)\beta (k\ell ). \end{aligned}$$

Comparison with the fitness scheme (6.1) shows, after some calculation, that their model is the special case of (6.1) obtained by assuming

7.2 $$\begin{aligned} e_{11} = \frac{4a_1a_2e_{22}}{(a_1+d_1)(a_2+d_2)}, \text { and } e_{12} = \frac{2a_1e_{22}}{a_1+d_1}, \text { and } e_{21}=\frac{2a_2e_{22}}{a_2+d_2}. \end{aligned}$$

It follows that the isoclines $\partial {\bar{m}}/\partial p=0$ and $\partial {\bar{m}}/\partial q=0$ take the form

7.3a $$\begin{aligned}&\frac{(a_2+d_2-2d_2p)\varphi _2(q)}{(a_1+d_1)(a_2+d_2)} = 0, \end{aligned}$$

7.3b $$\begin{aligned}&\frac{(a_1+d_1-2d_1q)\varphi _1(p)}{(a_1+d_1)(a_2+d_2)} = 0, \end{aligned}$$

where

7.3c $$\begin{aligned} \varphi _i(x) = (a_1+d_1)(a_2+d_2)+2e_{22}x(a_i+d_i-d_ix). \end{aligned}$$

Therefore, they are in product form. It is not difficult to show that the isoclines of (6.2) are in product form if and only if (7.2) holds. In this case, which we assume below, the dynamics has separated variables.

We define

7.4 $$\begin{aligned} {\tilde{p}}= \frac{a_2+d_2}{2d_2} \quad \text {and}\quad {\tilde{q}} = \frac{a_1+d_1}{2d_1}, \end{aligned}$$

and observe that $0<{\tilde{p}}<1$ ($0<{\tilde{q}}<1$) if and only if there is overdominance or underdominance at locus *A* (*B*). If $0<{\tilde{p}}<1$ and $0<{\tilde{q}}<1$, we call the equilibrium $({\tilde{p}},{\tilde{q}})$ the central equilibrium. We note that $p={\tilde{p}}$ and $q={\tilde{q}}$ are invariant lines of the dynamics (6.2) with (7.2). As a consequence, saddle connections occur robustly in this class, e.g., for the boundary flow ${\mathsf {E}}^4_4{\mathsf {C}}_0{\mathsf {b}}$ with $\delta =2$ (Fig. S3h, panel 6).

The isoclines may be composed of up to three straight lines. For $\dot{p}=0$ (and in a representation as in Fig. 1), these are the horizontal line $p={\tilde{p}}$ and the two vertical lines that are given by the solutions of $\varphi _2(q)=0$. If $0<{\tilde{p}}<1$, then the two edge equilibria $E_{13}$ and $E_{24}$ exist and have the same value, ${\tilde{p}}$. Thus, edge equilibria occur always in pairs on opposite edges. If in addition $0<{\tilde{q}}<1$, the other two edge equilibria and the central equilibrium $({\tilde{p}}, {\tilde{q}})$ exist. Furthermore, the intersection points of $\varphi _2(q)=0$ and $\varphi _1(p)=0$ may yield up to four additional internal equilibria. Thus, in total there may be up to five internal equilibria.

The case of five internal equilibria can be realized for overdominance at both loci as well as for underdominance at both loci, but not if one locus exhibits overdominance and the other underdominance (Theorem 7.1 and Table S3). The corresponding boundary flows are ${\mathsf {E}}^4_4{\mathsf {C}}_0{\mathsf {b}}$ with $\delta =4$ and its flow reversal ${\mathsf {E}}^4_0{\mathsf {C}}_4{\mathsf {b}}$ .

Because opposite edge equilibria occur pairwise, flows of type ${\mathsf {E}}^1_m$ or ${\mathsf {E}}^3_m$ cannot occur. The following theorem lists all possible equilibrium structures. Only 16 of the 42 boundary flows in Fig. 2 can occur. In particular, it states the extent to which the equilibrium structure can be inferred from the boundary flow. The proof is given in “Appendix A.3”. Parameter combinations that yield all possible equilibrium structures are given in Table S3.

### Theorem 7.1

Assume (6.2) and (7.2).

- All possible equilibrium structures in the interior are given in Table 1.
- For the top twelve boundary-flow classes ${\mathsf {E}}^n_m{\mathsf {C}}_k{{\mathsf {x}}}$ in Table 1, the number of asymptotically stable edge equilibria is $\min \{m,\delta \}$. For the other four classes, this is in general wrong.

**Table 1:** Internal equilibrium structures for the multilinear epistasis model

Boundary flow	$\delta =0$	$\delta =1$	$\delta =2$	$\delta =3$	$\delta =4$
${\mathsf {E}}^0_0{\mathsf {C}}_1{\mathsf {s}}$	−	0	−	−	−
${\mathsf {E}}^0_0{\mathsf {C}}_1{\mathsf {a}}$	−	0	−	−	−
${\mathsf {E}}^0_0{\mathsf {C}}_2{\mathsf {b}}$	−	−	1 saddle	−	−
${\mathsf {E}}^2_2{\mathsf {C}}_0{\mathsf {c}}$	−	0	−	−	−
${\mathsf {E}}^2_0{\mathsf {C}}_2{\mathsf {c}}$	−	0	−	−	−
${\mathsf {E}}^2_2{\mathsf {C}}_0{\mathsf {e}}$	−	$0^*$	−	−	−
${\mathsf {E}}^2_0{\mathsf {C}}_2{\mathsf {e}}$	−	$0^*$	−	−	−
${\mathsf {E}}^2_1{\mathsf {C}}_0{\mathsf {e}}$	−	0	−	−	−
${\mathsf {E}}^2_1{\mathsf {C}}_1{\mathsf {a}}'''$	−	−	1 saddle	−	−
${\mathsf {E}}^2_1{\mathsf {C}}_2{\mathsf {e}}$	−	0	−	2 saddles	−
${\mathsf {E}}^4_4{\mathsf {C}}_0{\mathsf {b}}$	1 sink	−	1 saddle	−	1 source, 4 saddles
${\mathsf {E}}^4_0{\mathsf {C}}_4{\mathsf {b}}$	1 source	−	1 saddle	−	1 sink, 4 saddles
${\mathsf {E}}^4_3{\mathsf {C}}_0{\mathsf {e}}$	1 sink	−	1 saddle or 1 source and 2 saddles	−	−
${\mathsf {E}}^4_1{\mathsf {C}}_2{\mathsf {e}}$	1 source	−	1 saddle or 1 sink and 2 saddles	−	−
${\mathsf {E}}^4_2{\mathsf {C}}_0{\mathsf {c}}$	1 sink or 1 source	−	1 saddle	−	−
${\mathsf {E}}^4_2{\mathsf {C}}_1{\mathsf {s}}$	−	1 sink or source, 1 saddle	−	−	−

A ‘−’ indicates that this value of $\delta$ does not occur, and ‘0’ indicates that the number of internal equilibria is zero. A comma means ‘and’. ${}^*$ Indicates that this case can be realized by matrices with different extended boundary flows; thus there are two different equilibrium structures. For the four boundary flows at the bottom, which admit two different internal equilibrium structures for one value of $\delta$, each of the internal equilibrium structures is generated by a different, but unique, extended boundary flow

### Remark 7.2

Because mean fitness is a strict Lyapunov function (Sect. 2), there is a globally asymptotically stable equilibrium if and only if there is precisely one sink. This occurs in the following cases: ${\mathsf {E}}^0_0{\mathsf {C}}_1{\mathsf {s}}$ and ${\mathsf {E}}^0_0{\mathsf {C}}_1{\mathsf {a}}$ (a corner equilibrium is globally attracting); ${\mathsf {E}}^2_2{\mathsf {C}}_0{\mathsf {c}}$, ${\mathsf {E}}^2_2{\mathsf {C}}_0{\mathsf {e}}$, and ${\mathsf {E}}^2_1{\mathsf {C}}_0{\mathsf {e}}$ (an edge equilibrium is globally attracting); ${\mathsf {E}}^4_4{\mathsf {C}}_0{\mathsf {b}}$ and ${\mathsf {E}}^4_3{\mathsf {C}}_0{\mathsf {e}}$, each with $\delta =0$ (the central equilibrium is globally attracting); ${\mathsf {E}}^4_2{\mathsf {C}}_0{\mathsf {c}}$ with $\delta =0$ (in this case the central equilibrium is a sink, hence globally attracting).

We observe from (7.2) that the multilinear model reduces to that with linear isoclines in Sect. 6 if and only if $d_1=d_2=0$ or $e_{22}=0$. Thus, the multilinear model of epistasis coincides with the epistatic model of Zhivotovsky and Gavrilets (1992) if and only if dominance is absent, when it simplifies to the haploid model (Sect. 6.2), or if epistasis is absent, when it reduces to the additive model (Sect. 6.1). It is easy to show that if dominance and epistasis are present, the multilinear model and that of Zhivotovsky and Gavrilets are different and none is a special case of the other. The reason is that in the model of Zhivotovsky and Gavrilets there are no additive-by-dominance or dominance-by-dominance interactions, whereas they occur in the multilinear model if dominance is present (as follows from Eq. 7.2 and Remark 6.1).

### Remark 7.3

- The following (extended) boundary flows occur in the multilinear epistasis model but not in the model of Zhivotovsky and Gavrilets (1992): ${\mathsf {E}}^2_1{\mathsf {C}}_0{\mathsf {e}}$, ${\mathsf {E}}^2_1{\mathsf {C}}_1{\mathsf {a}}'''$, ${\mathsf {E}}^2_1{\mathsf {C}}_2{\mathsf {e}}$, ${\mathsf {E}}^4_3{\mathsf {C}}_0{\mathsf {e}}$, ${\mathsf {E}}^4_1{\mathsf {C}}_2{\mathsf {e}}$, ${\mathsf {E}}^4_2{\mathsf {C}}_1{\mathsf {s}}$; ${\mathsf {E}}^4_4{\mathsf {C}}_0{\mathsf {b}}$ and ${\mathsf {E}}^4_0{\mathsf {C}}_4{\mathsf {b}}$ (each with $\delta =2$ and $\delta =4$) and ${\mathsf {E}}^4_2{\mathsf {C}}_0{\mathsf {c}}$ with $\delta =0$ (see Table S2).
- The extended boundary flows that occur both in the multilinear model and in the model of Zhivotovsky and Gavrilets are those occuring in the haploid model (no edge equilibria), those occuring in the absence of dominance and epistasis (listed in Corollary 6.3), and ${\mathsf {E}}^2_2{\mathsf {C}}_0{\mathsf {c}}$ and ${\mathsf {E}}^2_0{\mathsf {C}}_2{\mathsf {c}}$, each with $\delta =1$ (see Table S2). However, in the latter case the fitness matrices are different.

Finally, we note that the above model was investigated by Bomze et al. (1983) in the context of Mendelian game dynamics of asymmetric contests. Because then the fitness matrix (2.2) is in general not symmetric, periodic solutions may occur. Bomze (1982) provided a classification of all possible phase portraits.

## Equal locus effects

If both loci contribute to fitness equally, the $3\times 3$ matrix in (6.1) is symmetric, i.e.,

8.1 $$\begin{aligned} a_1 = a_2 = a, \; d_1 = d_2 = d, \;\text {and}\; e_{21} = e_{12}. \end{aligned}$$

Under this assumption, which we impose throughout this section, only the nine boundary flows in Fig. 2 occur that have a code ending by an ${\mathsf {s}}$ or ${\mathsf {b}}$. However, additional restrictions on the possible phase portraits and equilibrium structures occur as we will demonstrate now.

The symmetry implies that edge equilibria occur pairwise, i.e., ($E_{24},\ E_{34}$) and ($E_{12},\ E_{13}$). Equilibria of a pair have the same internal and the same external stability. Therefore, boundary flows with an odd number of stable corner equilibria give rise to odd values $\delta$; with an even number of stable corner equilibria $\delta$ is even.

Using the symmetry (8.1), we infer from (6.2) that the isoclines take the form

8.2 $$\begin{aligned} h(x)=\frac{a + d + 2e_{22}x + (e_{12} -2e_{22})x^2}{2d -2(e_{12} -2 e_{22}) x - (e_{11} - 4e_{12} + 4e_{22}) x^2}, \end{aligned}$$

where for the $\dot{p}=0$ isocline we have $h(p)=q$, and for the $\dot{q}=0$ isocline we have $h(q)=p$. Therefore, we obtain up to three symmetric internal equilibria. They satisfy $p=q$ and are the solutions of the cubic equation $h(x)=x$. In addition, there may exist a pair of internal equilibria that are mirror images of each other with respect to the diagonal $p=q$. Since the internal equilibria are the zeros of the quintic polynomial (3.7), this pair of equilibria is given by the zeros of a quadratic polynomial.

The following theorem determines the number of internal equilibria for all possible boundary flows. We recall from Corollary 4.5 that the number of internal equilibria is odd if and only if $\delta$ is even.

### Theorem 8.1

Assume (6.2) and (8.1). Possible boundary flows are of class ${\mathsf {E}}^{2n}_{2m}{\mathsf {C}}_k{\mathsf {s}}$, where $n,m\in \{0,1,2\}$, $m\le n$, and $k\in \{0,1,2,3,4\}$. The boundary index sum $\delta$ is odd for boundary-flow classes with an odd number of stable corner equilibria, and $\delta$ is even otherwise. In addition, the following holds:

- For the boundary-flow classes ${\mathsf {E}}^2_2{\mathsf {C}}_1{\mathsf {s}}$ and ${\mathsf {E}}^2_0{\mathsf {C}}_3{\mathsf {s}}$, each with $\delta =1$ or $\delta =3$, and for ${\mathsf {E}}^4_2{\mathsf {C}}_1{\mathsf {s}}$ with $\delta =3$, the number of internal equilibria can be zero (only for $\delta =1$), two, or four.
- For the boundary-flow classes ${\mathsf {E}}^4_4{\mathsf {C}}_0{\mathsf {b}}$ and ${\mathsf {E}}^4_0{\mathsf {C}}_4{\mathsf {b}}$, each with $\delta =0$ or $\delta =4$, the number of internal equilibria can be one (only for $\delta =1$), three, or five.
- For the boundary-flow classes ${\mathsf {E}}^0_0{\mathsf {C}}_1{\mathsf {s}}$ (which has $\delta =1$), ${\mathsf {E}}^0_0{\mathsf {C}}_2{\mathsf {b}}$ (which has $\delta =2$), ${\mathsf {E}}^2_2{\mathsf {C}}_0{\mathsf {s}}$ and ${\mathsf {E}}^2_0{\mathsf {C}}_2{\mathsf {s}}$ (which both have $\delta =0$ or $\delta =2$), ${\mathsf {E}}^4_4{\mathsf {C}}_0{\mathsf {b}}$ and ${\mathsf {E}}^4_0{\mathsf {C}}_4{\mathsf {b}}$ with $\delta =2$, and ${\mathsf {E}}^4_2{\mathsf {C}}_1{\mathsf {s}}$ with $\delta =1$, the respective maximum number of internal equilibria (four or five) cannot be assumed. Any smaller number of internal equilibria admitted by Corollary 4.5 (1 or 3 if $\delta =0$ or $\delta =2$, 0 or 2 if $\delta =1$) can be assumed.

The proof is given in “Appendix A.4”. Without proof (which is simple), we note that in this symmetric case, $\delta =-1$ can be excluded for ${\mathsf {E}}^4_2{\mathsf {C}}_1{\mathsf {s}}$. Therefore, the only possible values are $\delta =1$ and $\delta =3$.

Finally, we briefly treat two special cases. For the model of Gavrilets (1993) mentioned in Sect. 6, i.e., Eq. (6.4) with (8.1), the following boundary flows and values $\delta$ can occur: All eight boundary flows from Corollary 6.2 ending with an ${\mathsf {s}}$ or ${\mathsf {b}}$ occur. For ${\mathsf {E}}^0_0{\mathsf {C}}_1{\mathsf {s}}$, ${\mathsf {E}}^0_0{\mathsf {C}}_2{\mathsf {b}}$, ${\mathsf {E}}^2_2{\mathsf {C}}_1{\mathsf {s}}$, ${\mathsf {E}}^2_0{\mathsf {C}}_3{\mathsf {s}}$, ${\mathsf {E}}^4_4{\mathsf {C}}_0{\mathsf {b}}$, and ${\mathsf {E}}^4_0{\mathsf {C}}_4{\mathsf {b}}$, the boundary index sum is already uniquely determined ($\delta =1$, 2, 1, 1, 0, and 0, respectively). For ${\mathsf {E}}^2_2{\mathsf {C}}_0{\mathsf {s}}$ and ${\mathsf {E}}^2_0{\mathsf {C}}_2{\mathsf {s}}$ the case $\delta =1$ is easily excluded, whence only $\delta =0$ or $\delta =2$ are possible.

If the symmetry assumption (8.1) is imposed on the multilinear epistasis model treated in Sect. 7, the following boundary flows and equilibrium structures occur.

${\mathsf {E}}^0_0{\mathsf {C}}_1{\mathsf {s}}$ ($\delta =1$) and ${\mathsf {E}}^0_0{\mathsf {C}}_2{\mathsf {b}}$ ($\delta =2$); in both cases, the equilibrium structure is unique.

${\mathsf {E}}^4_2{\mathsf {C}}_1{\mathsf {s}}$ ($\delta =1$): both cases (sink and saddle, source and saddle) can be realized.

${\mathsf {E}}^4_4{\mathsf {C}}_0{\mathsf {b}}$ and ${\mathsf {E}}^4_0{\mathsf {C}}_4{\mathsf {b}}$: each with $\delta =0$ and $\delta =4$. $\delta =2$ cannot occur because this would require neighboring edge equilibria to differ in their external stability.

Interestingly, there are no equilibrium structures with two edge equilibria in the multilinear model if loci have equal effects.

## The symmetric viability model

As already outlined in the Introduction, the so-called symmetric viability model has received much attention in the literature. One reason is that special cases of it arise naturally when two diallelic loci determine a quantitative character that is under stabilizing selection toward an intermediate optimum (e.g., Wright 1935, 1952; Hastings 1987; Nagylaki 1989; Gavrilets and Hastings 1993; Bürger and Gimelfarb 1999; Willensdorfer and Bürger 2003). For a detailed review, consult Bürger (2000, Chap. 6.2).

It has the property that fitnesses of genotypes are invariant under the simultaneous exchange of $A_1$ with $A_2$ and $B_1$ with $B_2$. Therefore the resulting fitness matrix (2.2) is centro-symmetric and depends only on four parameters. We shall use the following parametrization (cf. Nagylaki 1989):9.1This is equivalent to the most general form, as first introduced by Bodmer and Felsenstein (1967). The model studied by Lewontin and Kojima (1960) corresponds to the special case $m=0$. Interestingly, this is also a special case of the multilinear epistasis model treated in Sect. 7 [by setting $a_1=a_2=e_{11}=e_{12}=e_{21}=0$ in (6.1)]. Bodmer and Parson (1962) assumed $r_1=r_2$, which makes the matrix symmetric, in addition to being centro-symmetric. Thus, only boundary flows ending with ${\mathsf {b}}$ can be realized. The most general models of stabilizing selection (among those referred to above) require all four parameters in (9.1). However, because the double heterozygote has the highest fitness and fitness of trait values decays symmetrically with distance from the optimum, the four parameters have to satisfy certain inequalities (see Nagylaki 1989).

We analyze the general model (9.1). Instead of $(p,q)\in [0,1]^2$, we use the coordinates $(x,y)\in [-1,1]^2$ defined by

9.2 $$\begin{aligned} p=\frac{1+x}{2}, \; q=\frac{1+y}{2}. \end{aligned}$$

This transforms the system (2.5) into

9.3a $$\begin{aligned} \dot{x}&=(1-x^2)(r_1x+my+lxy^2), \end{aligned}$$

9.3b $$\begin{aligned} \dot{y}&=(1-y^2)(r_2y+mx+lyx^2), \end{aligned}$$

which will form the basis for the subsequent analysis.

It is immediate that the origin $O=(0,0)$ is an equilibrium and the dynamics is point-symmetric with respect to it. In particular, if $(\hat{x}, \hat{y})$ is an equilibrium of (9.3), so is $(-\hat{x}, -\hat{y})$, and both have the same eigenvalues because the respective Jacobian matrices are equal. We observe that the isoclines of (9.3) are in product form if $m=0$, whence a special case of the multilinear epistasis model emerges. The isoclines are linear if $l=0$.

If the isoclines are not linear, they are given by

9.4a $$\begin{aligned} x = \psi _1(y)&= \frac{-my}{r_1+ly^2}, \end{aligned}$$

9.4b $$\begin{aligned} y = \psi _2(x)&= \frac{-mx}{r_2+lx^2}. \end{aligned}$$

### Theorem 9.1

- The central, or symmetric, equilibrium *O* exists always.
- If $l\ne 0$ and $r_1r_2\ne 0$, the following pairs of unsymmetric internal equilibria may exist:
  9.5a $$\begin{aligned} y_{1,2}=&\pm \sqrt{\frac{-r_1-m\sqrt{\frac{r_1}{r_2}}}{l}},\quad x_{1,2}=\sqrt{\frac{r_2}{r_1}}y_{1,2}, \end{aligned}$$
  9.5b $$\begin{aligned} y_{3,4}=&\pm \sqrt{\frac{-r_1+m\sqrt{\frac{r_1}{r_2}}}{l}},\quad x_{3,4}=-\sqrt{\frac{r_2}{r_1}}y_{3,4}. \end{aligned}$$

In particular, the following holds:

(i) If ${\text {sgn}}r_1 = {\text {sgn}}r_2 = {\text {sgn}}l$, then at most one of these pairs is admissible.
(ii) If ${\text {sgn}}r_1 = {\text {sgn}}r_2 = -{\text {sgn}}l$, then both pairs may be admissible.
(iii) If ${\text {sgn}}r_1 = -{\text {sgn}}r_2$, then *O* is the only internal equilibrium.

The *proof* is given in “Appendix A.5.1”.

It is straightforward to compute the Jacobian of (9.3). At an internal equilibrium $(\hat{x},\hat{y})$ it simplifies to

9.6 $$\begin{aligned} J(\hat{x},\hat{y})=\begin{pmatrix} (1-\hat{x}^2)(r_1+l\hat{y}^2) &{} (1-\hat{x}^2)(m+2l\hat{x} \hat{y})\\ (1-\hat{y}^2)(m+2l\hat{x} \hat{y}) &{} (1-\hat{y}^2)(r_2+l\hat{x}^2) \end{pmatrix}; \end{aligned}$$

cf. Lemma 3.2. Evaluation at the central equilibrium *O* yields

9.7 $$\begin{aligned} J_O = \begin{pmatrix} r_1 &{}\quad m\\ m &{}\quad r_2 \end{pmatrix}. \end{aligned}$$

From the Routh–Hurwitz criterion we infer immediately:

### Lemma 9.2

Let

9.8a $$\begin{aligned} \rho = \det {J_O}&=r_1r_2-m^2, \end{aligned}$$

9.8b $$\begin{aligned} {\text {tr}}J_O&=r_1+r_2 . \end{aligned}$$

Then *O* is

- a saddle with index $-1$ if $\rho <0$,
- a sink with index $+1$ if $\rho >0$ and ${\text {tr}}J_O<0$,
- a source with index $+1$ if $\rho >0$ and ${\text {tr}}J_O>0$.

Note that the sign of ${\text {tr}}J_O$ can be determined immediately from the fitness scheme (9.1). If the fitness of the double heterozygous genotpye $A_1A_2B_1B_2$ exceeds the arithmetic mean fitness of, for instance, the homozygous genotypes $A_iA_iB_1B_1$ and $A_iA_iB_2B_2$ ($i=1$ or 2), then ${\text {tr}}J_O<0$.

### Remark 9.3

- Lemma 9.2 settles the internal equilibrium structure for the case $l=0$, in which *O* is the only internal equilibrium.
- Let $r_1r_2=0$. If $r_1=r_2=0$, the equilibrium ${\mathsf {O}}$ is a saddle, and there exists either no other internal equilibrium (if $\left| m/l\right| >1$) or the curve of equilibria $y=-m/(lx)$, yielding a degenerate flow. If $r_1=m=0$, then $y=0$ is a line of equilibria, hence again degenerate. If $r_1=0$ and $m\ne 0$ and $l\ne 0$, then *O* is the unique internal equilibrium, and it is a saddle.

For the remainder of this section, we assume $l\ne 0$ and $r_1r_2\ne 0$. As a corollary to the above theorem, we obtain

### Corollary 9.4

If ${\text {sgn}}r_2=-{\text {sgn}}r_1$, then *O* is the unique internal equilibrium. It is a saddle point and $\delta =2$.

### Proof

We already know from Theorem 9.1 that *O* is unique. If ${\text {sgn}}r_2=-{\text {sgn}}r_1$, then $\rho <0$ and *O* is a saddle. Since the index of a saddle point is $-1$, Theorem 4.4 shows that the boundary index sum is $\delta =2$. $\square$

Here is the main result of this section.

### Theorem 9.5

- The internal equilibrium structures that can occur in the symmetric viability model are given in Table 2.
- The stability of the boundary equilibria can be inferred from the boundary flow type ${\mathsf {E}}^n_m{\mathsf {C}}_k$ as follows:
- The number of asymptotically stable corner equilibria is *k*.
- The number of asymptotically stable edge equilibria is $\min \{m,\delta \}$.

**Table 2:** Internal equilibrium configurations for the symmetric viability model

Boundary flow	$\delta =0$		$\delta =2$		$\delta =4$	
	$\rho <0$	$\rho >0$	$\rho <0$	$\rho >0$	$\rho <0$	$\rho >0$
${\mathsf {E}}^0_0{\mathsf {C}}_2{\mathsf {b}}$	−	−	1 saddle	1 sink or source$^b$, 2 saddles	−	−
${\mathsf {E}}^2_2{\mathsf {C}}_0{\mathsf {c}}$	1 saddle, 2 sinks	1 sink	1 saddle	1 sink or source$^a$, 2 saddles	−	−
${\mathsf {E}}^2_0{\mathsf {C}}_2{\mathsf {c}}$	1 saddle, 2 sources	1 source	1 saddle	1 sink or source$^a$, 2 saddles	−	−
${\mathsf {E}}^4_4{\mathsf {C}}_0{\mathsf {b}}$	1 saddle, 2 sinks	1 sink	1 saddle	1 source, 2 saddles	3 saddles	1 source, 4 saddles
${\mathsf {E}}^4_0{\mathsf {C}}_4{\mathsf {b}}$	1 saddle, 2 sources	1 source	1 saddle	1 sink, 2 saddles	3 saddles	1 sink, 4 saddles
${\mathsf {E}}^4_2{\mathsf {C}}_0{\mathsf {c}}$	$\times$	1 sink or 1 source$^b$	1 saddle	1 sink or source$^b$, 2 saddles	−	−

A ‘−’ indicates that this value of $\delta$ does not occur (see Table S1) and ‘$\times$’ indicates that this combination of $\rho$ and $\delta$ cannot occur. A comma means ‘and’. ${}^a$ Whether *O* is a sink or a source needs to be determined from the sign of $r_1+r_2$. ${}^b$ The stability of *O* switches under flow reversal, which (in these classes) does not alter the extended boundary-flow class

The *proof* is given in “Appendix A.5.2”. Examples of all possible flows can be found in Section S1 of the SI.

From Table 2, we infer immediately that *O* is the unique internal equilibrium if either $\delta =0$ and $\rho >0$ or if $\delta =2$ and $\rho <0$. In fact, the table shows that the number of internal equilibria can always be determined from the extended boundary flow and $\rho$.

Nagylaki (1989) identified all equilibrium structures and phase portraits for the special case of the symmetric viability model that arises from stabilizing selection toward an optimum situated on the genotypic value of the double heterozygote. He admitted arbitrary functions decaying from the optimum monotonically and symmetrically, and he assumed absence of linkage equilibrium. Thus, his model is a special cases of (9.3) (after transformation between (*p*, *q*) and (*x*, *y*) coordinates). He proved that only the two phase portraits for ${\mathsf {E}}_0^0{\mathsf {C}}_2{\mathsf {b}}$ can occur and four of the five listed for ${\mathsf {E}}_2^2{\mathsf {C}}_0{\mathsf {c}}$ (if $\delta =2$ and $\rho >0$, then *O* is a sink in his model).

## Discussion

The analysis of the classical two-locus two-allele selection-recombination model is notoriously difficult. As outlined in the Introduction, despite considerable efforts only special cases are well understood, for instance, the models with additive or multiplicative fitnesses. We investigated a simplification of the full two-locus two-allele model (2.3) by assuming that the two loci are independent, i.e., in linkage equilibrium. This simplified model is given by (2.5) and, essentially, goes back to Wright (1942), although special cases had been studied earlier (Haldane 1931; Wright 1935).

The model (2.5) is not only more accessible to mathematical analysis than (2.3), it is also of biological relevance because it has been derived as the weak-selection limit of the full model. Therefore, it provides a good approximation if selection is not too strong and the two loci are unlinked or only weakly linked. Indeed, under the non-degeneracy assumption (${\mathcal {H}}$), a theorem by Nagylaki et al. (1999) demonstrates that in the full model (2.3) and for sufficiently weak selection relative to recombination, every trajectory converges to an equilibrium point, and every such equilibrium point is a perturbation of an equilibrium point of the weak-selection limit (2.5). Throughout this paper, we imposed condition (${\mathcal {H}}$).

Importantly, mean fitness is a strict Lyapunov function for (2.5). Hence, every solution converges to an equilibrium point (Sect. 2). This is not always the case in the full model, neither for (2.3) nor its continuous-time analog. For each the existence of stable limit cycles was demonstrated (Akin 1979, 1982; Hastings 1981b; Hofbauer and Iooss 1984) if selection and recombination are of similar strength. For the weak-selection limit (2.5), Moran (1963) showed that, in addition to the eight possible boundary equilibria, there may exist up to five internal equilibria, and three can be simultaneously stable (Theorem 3.1). For the full model (2.3), the maximum number of equilibria is 15, seven of them being internal equilibria. This is an immediate consequence of a result of Altenberg (2010), who proved the conjecture of Feldman and Karlin (1970) that the maximum number of equilibria in a selection-recombination model with *n* gametes is $2^n-1$.

### Boundary flows, extended boundary flows, and phase portraits

Although we could not fully accomplish our goal of deriving and classifying all possible equilibrium structures and (equivalence classes of) phase portraits of the weak-selection limit (2.5), we identified the extended boundary flows and determined all equilibrium structures for several important types of fitness patterns. These results yield interesting insights into the role of epistasis and dominance in generating equilibrium structures.

For general fitnesses, we identified all possible boundary flows, i.e., flows on the boundary of the state space $[0,1]^2$ (Fig. 1). The four corners correspond to the monomorphic equilibria. The dynamics on the edges correspond to the single-locus dynamics when the other locus is fixed for one or the other allele. There are 42 (topologically) different boundary flows or, more precisely, boundary-flow classes because boundary flows that are obtained by relabeling loci or alleles are identified (Sect. 4, Fig. 2). Of these 42 boundary flows, there are 16 pairs for which a member of a pair is obtained by reversing the flow of the other member. The other 10 boundary-flow classes are self inverse under flow reversal.

These boundary flows are by far not sufficient to describe all possible phase portraits, i.e., the topological structure of the flow on $[0,1]^2$. As an intermediate step, we studied the extended boundary flows (Sect. 4.2). They describe the dynamics in a small neighborhood of the boundary, in particular, the external stability of the edge equilibria. A key ingredient for deriving the possible equilibrium structures and phase portaits on the full state space $[0,1]^2$ from a given extended boundary flow is the boundary index sum $\delta$ (defined below Eq. 4.1). The possible values of $\delta$ are constrained by Lemma 4.1, and Theorem 4.2 shows that for specific boundary flows additional values $\delta$ can be excluded. Thus, we are still left with 190 potentially possible extended boundary flows. The most important tools for drawing conclusions about the internal equilibrium structure are Theorem 4.4, which is a special case of a more general index theorem (Hofbauer 1990), and Corollary 4.5. Overall, we showed existence of 185 extended boundary flows by providing a fitness matrix and a phase portrait generating such an extended boundary flow (SI, Figs. S1–S5, Table S1).

For a given boundary-flow class and a value of $\delta$, there may still exist more than one extended boundary flow yielding this boundary-flow class and this $\delta$. Moreover an extended boundary flow may be compatible with more than one equilibrium structure, and an equilibrium structure may be generated by non-equivalent phase portraits (see Sect. 4.3 and Table S1, as well as the phase portraits in Section S1). Apart from characterizing all possible equilibrium structures or even all possible phase portraits, also more specific problems remain unresolved. For instance, can a sink and a source in the interior coexist? There are also five extended boundary flows, all with $\delta =-1$, whose existence we could not exclude. We conjecture that they do not exist (see Sect. 4.3).

### Permanence

An important notion in modeling biological systems is permanence. This is a generalization of the notion of a protected polymorphism, which is mainly used for one-locus two-allele models in (spatially) structured populations. Loosely speaking, permanence means that no type or species will be lost because its frequency will remain above a certain threshold. For our model (2.5), this implies that a permanent system can exhibit only 14 types of extended boundary flows (Corollary 4.7), all having $\delta =0$. At least one of these extended boundary flows (${\mathsf {E}}^4_4{\mathsf {C}}_0{\mathsf {b}}$) can be generated by permanent flows with one, three, or five internal equilibria (Fig. S1d, panels 4, 5, 6). Thus, the phase portrait is not uniquely determined by the extended boundary flow.

For every given fitness matrix generating one of the 14 boundary-flow classes of Corollary 4.7 and parametrized as in (6.1), a sufficiently strong increase of the epistasis parameter $e_{22}$ yields a permanent flow. This is a consequence of Corollaries 4.7 and A.1, since in the parametrization (6.1), $e_{22}$ is the only parameter in $m_{14}$ that is independent of the given boundary flow.

### Marginal overdominance or underdominance

In Sect. 5, we assumed that each locus exhibits either marginal overdominance at every point (*p*, *q*) or marginal underdominance; see (3.11) and Lewontin and Kojima (1960). This is equivalent to having continuous isoclines (3.4) that map [0, 1] into (0, 1). If there is marginal overdominance at both loci, then ${\mathsf {E}}^4_4{\mathsf {C}}_0{\mathsf {b}}$ is the only possible boundary-flow class and no boundary equilibrium is saturated. Therefore, the extended boundary flow is uniquely determined, $\delta =0$, and the system is permanent. This is compatible with having one, three, or five internal equilibria, of which one, two, or three, respectively, are sinks (Theorem 5.3). We could find phase portraits with one or three internal equilibria, but not with five. However, we could not prove that five internal equilibria cannot be realized. Although Moran’s (1963) example (Figure S1d, panel 6) has boundary-flow class ${\mathsf {E}}^4_4{\mathsf {C}}_0{\mathsf {b}}$ and $\delta =0$, it does not satisfy the assumptions of Theorem 5.3.

A result analogous to Theorem 5.3 holds if both loci exhibit marginal underdominance because then every flow can be obtained by flow reversal. If one locus exhibits marginal overdominance and the other marginal underdominance, then the boundary-flow class is ${\mathsf {E}}^4_2{\mathsf {C}}_0{\mathsf {c}}$, there exists a unique internal equilibrium, which is a saddle, and the two internally stable edge equilibria are linearly stable (Theorem 5.6). Hence, the phase portrait is uniquely determined by the boundary-flow class.

Table S2 lists the extended boundary flows and indicates by which of the special fitness patterns they can be generated.

### Linear isoclines, or additive-by-additive epistasis

A particularly interesting special class arises if linear isoclines are posited (Sect. 6). With the fitness parameterization (6.1) and the assumption (6.3), the dynamics (2.5) simplifies to (6.4). This type of model has been studied independently, in different generality and in different contexts, by Schuster et al. (1981) and by Zhivotovsky and Gavrilets (1992) and Gavrilets (1993); see Sect. 6 for a more detailed appraisal. In population genetics terms, (2.5) has linear isoclines if and only if epistatic interactions are absent or there are only additive-by-additive interactions. The assumption of linear isoclines rules out 16 of the 42 possible boundary flows and greatly reduces the number of possible phase portraits because there can be at most one internal equilibrium. Also the stability of the internal equilibrium, if it exists, is easily determined. Corollary 6.2 lists all possible flows. In particular, the phase portraits of (6.4) are uniquely determined by the boundary flow and by $\delta$. For 12 boundary flows, however, $\delta$ may assume more than one value.

Corollary 6.2 shows also that a stable internal equilibrium cannot exist unless there is at least one internally stable edge equilibrium, i.e., there is overdominance in at least one single-locus boundary system. Analogously, an internal source can occur only if there is underdominance in at least one single-locus boundary system.

As discussed in Sect. 6.1 on the additive model, in the absence of epistasis all trajectories of the full model (2.3) converge to the linkage-equilibrium manifold if the recombination rate is positive. Therefore, the phase portraits derived for (2.5) are representative for the full model. In addition, every possible boundary flow determines the phase portrait uniquely (Corollary 6.3).

Another important special case with linear isoclines is the haploid selection model (6.6). Since there is no dominance, the only possible boundary flows are ${\mathsf {E}}^0_0{\mathsf {C}}_1{\mathsf {s}}$, ${\mathsf {E}}^0_0{\mathsf {C}}_1{\mathsf {a}}$, and ${\mathsf {E}}^0_0{\mathsf {C}}_2{\mathsf {b}}$. As in the additive case, the phase portraits of (6.6) are uniquely determined by the boundary flows: in the first two cases there is no internal equilibrium, in the third case there is a saddle. The weak-selection limit (6.6) of the haploid model captures all possible equilibrium structures and phase portraits of the full haploid model with linkage disequilibrium, at least for continuous time (Bank et al. 2012 and Sect. 6.2).

### Multilinear epistasis

The so-called multilinear model of epistasis was introduced by Hansen and Wagner (2001). It assumes that the effects of gene substitutions due to changes in the genetic background can be described by a linear transformation (Sect. 7). In this model, all types of epistatic interactions can occur. In the two-locus case, these are additive-by-additive, additive-by-dominance, and dominance-by-dominance interactions (cf. Remark 6.1). Interestingly, the multilinearity assumption (7.1) turns out to be equivalent to assuming that the isoclines are in product form; see (7.3). Therefore, they are composed of vertical or horizontal straight lines. In the absence of dominance, the multilinear model and that of Zhivotovsky and Gavrilets (1992) coincide. In this case, both models reduce formally to the haploid model (6.6). Otherwise, they differ. In fact, up to five internal equilibria may occur in the multilinear model. It is also possible to have overdominance on every edge, i.e., in each marginal one-locus system, but no stable internal equilibrium (Table 1, boundary flow ${\mathsf {E}}^4_4{\mathsf {C}}_0{\mathsf {b}}$ with $\delta =4$). All possible equilibrium structures could be identified (Theorem 7.1). Seven of the 16 possible boundary flows determine the phase portrait uniquely.

### Equivalent loci

In Sect. 8, we briefly treat the equilibrium structures generated by symmetric fitness matrices. This is equivalent to assuming that both loci are equivalent, an assumption made in many investigations. Only the nine boundary flows of Fig. 2 ending with an ${\mathsf {s}}$ or ${\mathsf {b}}$ can occur. The symmetry properties of this model greatly simplify its analysis, so that for every possible boundary flow the possible values of $\delta$ and the possible number of internal equilibria can be determined (Theorem 8.1). Moran’s (1963) example of a flow with three stable internal equilibria is symmetric. Thus, the assumption of symmetry reduces the complexity of the two-locus model only in certain aspects.

### The symmetric viability model

As indicated in the Introduction and in Sect. 9, the symmetric viability model may be one of the best studied dynamical systems in population genetics. Although it depends on only four parameters, its complexity seems to preclude a comprehensive mathematical analysis. Even the subclass arising from models of stabilizing selection on a quantitative trait toward an intermediate optimum is well understood only for special cases, such as quadratic or Gaussian stabilizing selection (Bürger 2000, Chap. 6.2; Willensdorfer and Bürger 2003).

The weak-selection limit, Eq.  (9.3) in our parameterization (9.1) and in the transformed coordinates, is still sufficiently complex to admit a wide variety of equilibrium structures and phase portraits. Nevertheless, it is simple enough to admit the identification of all possible equilibrium structures (Theorem 9.5 and Table 2). This requires knowledge of the boundary-flow class (by definition, the types ending with ${\mathsf {b}}$ or ${\mathsf {c}}$ occur), the boundary index sum, and the sign of two simple compound parameters ($\rho =r_1r_2-m^2$ and $r_1+r_2$).

Theorem 9.5 shows that the maximum number of internal equilibria of (9.3) is five and the maximum number of stable internal equilibria is two. This is different in the full two-locus symmetric viability model with linkage disequilibrium. Then the maximum number of internal equilibria is seven (Feldman and Karlin 1970), and four can be simultaneously stable (Hastings 1985). Interestingly, the same maximum numbers of internal and of stable internal equilibria occur in the special case arising from Gaussian stabilizing selection on a quantitative trait (Willensdorfer and Bürger 2003). In the full two-locus model, three of the seven internal equilibria are so called symmetric equilibria whose gamete frequencies satisfy $x_1=x_4$ and $x_2=x_3$. In the weak-selection limit, these symmetry conditions collapse to $p=\frac{1}{2}$ and $q=\frac{1}{2}$ or, in the (*x*, *y*) coordinates of Sect. 9, to $x=y=0$. Thus, in the weak-selection limit, the manifold defined by $x_1=x_4$ and $x_2=x_3$ collapses to the single point $(x,y)=(0,0)$. The four unsymmetric equilibria determined by Feldman and Karlin (1970) for the full model correspond to the four unsymmetric internal equilibria determined by Theorem 9.1.

### Multiplicative fitnesses

The reader may have noticed that we did not consider multiplicative fitnesses, although the multiplicative viability model has received great attention in the literature. This model has always been treated in discrete time because then multiplicate fitnesses have an immediate biological meaning. In contrast to the additive viability model, it has the property that the linkage-equilibrium manifold ($D=0$) is invariant under the map (2.3). Also in contrast to the additive model, solutions do not necessarily approach linkage equilibrium, and stable equilibria with $D\ne 0$ may exist. By performing the weak-selection limit, multiplicative fitnesses become additive because $(1+\epsilon a)(1+\epsilon b) \approx 1 + \epsilon (a+b)$. Thus, the weak-selection limits of the additive and the multiplicative model coincide.

### Inferring stable two-locus polymorphisms

An interesting, old question is whether the maintenance of a stable two-locus polymorphism requires some form of overdominance at the individual loci. Kojima (1959) showed for independent loci, i.e., our model (2.5), that marginal overdominance (3.11) on both loci at the equilibrium point is necessary for the maintenance of a stable two-locus polymorphism. However, this condition is not sufficient (Corollary 3.3). For the full two-locus model, Hastings (1982) showed that for a small range of recombination rates stable internal equilibria (sinks) may display marginal underdominance. Theorem 5.3 and Fig. S1d (panel 5) show that unstable internal equilibria may exist even if both loci display marginal overdominance on the whole state space.

A simpler and more general question is whether inferences about existence and stability of internal equilibria can be drawn from knowledge of the boundary flow. In general, the answer is negative. Without further restrictions on the fitness scheme, (2.2) or (6.1), there is no boundary flow that determines the phase portrait uniquely. The boundary-flow class ${\mathsf {E}}^0_0{\mathsf {C}}_2{\mathsf {b}}$ is the only one for which an internal equilibrium exists always. All other boundary-flow classes can have $\delta =1$ and all can be realized without an internal equilibrium (Figs. S2a – S2k). It is even possible to maintain an internal sink if no edge equilibrium exists, i.e., if all four marginal one-locus systems display intermediate dominance. Indeed, all three boundary-flow classes ${\mathsf {E}}^0_0$ admit an internal sink (Fig. S2a, panels 2 and 4; Fig. S3a, panel 2), but none of our special fitness patterns does so.

In the model with linear isoclines, a stable internal equilibrium can occur only if there is overdominance at least at two edges (boundary flows ${\mathsf {E}}^2_2{\mathsf {C}}_0{\mathsf {c}}$, ${\mathsf {E}}^2_2{\mathsf {C}}_0{\mathsf {s}}$, ${\mathsf {E}}^3_3{\mathsf {C}}_0{\mathsf {e}}$). Overdominance at all four edges (${\mathsf {E}}^4_4{\mathsf {C}}_0{\mathsf {b}}$) is sufficient for the existence of an internal sink (Corollary 6.2). For multilinear epistasis, which admits not only additive-by-additive but also additive-by-dominance and dominance-by-dominance interactions (though not in their most general form), an internal sink can exist only if there are four edge equilibria. However, for each of the six resulting boundary flows, there may exist either an internal sink or an internal source (Theorem 7.1). For the symmetric viability model, at least two edge equilibria are necessary for the existence of an internal sink. However, for each of the five resulting boundary flows, there may exist either an internal sink or an internal source (Theorem 9.5). Nevertheless, for the models with linear isoclines (i.e., only additive-by-additive epistasis) and with multilinear epistasis, many boundary-flow classes determine the equilibrium structure uniquely (Corollary 6.2 and Theorem 7.1).

Our treatment of special fitness patterns is not exhaustive. For instance, Hastings and Hom (1990) determined the equilibrium structure of a model with stabilizing selection toward an optimum with arbitrary position by assuming absence of linkage disequilibrium. More recently, Feldman and Puniyani (2006) examined an extension of the Lewontin–Kojima version of the full two-locus symmetric viability model, in which substitutions at locus *A* do not depend on the background alleles at locus *B*, but substitutions at locus *B* depend on the background locus *A*. The boundary flow classes that arise in this framework are ${\mathsf {E}}^2_2{\mathsf {C}}_0{\mathsf {e}},\ {\mathsf {E}}^2_1{\mathsf {C}}_0{\mathsf {e}},\ {\mathsf {E}}^2_1{\mathsf {C}}_2{\mathsf {e}},\ {\mathsf {E}}^4_4{\mathsf {C}}_0{\mathsf {b}},\ {\mathsf {E}}^4_3{\mathsf {C}}_0{\mathsf {e}}$, and ${\mathsf {E}}^4_2{\mathsf {C}}_0{\mathsf {c}}.$

### Electronic supplementary material

Below is the link to the electronic supplementary material.

Supplementary material 1 (pdf 9323 KB)

## Appendix

### The external eigenvalues

First we evaluate the Jacobian for the system (2.5) at each of the edges and obtain an interesting connection between the external eigenvalues and the marginal fitnesses (3.5) and (3.6):

A.1a $$\begin{aligned} \lambda _{34}&=m_{A_1A_2}-m_{A_2A_2}, \end{aligned}$$

A.1b $$\begin{aligned} \lambda _{12}&=m_{A_1A_2}-m_{A_1A_1}, \end{aligned}$$

A.1c $$\begin{aligned} \lambda _{24}&=m_{B_1B_2}-m_{B_2B_2}, \end{aligned}$$

A.1d $$\begin{aligned} \lambda _{13}&=m_{B_1B_2}-m_{B_1B_1}. \end{aligned}$$

Here, $\lambda _{ij}$ is the external eigenvalue at the boundary that contains $E_{ij}$. On each edge of the unit square one allele is missing, and the marginal fitnesses of the one-locus genotypes that contain the present allele define the external eigenvalue.

The eigenvalues $\lambda _{ij}$ evaluated at the equilibria $E_{ij}$ are

A.2a $$\begin{aligned} \hat{\lambda }_{34}=&\frac{1}{\xi _{34}^2}\left[ (m_{13}-m_{33})(m_{34}-m_{44})^2+2(m_{14}-m_{34})(m_{34}-m_{44})(m_{34}-m_{33})\right. \nonumber \\&\left. +\,(m_{24}-m_{44})(m_{34}-m_{33})^2\right] , \end{aligned}$$

A.2b $$\begin{aligned} \hat{\lambda }_{12}=&\frac{1}{\xi _{12}^2}\left[ (m_{13}-m_{11})(m_{12}-m_{22})^2+2(m_{14}-m_{12})(m_{12}-m_{22})(m_{12}-m_{11})\right. \nonumber \\&\left. +\,(m_{24}-m_{22})(m_{12}-m_{11})^2\right] , \end{aligned}$$

A.2c $$\begin{aligned} \hat{\lambda }_{24}=&\frac{1}{\xi _{24}^2}\left[ (m_{12}-m_{22})(m_{24}-m_{44})^2+2(m_{14}-m_{24})(m_{24}-m_{44})(m_{24}-m_{22})\right. \nonumber \\&\left. +\,(m_{34}-m_{44})(m_{24}-m_{22})^2\right] , \end{aligned}$$

A.2d $$\begin{aligned} \hat{\lambda }_{13}=&\frac{1}{\xi _{13}^2}\left[ (m_{12}-m_{11})(m_{13}-m_{33})^2+2(m_{14}-m_{13})(m_{13}-m_{33})(m_{13}-m_{11})\right. \nonumber \\&\left. +\,(m_{34}-m_{33})(m_{13}-m_{11})^2\right] , \end{aligned}$$

where $\xi _{ij}=2m_{ij}-m_{ii}-m_{jj}$ for $(i,j)\in \{(1,2),(1,3),(2,4),(3,4)\}$. The sign of $\hat{\lambda }_{ij}$ determines whether $E_{ij}$ is (strictly) saturated or not. Therefore, we omit the leading positive factor in many computations in the main text as well as in the appendix.

#### Corollary A.1

For sufficiently large $|m_{14}|$ the external eigenvalue $\hat{\lambda }_{ij}$ has the same sign as $m_{14}$.

#### Proof

This holds because $m_{14}$ shows up in every $\hat{\lambda }_{ij}$ at the same position and is independent of the given boundary flow and the two other factors multiplied with it, which have always the same sign if the corresponding edge equilibrium is admissible. $\square$

### Proof of Theorem 4.2

(a) Since $\delta =-2$ can occur only for ${\mathsf {E}}^4_2{\mathsf {C}}_0{\mathsf {c}}$, it is sufficient to exclude this case.

The boundary flow ${\mathsf {E}}^4_2{\mathsf {C}}_0{\mathsf {c}}$ has four equilibria on the boundary edges. The upper ($E_{12}$) and the lower ($E_{34}$) one are internally stable, while the two others ($E_{24}, E_{13}$) are internally unstable. We assume that $E_{34}$ and $E_{12}$ are not saturated, whereas the other two are. Then $\delta =-2$. Now, we derive the conditions that the viability parameters have to satisfy. $\hat{\lambda }_{34}$ and $\hat{\lambda }_{12}$, given by (A.2), need to be positive, i.e.,

A.3 $$\begin{aligned}&(m_{13}-m_{33})(m_{34}-m_{44})^2+2(m_{14}-m_{34})(m_{34}-m_{44})(m_{34}-m_{33}) \nonumber \\&\quad +(m_{24}-m_{44})(m_{34}-m_{33})^2>0, \end{aligned}$$

A.4 $$\begin{aligned}&(m_{13}-m_{11})(m_{12}-m_{22})^2+2(m_{14}-m_{12})(m_{12}-m_{22})(m_{12}-m_{11}) \nonumber \\&\quad +(m_{24}-m_{22})(m_{12}-m_{11})^2>0, \end{aligned}$$

whereas $\hat{\lambda }_{24}$ and $\hat{\lambda }_{13}$ need to be negative, i.e.,

A.5 $$\begin{aligned}&(m_{12}-m_{22})(m_{24}-m_{44})^2+2(m_{14}-m_{24})(m_{24}-m_{44})(m_{24}-m_{22}) \nonumber \\&\quad +(m_{34}-m_{44})(m_{24}-m_{22})^2<0, \end{aligned}$$

A.6 $$\begin{aligned}&(m_{12}-m_{11})(m_{13}-m_{33})^2+2(m_{14}-m_{13})(m_{13}-m_{33})(m_{13}-m_{11}) \nonumber \\&\quad +(m_{34}-m_{33})(m_{13}-m_{11})^2<0. \end{aligned}$$

For $\hat{\lambda }_{34}$ and $\hat{\lambda }_{12}$, the first and the third term are negative because there is underdominance at these edges. Therefore, the second term must be positive, otherwise the eigenvalues would be negative for all choices of parameters. For $\hat{\lambda }_{24}$ and $\hat{\lambda }_{13}$, the signs of the first and third terms are positive and this implies a negative second term. Therefore, we obtain the following inequalities:

A.7 $$\begin{aligned} m_{12},m_{34}<m_{14}<m_{24},m_{13}. \end{aligned}$$

From the boundary flow of ${\mathsf {E}}^4_2{\mathsf {C}}_0{\mathsf {c}}$ one can read off the relations

A.8 $$\begin{aligned} m_{24}<m_{12},m_{34}\; \text {and}\; m_{13}<m_{12},m_{34}, \end{aligned}$$

which yields the desired contradiction.

(b)–(d) These proofs are similar to the one above and left to the reader.

### Proof of Theorem 7.1

First we investigate when $\varphi _i(x)=0$ has zero, one, or two solutions in (0, 1). From (7.3c) we find

A.9a $$\begin{aligned} \varphi _i(0)&= (a_1+d_1)(a_2+d_2), \end{aligned}$$

A.9b $$\begin{aligned} \varphi _i(1)&= (a_1+d_1)(a_2+d_2) + 2a_i e_{22}, \end{aligned}$$

A.9c $$\begin{aligned} \varphi _i'(x_i)&=0 \quad \text {if and only if}\quad x_i = \frac{a_i+d_i}{2d_i}, \end{aligned}$$

A.9d $$\begin{aligned} \varphi _i\left( \frac{a_i+d_i}{2d_i}\right)&= (a_1+d_1)(a_2+d_2) +\frac{(a_i+d_i)^2e_{22}}{2d_i}, \end{aligned}$$

where we recall from (7.4) that ${\tilde{p}}=\frac{a_1+d_1}{2d_1}$ and ${\tilde{q}}=\frac{a_2+d_2}{2d_2}$. As a simple consequence we obtain:

#### Lemma A.2

The number of zeros in (0, 1) of the isocline $\varphi _i$ is

- one if and only if
  A.10 $$\begin{aligned} {\text {sgn}}\varphi _i(1) = -{\text {sgn}}\varphi _i(0); \end{aligned}$$
- two if and only if the following three conditions hold:
  A.11a $$\begin{aligned} {\text {sgn}}\varphi _i(1) = {\text {sgn}}\varphi _i(0), \end{aligned}$$
  A.11b $$\begin{aligned} 0<\frac{a_i+d_i}{2d_i}<1, \text { i.e., } \left| d_i\right| >a_i, \end{aligned}$$
  A.11c $$\begin{aligned} {\text {sgn}}\varphi _i\left( \frac{a_i+d_i}{2d_i} \right) = -{\text {sgn}}\varphi _i(0); \end{aligned}$$
- zero if neither (A.10) nor (A.11) are fulfilled.

Obviously, (b) requires $0<{\tilde{q}}<1$ if $i=1$, and $0<{\tilde{p}}<1$ if $i=2$.

Lemma A.2 has a number of important consequences:

#### Remark A.3

(i) Because $\dot{p}=0$ if and only if $p={\tilde{p}}$ or $\varphi _2(q)=0$, the flows on the opposite edges $q=0$ and $q=1$ are equivalent if and only if $\varphi _2(q)=0$ has zero or two solutions in (0, 1) which, in turn, is equivalent to $\varphi _2(0)$ and $\varphi _2(1)$ having the same sign. Otherwise, one is the flow reversal of the other. An analogous statement holds for the flows on the opposite edges $p=0$ and $p=1$.
(ii) If $\varphi _2(q)=0$ has two solutions, $q_1$ and $q_2$, then ${\text {sgn}}\dot{p}(p,q) = -{\text {sgn}}\dot{p}(p,0)$ $= -{\text {sgn}}\dot{p}(p,1)$ for every $q\in (q_1,q_2)$. This applies in particular to ${\tilde{q}}$. Therefore, the edge equilibrium $E_{12}=(1,{\tilde{q}})$ is externally stable if and only if $\dot{p}< 0$ on the edges $q=0$ and $q=1$ for *p* close to 1. An analogous statement holds if $\varphi _1(p)=0$ has two solutions.
(iii) If $\varphi _2(q)=0$ has no solutions in (0, 1), then $\dot{p}$ does not change sign in small neighborhoods of $p=0$ and of $p=1$. Therefore, $E_{12}$ is externally unstable if and only if $C_1$ (or $C_2$) is unstable on the edge $q=1$ ($q=0$).
(iv) If there is (precisely) one pair of edge equilibria, the number of intersection points of $\varphi _1(p)=0$ and $\varphi _2(q)=0$ in $(0,1)^2$ can be 0, 1, or 2. Since in this case the equilibrium $({\tilde{p}}, {\tilde{q}})$ does not exist, the number of internal equilibria can be only 0, 1, or 2.
(v) If there are four edge equilibria, the number of intersection points of $\varphi _1(p)=0$ and $\varphi _2(q)=0$ in $(0,1)^2$ can be 0, 1, 2, 4, and the number of internal equilibria can be 1, 2, 3, or 5.

In the main text, we already showed that edge equilibria occur only in pairs at opposite edges. This excludes all boundary flows of type ${\mathsf {E}}^1_m{\mathsf {C}}_k$ and ${\mathsf {E}}^3_m{\mathsf {C}}_k$, but also some others (see below).

**Boundary flows with no edge equilibria** Because opposite edge equilibria occur pairwise, they occur if and only if dominance is intermediate at both loci, i.e., if and only if $-a_i<d_i<a_i$ for $i=1$ and $i=2$. The possible boundary flows are ${\mathsf {E}}^0_0{\mathsf {C}}_1{\mathsf {s}}$, ${\mathsf {E}}^0_0{\mathsf {C}}_1{\mathsf {a}}$, and ${\mathsf {E}}^0_0{\mathsf {C}}_2{\mathsf {b}}$. By Lemma A.2b, there is at most one solution of $\varphi _i(x)=0$ in (0, 1), whence the number of internal equilibria is zero or one. Because one internal equilibrium can occur only if $\varphi _1(p)=0$ and $\varphi _2(q)=0$ intersect, which implies that the flows at opposite edges have different direction, the boundary flow ${\mathsf {E}}^0_0{\mathsf {C}}_2{\mathsf {b}}$ is the only in this class with an internal equilibrium. Because $\delta =2$, it is a saddle. This proves statement (a) of Theorem 7.1 for the top three boundary flows in Table 1.

**Boundary flows with one pair of edge equilibria** Without loss of generality we assume that the pair $E_{12}=(1,{\tilde{q}})$ and $E_{34}=(0,{\tilde{q}})$ exists, and $-a_1<d_1<a_1$. Although not necessary, for the ease of the argument we compute the eigenvalues at the edge equilibria. Recall from (3.3) and (A.2) that the internal eigenvalue at the edge equilibrium $E_{ij}$ is denoted by $\mu _{ij}$ and the external by $\hat{\lambda }_{ij}$. We obtain  Therefore, $E_{34}$ and $E_{12}$ have the same internal stability if and only if $\varphi _1(0)$ and $\varphi _1(1)$ have the same sign. They have different external stability because there is intermediate dominance at the other locus.

As a consequence, since there is only one pair of edge equilibria, their total contribution to the boundary index sum $\delta$ is either $-1$ or 1. These considerations restrict the possible boundary flows and boundary index sums $\delta$ to (cf. Fig. 2, Lemma 4.1, Theorem 4.2): ${\mathsf {E}}^2_2{\mathsf {C}}_0{\mathsf {c}}$ and ${\mathsf {E}}^2_2{\mathsf {C}}_0{\mathsf {e}}$, their flow reversals ${\mathsf {E}}^2_0{\mathsf {C}}_2{\mathsf {c}}$ and ${\mathsf {E}}^2_0{\mathsf {C}}_2{\mathsf {e}}$, ${\mathsf {E}}^2_1{\mathsf {C}}_0{\mathsf {e}}$ (all with $\delta =1$), ${\mathsf {E}}^2_1{\mathsf {C}}_1{\mathsf {a}}'''$ with $\delta =0$ or $\delta =2$, and ${\mathsf {E}}^2_1{\mathsf {C}}_2{\mathsf {e}}$ with $\delta =1$ or $\delta =3$.

Because the internal equilibria can result only from intersection points of $\varphi _1(p)=0$ and $\varphi _2(q)=0$, there are two internal equilibria if and only if

A.13a $$\begin{aligned} {\text {sgn}}\varphi _2({\tilde{q}}) = -{\text {sgn}}\varphi _2(1) = -{\text {sgn}}\varphi _2(0) \end{aligned}$$

and

A.13b $$\begin{aligned} {\text {sgn}}\varphi _1(1) = -{\text {sgn}}\varphi _1(0)\,; \end{aligned}$$

cf. Remark A.3(ii), (iv). (A.13) can be satisfied only if the opposite edge equilibria have different internal stability and if the flows on the other pair of edges have different direction. As a consequence of (A.13), the flows ${\mathsf {E}}^2_2{\mathsf {C}}_0{\mathsf {c}}$, ${\mathsf {E}}^2_2{\mathsf {C}}_0{\mathsf {e}}$, ${\mathsf {E}}^2_0{\mathsf {C}}_2{\mathsf {c}}$, ${\mathsf {E}}^2_0{\mathsf {C}}_2{\mathsf {e}}$, and ${\mathsf {E}}^2_1{\mathsf {C}}_0{\mathsf {e}}$, which all have $\delta =1$, cannot have an internal equilibrium. Thus, in all these cases the phase portrait is uniquely determined by the boundary flow and statement (a) of Theorem 7.1 is proved for these five boundary flows.

The boundary flow ${\mathsf {E}}^2_1{\mathsf {C}}_1{\mathsf {a}}'''$ with $\delta =0$ can be excluded, as follows. Concordant with Fig. 2, we assume that $E_{12}$ is internally unstable and $E_{34}$ is internally stable. Then $\delta =0$ requires that $E_{12}$ is externally stable and $E_{34}$ is externally unstable. Therefore, (A.12b) implies ${\text {sgn}}\varphi _1(0)=-{\text {sgn}}\varphi _1(1)$. To obtain ${\mathsf {E}}^2_1{\mathsf {C}}_1{\mathsf {a}}'''$, we need $\dot{p}>0$ if $q=0$, and $\dot{p}<0$ if $q=1$. These assumptions are equivalent to the following conditions: $-a_1<d_1<a_1$, and $d_2>a_2>0$ and ${\text {sgn}}\varphi _2(1)=-{\text {sgn}}\varphi _2(0)$. Therefore, we have $\varphi _1(0)=\varphi _2(0)=(a_1+d_1)(a_2+d_2)>0$ and external instability of $E_{34}$ requires $\hat{\lambda }_{34}>0$. Since we also need $\varphi _i(1)=(a_1+d_1)(a_2+d_2)+2a_ie_{22}<0$ for $i=1$ and $i=2$, we obtain a contradiction because, by (A.12a), $e_{22}$ had to satisfy

A.14 $$\begin{aligned} -\frac{2d_2(a_1+d_1)}{a_2+d_2}< e_{22} <-\frac{(a_1+d_1)(a_2+d_2)}{2a_2}. \end{aligned}$$

This is impossible because the expression on the left exceeds that on the right. Therefore, only $\delta =2$ is possible and the internal equilibrium is a saddle. This proves statement (a) of Theorem 7.1 for the boundary flow ${\mathsf {E}}^2_1{\mathsf {C}}_1{\mathsf {a}}'''$.

Finally, we treat the only remaining boundary flow, ${\mathsf {E}}^2_1{\mathsf {C}}_2{\mathsf {e}}$. This has either zero or two internal equilibria. Obviously, if $\delta =3$, there must be two internal saddles. If $\delta =1$, two internal equilibria can be excluded because this would require that the internally unstable edge equilibrium be also externally unstable, which is impossible by the argument in (ii) above. This completes the proof of statement (a) of Theorem 7.1 for all boundary flow with two edge equilibria.

**Boundary flows with two pairs of edge equilibria** Since there are two pairs of opposite edge equilibria, the central equilibrium $({\tilde{p}}, {\tilde{q}})$ exists, and opposite equilibria have the same external stability by (A.12) and its analog for the other locus. Therefore, the total contribution of the edge equilibria to the boundary index sum $\delta$ is $-4$, $-2$, 0, 2, or 4. These considerations restrict the possible boundary flows and boundary index sums $\delta$ to (Fig. 2, Lemma 4.1, Theorem 4.2): ${\mathsf {E}}^4_4{\mathsf {C}}_0{\mathsf {b}}$ with $\delta =0$ or $\delta =2$ or $\delta =4$; ${\mathsf {E}}^4_3{\mathsf {C}}_0{\mathsf {e}}$ with $\delta =0$ or $\delta =2$; their flow reversals ${\mathsf {E}}^4_0{\mathsf {C}}_4{\mathsf {b}}$ and ${\mathsf {E}}^4_0{\mathsf {C}}_4{\mathsf {b}}$ with the same values of $\delta$; ${\mathsf {E}}^4_2{\mathsf {C}}_0{\mathsf {c}}$ with $\delta =0$ or $\delta =2$; and ${\mathsf {E}}^4_2{\mathsf {C}}_1{\mathsf {s}}$ with $\delta =1$.

From Lemma A.2(b) we infer that $\varphi _1(p)$ and $\varphi _2(q)$ can have four intersection points in $(0,1)^2$ only if at each locus their is either overdominance at both edges, or underdominance at both edges (by Remark A.3(i), the flows on opposite edges are equivalent if there are four intersection points). If there is overdominance at both loci ($d_i>a_i$ for $i=1,2$), a simple calculation shows that there are four intersection points if and only if

A.15 $$\begin{aligned} -\frac{(a_1+d_1)(a_2+d_2)}{2a_i}< e_{22} < -\frac{2d_i(a_1+d_1)(a_2+d_2)}{(a_i+d_i)^2} \;\text {for } i =1,2. \end{aligned}$$

It is easily shown that this condition on $e_{22}$ can be satisfied for every choice of $\left| d_i\right| > a_i$.

If there is underdominance at both loci ($d_i<-a_i$ for $i=1,2$), the condition for four admissible intersection points is again (A.15). If there is overdominance at one locus and underdominance at the other, then $\varphi _i(0)<0$ for $i=1,2$. This leads to a contradiction with the requirement $\varphi _i\left( \frac{a_i+d_i}{2d_i}\right) >0$ (A.11c), which is equivalent to $e_{22}\frac{(a_i+d_i)^2}{2d_i}>-(a_1+d_1)(a_2+d_2)$, because the left-hand side assumes different signs for $i=1$ and $i=2$, whereas the right-hand side is positive.

Therefore, five internal equilibria can occur only for the boundary flow ${\mathsf {E}}^4_4{\mathsf {C}}_0{\mathsf {b}}$ and its flow reversal ${\mathsf {E}}^4_0{\mathsf {C}}_4{\mathsf {b}}$. For all other equilibrium structures with four edge equilibria, the number of internal equilibria is at most three by Remark A.3(v).

The eigenvalues of the central equilibrium $({\tilde{p}}, {\tilde{q}})$ are easily calculated and can be written as

A.16 $$\begin{aligned} \nu _1 = \frac{a_1-d_1}{2d_1(a_2+d_2)}\,\varphi _2({\tilde{q}}) \;\text { and }\; \nu _2 = \frac{a_2-d_2}{2d_2(a_1+d_1)}\,\varphi _1({\tilde{p}}). \end{aligned}$$

The external eigenvalues of the edge equilibria are obtained from (A.12) and its analogon for the second locus.

For the boundary flow ${\mathsf {E}}^4_4{\mathsf {C}}_0{\mathsf {b}}$, the number of intersection points of $\varphi _1(p)=0$ and $\varphi _2(q)=0$ can be only zero or four because the flows on opposite edges are equivalent. Therefore, the number of internal equilibria can be only one or five. If there are five, then $({\tilde{p}},{\tilde{q}})$ is a source (by Lemma A.2b and because $\varphi _i(0)>0$) and all edge equilibria are externally stable. Therefore, $\delta =4$ and the other internal equilibria are saddles. If $({\tilde{p}},{\tilde{q}})$ is the only internal equilibrium, it is a saddle if $\delta =2$, and it is globally asymptotically stable if $\delta =0$. All these cases can be realized (Table S3). This proves statement (a) of Theorem 7.1 for ${\mathsf {E}}^4_4{\mathsf {C}}_0{\mathsf {b}}$ and its reversed boundary flow ${\mathsf {E}}^4_0{\mathsf {C}}_4{\mathsf {b}}$.

We treat ${\mathsf {E}}^4_3{\mathsf {C}}_0{\mathsf {e}}$. We have already shown that the boundary index sum is either $\delta =0$ or $\delta =2$. Following Fig. 2, we assume without loss of generality that $E_{34}$ is the internally unstable equilibrium. Remark A.3(i) implies that $\varphi _1(p)=0$ has precisely one solution in (0, 1) and $\varphi _2(q)=0$ has zero or two solutions in (0, 1). Therefore, the number of intersection points of $\varphi _1(p)$ and $\varphi _2(q)$ is zero or two and the number of internal equilibria is one or three. If there are no intersection points, the central equilibrium is a sink if $\delta =0$ (because then, by A.3(iii), all four edge equilibria are externally unstable), and it is a saddle if $\delta =2$. If there are two intersection points and $\delta =2$, the central equilibrium is a source (because all edge equilibria are externally stable) and the other two internal equilibria are saddles. With two intersection points, the case $\delta =0$ can be excluded as follows: As in Fig. 2, assume that $E_{34}$ is the internally unstable equilibrium. Because the other three edge equilibria are internally stable, we have the conditions $d_2<-a_2<0$, $d_1>a_1>0$, $(a_1+d_1)(a_2+d_2)+2a_2e_{22}<0$, and $(a_1+d_1)(a_2+d_2)+2a_1e_{22}>0$ [see (A.9), (A.10) and statement (i) above]. By (A.11c) or (A.12a), for two intersection points also $(a_1+d_1)+e_{22}{\tilde{q}}<0$ needs to be satisfied. This easily leads to a contradiction and proves statement (a) for ${\mathsf {E}}^4_3{\mathsf {C}}_0{\mathsf {e}}$ and its reversed flow ${\mathsf {E}}^4_1{\mathsf {C}}_2{\mathsf {e}}$.

For ${\mathsf {E}}^4_2{\mathsf {C}}_0{\mathsf {c}}$ flows on the opposite edges are equivalent and therefore, the number of intersection points of $\varphi _1(p)=0$ and $\varphi _2(q)=0$ can only be zero or four. The latter was excluded above. Therefore, the internal equilibrium is a sink or a source if $\delta =0$, and it is a saddle if $\delta =2$. Since these cases can be realized, statement (a) is proved for ${\mathsf {E}}^4_2{\mathsf {C}}_0{\mathsf {c}}$.

Finally, for ${\mathsf {E}}^4_2{\mathsf {C}}_1{\mathsf {s}}$ (which can only have $\delta =1$) there exists the central equilibrium $({\tilde{p}}, {\tilde{q}})$ and precisely one additional equilibrium because (A.10) holds for $i=1$ and $i=2$. The central equilibrium can be a sink or a source, the other equilibrium is a saddle (this follows easily from the eigenvalues at $({\tilde{p}}, {\tilde{q}})$ and the fact that opposite edges have different flow direction). This proves statement (a) of Theorem 7.1 for ${\mathsf {E}}^4_2{\mathsf {C}}_1{\mathsf {s}}$.

Since all other cases have been excluded, we have proved that all possible equilibrium structures are listed in Table 1. Statement (b) of Theorem 7.1 follows easily from Table 1. Parameter combinations that yield all possible equilibrium structures are given in Table S3.

### Proof of Theorem 8.1

The statement about the possible boundary flows and the values of $\delta$ was proved below Eq. (8.1). The existence of equilibrium structures listed in Theorem 8.1 is shown in Section S1 of the SI: Figures S2d (panels 1,2,3), S4a (panels 1,2), and S4b (panels 5,6) show (a); Figures S1d (panels 4,5,6) and S4c (panels 1,3,5) show (b); Figures S1a (panels 5,6), S2a (panels 1,2), S2k (panels 2,3), S3a (panels 1,2), S3b (panels 2,3,4), and S3h (panels 4,5) show (c). What remains to be proved is that four or five internal equilibria cannot occur for the boundary flows listed in statement (c).

As already stated in the main text, the quintic polynomial $f(g(p))-p$ (3.7), which yields all internal equilibria, factorizes into a quadratic and a cubic polynomial. Since we want to apply Descartes’ rule of signs (which states that the number of positive roots of a polynomial is either equal to the number of sign changes between consecutive nonzero coefficients which are ordered by ascending variable exponent, or is less than it by an even number), we transform theses polynomials using $u=p/(1-p)$. Then the zeros $p\in (0,1)$ correspond to the zeros $u\in (0,\infty )$.

For the cubic polynomial $r(u)=\sum _{i=0}^3 r_iu^i$ that yields the symmetric equilibria (i.e., those satisfying $p=q$), we obtain  and for quadratic polynomial $s(u)=\sum _{i=0}^2 s_iu^i$ that yields the pair of asymmetric equilibria, we obtain

A.18a $$\begin{aligned} s_0&= -4d^2-d(e_{12}+2e_{22})-a(e_{12}-2e_{22}), \end{aligned}$$

A.18b $$\begin{aligned} s_1&= -8d^2-d(e_{11}-4e_{12}+12e_{22})-a(e_{11}-2e_{12})+2e_{22}(e_{12}-2e_{22}), \end{aligned}$$

A.18c $$\begin{aligned} s_2&=-4d^2+d(e_{11}-3e_{12}-2e_{22})-a(e_{11}-3e_{12}+2e_{22})+e_{12}(e_{12}-2e_{22}). \end{aligned}$$

From symmetry it follows immediately that if only one zero of *s*(*u*) is positive, the resulting equilibrium cannot be admissible. Therefore, to obtain a pair of admissible equilibria from $s(u)=0$, the coefficients $s_0$ and $s_2$ must have the same sign and the coefficient $s_1$ must have the opposite sign.

We start with the boundary flows ${\mathsf {E}}^4_4{\mathsf {C}}_0{\mathsf {b}}$ (with $\delta =2$), ${\mathsf {E}}^4_0{\mathsf {C}}_4{\mathsf {b}}$ (with $\delta =2$), and ${\mathsf {E}}^4_2{\mathsf {C}}_1{\mathsf {s}}$ (with $\delta =1$), for which the proof is simple and based on the observation that the external eigenvalues $\lambda _{ij}$ at the edge equilibria $E_{ij}$ satisfy $\lambda _{12}=\lambda _{13}=-s_2r_3$ and $\lambda _{24}=\lambda _{34}=s_0r_0$. We prove the claim for ${\mathsf {E}}^4_4{\mathsf {C}}_0{\mathsf {b}}$ with $\delta =2$; then the case ${\mathsf {E}}^4_0{\mathsf {C}}_4{\mathsf {b}}$ follows from flow reversal, and the case ${\mathsf {E}}^4_2{\mathsf {C}}_1{\mathsf {s}}$ is analogous. Because $\delta =2$, ${\text {sgn}}(\hat{\lambda }_{12})=-{\text {sgn}}({\hat{\lambda }_{34}})$ has to hold, whence ${\text {sgn}}(s_2r_3)={\text {sgn}}(s_0r_0)$ follows. In addition, admissibility and internal stability of the equilibria $E_{12}$ and $E_{24}$ implies $r_0<0$ and $r_3>0$. Hence, $s_2$ and $s_0$ have opposite signs. This rules out equilibria off the diagonal $p=q$, whence the maximum number of internal equilibria is three.

For the boundary flows ${\mathsf {E}}^0_0{\mathsf {C}}_1{\mathsf {s}}$, ${\mathsf {E}}^0_0{\mathsf {C}}_2{\mathsf {b}}$, ${\mathsf {E}}^2_2{\mathsf {C}}_0{\mathsf {s}}$, and ${\mathsf {E}}^2_0{\mathsf {C}}_2{\mathsf {s}}$, the proofs are more technical and different proofs are needed for each case.

First, we consider the boundary flow ${\mathsf {E}}^0_0{\mathsf {C}}_2{\mathsf {b}}$. Without loss of generality, we can assume $a+d=-1$ and $a<0$ in (6.1) with (8.1). Then the boundary flow ${\mathsf {E}}^0_0{\mathsf {C}}_2{\mathsf {b}}$ is realized if and only if the inequalities $d>-\frac{1}{2}$ and $1<e_{12}<e_{11}-1-2d$ hold. The proof is by contradiction. Therefore, assume that five internal equilibria exist. Then the signs of the coefficients of *r*(*u*) have to change three times, where we already know that $r_0=1>0$. The conditions $r_1<0$ and $r_2>0$ result in the additional inequalities $d<-\frac{3}{2}+e_{22}$ and $3e_{12}<3+4d+2e_{22}$, respectively. From the last together with $d>-\frac{1}{2}$ we conclude $e_{22}>1$. Also the signs of the coefficients of *s*(*u*) have to change, and both $-+-$ and $+-+$ are possible. We give the proof for the case $-+-$. By combining $s_0<0$, $s_1>0$, and $s_2<0$ with the above inequalities, we arrive at

A.19a $$\begin{aligned}&d> -\frac{1}{2} \text { and } e_{22}>1, \end{aligned}$$

A.19b $$\begin{aligned}&e_{12} < 2e_{22}+4d(d+e_{22}), \end{aligned}$$

A.19c $$\begin{aligned}&3e_{12} < 3+4d+2e_{22}, \end{aligned}$$

A.19d $$\begin{aligned}&2[e_{12}+(d+e_{22})(4d-e_{12}+e_{22})]< e_{11} \nonumber \\&\qquad < \frac{4d^2+6de_{12}-(e_{12}-3)e_{12}+2(e_{12}-1)e_{22}}{1+2d}, \end{aligned}$$

where the inequalities in (A.19d) are due to the conditions $s_1>0$ and $s_2<0$, and the right inequality in (A.19b) comes from $s_0<0$. However, we have not invoked $1<e_{12}<e_{11}-1-2d$. Simple rearrangements shows that (A.19d) is satisfied for some $e_{11}$ if and only if

$$\begin{aligned} (1+4d-e_{12}+2e_{22})(-e_{12}+2e_{22}+4d(d+e_{22}))<0 \end{aligned}$$

holds. (A.19b) implies that the second factor is positive. By multiplying the first factor with three, we can apply (A.19c) and find, using (A.19a),

$$\begin{aligned} 3+12d-3e_{12}+3e_{22}>4(2d+e_{22})>4(-1+1)=0. \end{aligned}$$

This yields the desired contradiction.

The proof for the sign sequence $+-+$ for $s_0$, $s_1$, and $s_2$ is very similar and left to the reader.

Next, we study the boundary flow ${\mathsf {E}}^2_2{\mathsf {C}}_0{\mathsf {s}}$. We can scale fitnesses such that $a+d=1$. In addition, we have $a+d>2a$, which implies $d>\tfrac{1}{2}$. The flow on the edge $p=1$ implies $e_{11}<2d-1+e_{12}$ and $e_{12}<-1$. Together with the assumption of three sign changes of *r*(*u*), we obtain the following conditions:

A.20a $$\begin{aligned}&\displaystyle \frac{1}{2}< d < -\frac{e_{22}}{2}, \end{aligned}$$

A.20b $$\begin{aligned}&\displaystyle e_{22} < -1, \end{aligned}$$

A.20c $$\begin{aligned}&\displaystyle \frac{1}{3}(-3+4d+2e_{22})< e_{12} < -1, \end{aligned}$$

A.20d $$\begin{aligned}&\displaystyle e_{11} < 2d-1+e_{12}. \end{aligned}$$

We will show that (A.20) implies $s_0>0$ and $s_2<0$, whence the pair of asymmetric internal equilibria never exists. Assume $s_0<0$. Then

$$\begin{aligned} 2e_{22}-4d(d+e_{22})<e_{12}<-1 \end{aligned}$$

holds, and $2e_{22}-4d(d+e_{22})<-1$ together with (A.20a) implies

$$\begin{aligned} \frac{-1+4d^2}{2(1-2d)}<e_{22}<-2d, \end{aligned}$$

which cannot be satisfied if $d>\tfrac{1}{2}$. Hence, $s_0>0$ must hold.

Now assume $s_2>0$. This yields the left inequality in

$$\begin{aligned} \frac{4d^2+e_{12}(6d-3-e_{12}+2e_{22})-2e_{22}}{2d-1}< e_{11} < 2d-1+e_{12}, \end{aligned}$$

and the right inequality is (A.20d). Then the inequality between the left and the right term can be rewritten as

$$\begin{aligned} (1+e_{12})(1-4d+e_{12}-2e_{22})>0, \end{aligned}$$

which is impossible because $1+e_{12}<0$ by (A.20b) and $1-4d+e_{12}-2e_{22}>0$ by (A.20c). This finishes the proof of the case ${\mathsf {E}}^2_2{\mathsf {C}}_0{\mathsf {s}}$. Because ${\mathsf {E}}^2_0{\mathsf {C}}_2{\mathsf {s}}$ is the flow reversal of ${\mathsf {E}}^2_2{\mathsf {C}}_0{\mathsf {s}}$, we have also excluded five internal equilibria for this case.

Finally we show, again by contradiction, that four internal equilibria are impossible for boundary flow class ${\mathsf {E}}^0_0{\mathsf {C}}_1{\mathsf {s}}$. Here we scale the fitnesses such that $a+d=-1$, and we have $d>-\frac{1}{2}$.

If there are four internal equilibria then, since in this case $r_0$ and $r_3$ are both positive, there are three possibilities for *r*(*u*) to have two sign changes. It is easily seen that $+--+$ is impossible. We give the proof only for $+-++$. This yields the following inequalities:

A.21a $$\begin{aligned} -\tfrac{1}{2}&< d < e_{22}-\frac{3}{2}, \end{aligned}$$

A.21b $$\begin{aligned} e_{12}&< 1 < e_{22}, \end{aligned}$$

A.21c $$\begin{aligned} e_{11}&<1+2d+e_{12} \;. \end{aligned}$$

Next we want to show that $s_0<0$ and $s_1<0$ if (A.21) holds.

Assume $s_0>0$. Using (A.21a) we get $\frac{-4d^2+e_{12}}{2(1+2d)}>e_{22}>d+\frac{3}{2}$. The outer inequality together with (A.21b) is equivalent to

$$\begin{aligned} 8(1+d)d+3<e_{12}<1, \end{aligned}$$

where now the inequality between the left and the right term implies $2(1+2d)^2<0$, which is a contradiction and therefore $s_0<0$.

Now assume $s_1>0$. Applying (A.21c), we get

$$\begin{aligned} 8 d^2 + 2 e_{12} - 2 d e_{12} + 12 d e_{22} - 2 e_{12} e_{22} + 4 e_{22}^2<e_{11}<1+2d+e_{12}. \end{aligned}$$

The inequality between the outer terms is equivalent to

$$\begin{aligned} (-1 + 2 d + 2 e_{22}) (1 + 4 d - e_{12} + 2 e_{22}) < 0. \end{aligned}$$

By using (A.21a) and subsequently (A.21b) for both factors, we find for the first

$$\begin{aligned} -1 + 2 d + 2 e_{22}>2(-1+e_{22})>0, \end{aligned}$$

and for the second

$$\begin{aligned} 1 + 4 d - e_{12} + 2 e_{22}>-1-e_{12}+2e_{22}<1-e_{12}>0. \end{aligned}$$

Thus we have again a contradiction and know that there can be at most one sign change in the polynomial *s*(*u*). Hence a pair of asymmetric equilibria is impossible under (A.21). The proof for the case $++-+$ is left to the reader. This finishes the proof for case ${\mathsf {E}}^0_0{\mathsf {C}}_1{\mathsf {s}}$.

### Proofs for the symmetric viability model

#### Proof of Theorem 9.1

Statement (a) of the theorem follows immediately from the dynamics (9.3). To prove (b), we need some preparation.

Let $l\ne 0$ and $r_1r_2\ne 0$. First, we compute the coordinates $y_{i,j}$ and $x_{i,j}$ of the potential unsymmetric equilibria. These are given by the intersection points of the isoclines (9.4). Since the central equilibrium (0, 0) exists always, we assume $(x,y)\ne (0,0)$. It follows immediately from (9.4) that then $x\ne 0$ and $y\ne 0$. Therefore, we can eliminate *x* and obtain after a short calculation

A.22 $$\begin{aligned} m^2\frac{r_1}{r_2}=\left( r_1+ly^2\right) ^2. \end{aligned}$$

Thus, (A.22) can have real solutions only if ${\text {sgn}}r_2={\text {sgn}}r_1$. This proves statement (iii) of Theorem 9.1.

From now on we assume ${\text {sgn}}r_2={\text {sgn}}r_1$. Taking square roots in (A.22), we obtain

A.23 $$\begin{aligned} \pm \left| m\right| \sqrt{\frac{r_1}{r_2}}=r_1+ly^2, \end{aligned}$$

and define

A.24 $$\begin{aligned} y_\pm = \frac{-r_1\pm \left| m\right| \sqrt{\frac{r_1}{r_2}}}{l}. \end{aligned}$$

If $y_\pm >0$, the corresponding square roots exist and yield the expressions $y_{i,j}$ in (9.5) of Theorem 9.1. Substituting the expressions $y_{i,j}$(9.5) into (9.4a) gives

A.25 $$\begin{aligned} x_{1,2}= \sqrt{\frac{r_2}{r_1}}y_{1,2} \quad \text {and} \quad x_{3,4}=-\sqrt{\frac{r_2}{r_1}}y_{3,4}. \end{aligned}$$

The following lemma lists the conditions for admissibility of the internal equilibria.

##### Lemma A.4

Define

A.26 $$\begin{aligned} h_i=r_i+l \quad \text {for}\quad i=1,2 \end{aligned}$$

and

A.27 $$\begin{aligned} \epsilon _i=\frac{r_i}{r_j}-\left( \frac{h_i}{m}\right) ^2 \quad \text {for}\quad i=1,2 \text { and } j\ne i. \end{aligned}$$

If $\left| r_1\right| \ge \left| r_2\right|$, Table 3 states the conditions under which each of $y_+$ and $y_-$ gives rise to a pair of internal equilibria. If $\left| r_1\right| <\left| r_2\right|$, an analogous table gives these conditions, in which the roles of *x* and *y* are exchanged, and $r_1$, $h_1$, and $\epsilon _1$ are substituted by $r_2$, $h_2$, and $\epsilon _2$, respectively.

Obviously, the coordinates of the equilibria are given by (9.5).

##### Proof

As shown above, the solutions are admissible only if ${\text {sgn}}r_2={\text {sgn}}r_1$, which we assume henceforth. The conditions in each of the cells are determined quite straightforwardly. For illustration, we prove the case $r_1>0$ and $l>0$, and leave the other cases to the reader. Because $r_1>0$ and $l>0$, only $y_+$ can give rise to equilibria. Because $\left| r_1\right| \ge \left| r_2\right|$, the resulting pair of equilibria is admissible if and only if $0<y_+<1$; cf. (9.5). Because $l>0$, $0<y_+<1$ is equivalent to

A.28 $$\begin{aligned} 0< -r_1 + \left| m\right| \sqrt{\frac{r_1}{r_2}}<l. \end{aligned}$$

Multiplication of the left inequality by $\sqrt{\frac{r_2}{r_1}}$ shows that the left inequality holds if and only if $\rho <0$. The right inequality is equivalent to

A.29 $$\begin{aligned} \left| m\right| \sqrt{\frac{r_1}{r_2}} < r_1 + l = h_1, \end{aligned}$$

where $h_1>0$. Squaring and rearranging shows that the inequality is equivalent to $\epsilon _1<0$. Thus, we have proved the conditions in the first row of the table.

The statement about the case $\left| r_1\right| <\left| r_2\right|$ follows immediately from the symmetry of *x* and *y* in the dynamics (9.3) and the according symmetry of $y_{i,j}$ and $x_{i,j}$. $\square$

Now we can finish the proof of Theorem 9.1. Statement (b)(i) follows immediately from the first two rows of Table 3, and statement (b)(ii) follows from the third and forth row. This finishes the proof.

**Table 3:** Each cell lists the conditions for the validity of the inequalities on top under the respective assumptions on $r_1$ and *l* on the left

	$0< y_+ < 1$	$0< y_- < 1$
$r_1>0 \wedge l>0$	$\rho<0 \wedge \epsilon _1<0$	−
$r_1<0 \wedge l<0$	−	$\rho<0 \wedge \epsilon _1<0$
$r_1> 0 > l$	$(\rho>0 \wedge h_1<0) \; \vee (\rho>0 \wedge h_1>0 \wedge \epsilon _1>0)$	$h_1<0 \wedge \epsilon _1<0$
$r_1< 0 < l$	$h_1>0 \wedge \epsilon _1>0$	$(\rho>0 \wedge h_1>0)\; \vee (\rho>0 \wedge h_1<0 \wedge \epsilon _1>0)$

The symbols $\wedge$ and $\vee$ mean ‘and’ and ‘or’

#### Proof of Theorem 9.5

Before proving the theorem, we present a number of results that will be needed. We begin by presenting the coordinates and eigenvalues of the SLP’s in (*x*, *y*)-coordinates:  The internal eigenvalues are

A.31a $$\begin{aligned} \mu _{34}&=\mu _{12}=\frac{h_2^2-m^2}{h_2}, \end{aligned}$$

A.31b $$\begin{aligned} \mu _{24}&=\mu _{13}=\frac{h_1^2-m^2}{h_1}, \end{aligned}$$

and the external eigenvalues are

A.32a $$\begin{aligned} \hat{\lambda }_{34}&=\hat{\lambda }_{12}=\frac{r_2}{h_2^2}-\frac{r_1}{m^2}, \end{aligned}$$

A.32b $$\begin{aligned} \hat{\lambda }_{24}&=\hat{\lambda }_{13}=\frac{r_1}{h_1^2}-\frac{r_2}{m^2}. \end{aligned}$$

The following lemmas will help to keep the proof simple.

##### Lemma A.5

- $E_{34}$ and $E_{12}$ are admissible if and only if $\left( \frac{h_2}{m}\right) ^2>1$.
- $E_{24}$ and $E_{13}$ are admissible if and only if $\left( \frac{h_1}{m}\right) ^2>1$.

These statements follow immediately from (A.30).

##### Lemma A.6

Let $${\text {sgn}}r_2={\text {sgn}}r_1$$. Then

- $\hat{\lambda }_{34}=\hat{\lambda }_{12}>0$ if and only if ${\text {sgn}}\epsilon _2={\text {sgn}}r_2$.
- $\hat{\lambda }_{24}=\hat{\lambda }_{13}>0$ if and only if ${\text {sgn}}\epsilon _1={\text {sgn}}r_2$.

These statements follow easily from (A.32) and the definition of $\epsilon _i$ (A.27).

##### Lemma A.7

Let $${\text {sgn}}r_2={\text {sgn}}r_1$$ and $$r_1\ne r_2$$.

- If $E_{24}$ and $E_{13}$ are admissible and ${\text {sgn}}\hat{\lambda }_{24}={\text {sgn}}\hat{\lambda }_{13}=-{\text {sgn}}(r_1-r_2)$, then ${\text {sgn}}r_2={\text {sgn}}(r_1-r_2)$.
- If $E_{34}$ and $E_{12}$ are admissible and ${\text {sgn}}\hat{\lambda }_{34}={\text {sgn}}\hat{\lambda }_{12}={\text {sgn}}(r_1-r_2)$, then ${\text {sgn}}r_2=-{\text {sgn}}(r_1-r_2)$.

##### Proof

Let ${\text {sgn}}(r_1-r_2)=-1$. Then $r_2>r_1$ and the following implications hold:

A.33 $$\begin{aligned} {\text {sgn}}r_1&>0 \Rightarrow m^2r_1-h_1^2r_2<r_1(m^2-h_1^2)<0 \Leftrightarrow \hat{\lambda }_{24}=\hat{\lambda }_{13}<0 \end{aligned}$$

and

A.34 $$\begin{aligned} {\text {sgn}}r_2&<0 \Rightarrow m^2r_2-h_2^2r_1>r_2(m^2-h_2^2)>0 \Leftrightarrow \hat{\lambda }_{34}=\hat{\lambda }_{12}>0. \end{aligned}$$

Here, we used that $m^2-h_1^2<0$ ($m^2-h_1^2<0$) if $E_{24}$ and $E_{13}$ ($E_{34}$ and $E_{12}$) are admissible (Lemma A.5). By reversing the inequalities and the implications in (A.33) and (A.34), we get statements (a) and (b) for the case ${\text {sgn}}(r_1-r_2)=-1$. If ${\text {sgn}}(r_1-r_2)=+1$, a similar argument applies. $\square$

These lemmas help us to prove the following statements.

##### Corollary A.8

- For ${\mathsf {E}}^4_4{\mathsf {C}}_0{\mathsf {b}}$ with $\delta =4$ and $\rho >0$, *O* is a source.
- For ${\mathsf {E}}^4_4{\mathsf {C}}_0{\mathsf {b}}$ with $\delta =2$ and $\rho >0$, *O* is a source.

##### Proof

Without loss of generality (cf. Lemma A.4), we can assume $\left| r_1\right| \ge \left| r_2\right|$. Because we assume $\rho >0$, the index of *O* is $+1$.

- Since $\delta =4$ is assumed, there must be four internal equilibria, in addition to *O*. Therefore, Theorem 9.1 implies that ${\text {sgn}}r_1={\text {sgn}}r_2=-{\text {sgn}}l$. To achieve $\delta =4$, we need ${\text {sgn}}\hat{\lambda }_{34}= {\text {sgn}}\hat{\lambda }_{12}= {\text {sgn}}\hat{\lambda }_{24}={\text {sgn}}\hat{\lambda }_{13}=-1$. Suppose $r_1<r_2<0$. Then ${\text {sgn}}(r_1-r_2)=-1$ and Lemma A.7(b) informs us that ${\text {sgn}}r_2=-{\text {sgn}}(r_1-r_2)=1$, a contradiction. Therefore, $l<0<r_2\le r_1$ and ${\text {tr}}J^o=r_1+r_2>0$ (9.8b). Because $\rho >0$, the central equilibrium is a source.
- Assume *O* is a sink. Then $r_1+r_2<0$. Because $\delta =2$ and the index of *O* is $+1$, there must be at least two additional internal equilibria. Theorem 9.1 implies that ${\text {sgn}}r_1={\text {sgn}}r_2$, whence ${\text {sgn}}r_1={\text {sgn}}r_2=-1$ follows. Table 3 from Lemma A.4 shows that $l>0$ is necessary for the existence of at least two additional internal equilibria. Because $\delta =2$ and no corner equilibrium is saturated, we have ${\text {sgn}}(\hat{\lambda }_{13})=-{\text {sgn}}(\hat{\lambda }_{12})$. Lemma A.6 shows that only $\hat{\lambda }_{12}>0$ is compatible with $r_1<r_2<0$, because otherwise statement (a) yields $\epsilon _2>0$, which implies $r_2<\bigl (\frac{m}{h_1}\bigr )^2 r_1<r_1$, a contradiction (here, the second inequality follows from by Lemma A.5b). From the boundary flow itself, we get the inequalities
  A.35a $$\begin{aligned} r_2+l+m<&0>r_2+l-m, \end{aligned}$$
  A.35b $$\begin{aligned} h_1,h_2<&0. \end{aligned}$$

This yields

A.36 $$\begin{aligned} 0>-l>r_2>r_1. \end{aligned}$$

We define $\omega (r_1) = h_1^2m^2\hat{\lambda }_{13} = r_1m^2-r_2(r_1+l)^2$. Then $\omega (0)=-r_2l^2>0$, $\omega (-l)=-lm^2<0$, and $\omega (r_2)=-r_2(r_2+l-m)(r_2+l+m)>0$ by (A.35a). Since $\omega$ is quadratic in $r_1$ with the positive leading coefficient $-r_2$, it follows that $\omega (r_1)>0$ for $r_1<r_2$. This yields the desired contradiction because ${\text {sgn}}(\omega (r_1))={\text {sgn}}(\hat{\lambda }_{13})<0$. $\square$

Now we are able to prove Theorem 9.5.

##### Proof

We prove the statements about the internal equilibrium structures in Table 2 column by column. Table S1 shows that the boundary flows in Table 2 marked by ‘−’ cannot by realized for the given $\delta$. Therefore, it is sufficient to validate the cells with non-trivial entries and the one marked by ‘$\times$’. For each of these cells we show that the given equilibrium structures are the only possible. Existence follows from the corresponding figures in Section S1 of the SI.

**Case 1**, ${\varvec{\rho }}<{\varvec{0}}$ Lemma A.4 and Table 3 show that at most one pair of unsymmetric internal equilibria can exist. Thus, the number of internal equilibria is one or three. Lemma 9.2 ensures that *O* is a saddle with index $-1$.

${\varvec{\delta }}={\varvec{0}}$ To achieve an index sum of $+1$ from the internal equilibria, one pair of unsymmetric equilibria with index $+1$ must exist. Three internal equilibria can indeed be realized for the four indicated boundary flows. The two unsymmetric equilibria must be sinks for the boundary flows ${\mathsf {E}}^2_2{\mathsf {C}}_0{\mathsf {c}}$ and ${\mathsf {E}}^4_4{\mathsf {C}}_0{\mathsf {b}}$, because otherwise no stable equilibrium would exist. The two flow reversal cases follow immediately.

It remains to prove that for the boundary-flow class ${\mathsf {E}}^4_2{\mathsf {C}}_0{\mathsf {c}}$ two unsymmetric equilibria cannot exist. Unsymmetric equilibria exist only if ${\text {sgn}}r_1={\text {sgn}}r_2$ (Theorem 9.1). Because for one pair of edge equilibria the internal eigenvalues are negative and for the other positive, and because $\bigl (\frac{h_i}{m}\bigr )^2>1$ by Lemma A.5, Eq. (A.31) implies ${\text {sgn}}h_1\ne {\text {sgn}}h_2$. Therefore, $r_1\ne r_2$. Without loss of generality we assume $\left| r_1\right| >\left| r_2\right|$. If $r_1>r_2>0$, then $h_1=r_1+l > r_2+l =h_2$ and $h_1>0>h_2$. This implies $r_1>0>l$. Because $\rho <0$, Table 3 shows that two unsymmetric equilibria could exist only if $h_1<0$, which is impossible. If $r_1<r_2<0$, then $l>0$ and $h_1<0$ follow. Again, Table 3 shows that two unsymmetric equilibria cannot exist. Therefore, this boundary flow cannot be realized if $\rho <0$ and $\delta =0$.

${\varvec{\delta }} ={\varvec{2}}$ Three internal equilibria cannot be realized because at most one pair of unsymmetric equilibria can exist, and each of these equilibria has the same index (see Sect. 9 below Eq. 9.3). Thus, a total index sum of 1 can be realized only if *O* is the unique internal equilibrium.

${\varvec{\delta }}={\varvec{4}}$ Here, one pair of unsymmetric equilibria, each with index $-1$, must exist to achieve a total index sum of 1.

**Case 2**, ${\varvec{\rho }}>{\varvec{0}}$ Lemma 9.2 shows that *O* has index 1.

${\varvec{\delta }}={\varvec{4}}$ Four saddles need to exist in the interior to realize this case. Corollary A.8 informs us that *O* is a source.

${\varvec{\delta }}={\varvec{2}}$ The sum of the indices of the unsymmetric equilibria must be $-2$. This can be realized only if there is one pair of unsymmetric equilibria, because the two members of each pair have the same index. Corollary A.8 informs us that *O* is a source for ${\mathsf {E}}^4_4{\mathsf {C}}_0{\mathsf {b}}$, whence *O* is a sink in the flow-reversal case ${\mathsf {E}}^4_0{\mathsf {C}}_4{\mathsf {b}}$. In the other four cases, *O* can be a sink or a source.

${\varvec{\delta }}={\varvec{0}}$ We already know that *O* has index is 1. For ${\mathsf {E}}^2_2{\mathsf {C}}_0{\mathsf {c}}$ and ${\mathsf {E}}^4_4{\mathsf {C}}_0{\mathsf {b}}$ it must be a sink because otherwise no stable equilibrium would exist. This also settles the two flow reversal cases ${\mathsf {E}}^2_0{\mathsf {C}}_2{\mathsf {c}}$ and ${\mathsf {E}}^4_0{\mathsf {C}}_4{\mathsf {b}}$. For ${\mathsf {E}}^4_2{\mathsf {C}}_0{\mathsf {c}}$, *O* can be a sink or a source.

If more than one internal equilibrium exists, then ${\text {sgn}}r_1 = {\text {sgn}}r_2$ by Theorem 9.1. Without loss of generality (Lemma A.4), we assume $\left| r_1\right| \ge \left| r_2\right|$. Table 3 shows that because $\rho >0$, we must have ${\text {sgn}}r_1 = {\text {sgn}}r_2 = -{\text {sgn}}l$.

Assume three internal equilibria. Because *O* has index 1, the indices of the two unsymmetric equilibria must be of different sign, which is impossible in the symmetric viability model.

Assume five internal equilibria. Then Theorem 9.1 implies ${\text {sgn}}r_1={\text {sgn}}r_2=-{\text {sgn}}l$. Table 3 shows that this implies ${\text {sgn}}\epsilon _1 = {\text {sgn}}h_1 = -{\text {sgn}}r_1$. By Lemma A.6, this implies $\hat{\lambda }_{24}=\hat{\lambda }_{13}<0$.

We need to treat the boundary-flows classes separately. Because of the flow-reversal cases, it is sufficient to study ${\mathsf {E}}^2_2{\mathsf {C}}_0{\mathsf {c}}$, ${\mathsf {E}}^4_4{\mathsf {C}}_0{\mathsf {b}}$, and ${\mathsf {E}}^4_2{\mathsf {C}}_0{\mathsf {c}}$. The boundary flow ${\mathsf {E}}^4_4{\mathsf {C}}_0{\mathsf {b}}$ can have $\delta =0$ only if no edge equilibrium is saturated, i.e., if all external eigenvalues are positive. This contradicts $\hat{\lambda }_{24}=\hat{\lambda }_{13}<0$.

For ${\mathsf {E}}^4_2{\mathsf {C}}_0{\mathsf {c}}$, $\delta =0$ can be realized also if there are four saturated edge equilibria. Lemma A.6 implies ${\text {sgn}}\epsilon _1={\text {sgn}}\epsilon _2=-{\text {sgn}}r_2=-{\text {sgn}}r_1$. Because one pair of internal eigenvalues is negative and the other positive, and $\bigl (\frac{h_i}{m}\bigr )^2>1$ by Lemma A.5, equation (A.31) implies ${\text {sgn}}h_2 = -{\text {sgn}}h_1$. Assume $r_1\ge r_2>0$. Then $h_1=r_1+l>r_2+l=h_2$ and Table 3 shows that five internal equilibria require $h_1<0$. Therefore, $h_2<0$, which contradicts ${\text {sgn}}h_2 = -{\text {sgn}}h_1$. An analogous argument applies if $r_1\le r_2<0$.

It remains to exclude five internal equilibria for ${\mathsf {E}}^2_2{\mathsf {C}}_0{\mathsf {c}}$. Assume that $E_{12}$ and $E_{34}$ are admissible. Then they are internally stable. From the fitness scheme (9.1) we infer $r_1>r_2$ and $h_2 < -\left| m\right| \le 0$. Because $\delta =0$, these edge equilibria must be externally unstable, whence ${\text {sgn}}\epsilon _2 = -{\text {sgn}}r_2$ follows from Lemma A.6. Lemma A.7(b) shows that $r_2<0$, whence $r_2<0<l$ and $\epsilon _2>0$ follow. The analog of Table 3 for the case $\left| r_2\right| >\left| r_1\right|$ shows that five equilibria require $h_2>0$, a contradiction. $\square$
