# Supplementary material for: Evolutionary dynamics in the two-locus two-allele model with weak selection
Source: J Math Biol. 2017 May 25;76(1):151–203. doi: 10.1007/s00285-017-1140-7 (PMC5754571; doi:10.1007/s00285-017-1140-7)
Supplement: Supplementary file 1 — Supplementary material 1 (pdf 9323 KB) [file 285_2017_1140_MOESM1_ESM.pdf]

Supplementary Information: Evolutionary dynamics in the two-locus two-allele model with weak selection

Martin Pontz Josef Hofbauer Reinhard Bürger

May 22, 2017

Address for correspondence:

Martin Pontz
Institut für Mathematik
Universität Wien
Oskar-Morgenstern-Platz 1
1090 Wien
Austria
E-mail: martin.pontz@univie.ac.at
Phone: +43 1 4277 50784

S1 Phase portraits

The figures in this section display, up to flow reversal, phase portraits for $75 + 25$ possible extended boundary flows (see the explanation in Section 4.3). We show (at least) one representative of a flow-reversal pair from each of the 25 equivalence classes (with respect to topology and symmetry, as described in the first paragraphs of Section 4). For the 75 flow-reversal pairs we show only phase portraits yielding the upper type in Figure 2. For several extended boundary flows, we present more than one phase portrait. We do this, in particular, for cases where three, four, or five internal equilibria are compatible with a given extended boundary-flow class. Black dots represent sinks, black circles sources, and green dots (grey in black-white printing) are saddles.

Below each phase portrait, we provide the fitness matrix generating it. We found most matrices by using the built-in FindInstance function of *Mathematica*, which we applied to the set of inequalities that the parameters have to satisfy to yield the desired type of phase portraits. Entries of the computed matrices are usually rational numbers, often ratios of large integers. In most cases, we could find matrices in a neighborhood with integer entries that yield the same type of phase portrait, which were then used. In addition, we tried to choose the matrices such that the resulting phase portraits display the main features of the given external boundary flow as clearly as possible. In a few cases, no visually entirely satisfactory result could be achieved. We determined position and stability of the equilibria by numerical solution of the system resulting from a given matrix. We produced the graphs by combining the StreamPlot function for the orbits with the ListPlot function for the equilibria. Both are built-in functions of *Mathematica*.

Concerning robustness, the following qualification needs to be made. Some phase portraits of the symmetry classes b, s and e are robust only with respect to their symmetry property because they were generated by a matrix satisfying the respective symmetry condition. They may have saddle connections in the interior. In most of these cases, however, breaking the symmetry yields phase portraits that are still members of the same class.

Finally, we note that many phase portraits were generated by matrices that do not satisfy the symmetry assumption x of their boundary-flow class $\mathsf{E}^n_m\mathsf{C}_k\mathsf{x}$. The reason is that although there exists a matrix with symmetry property x generating a phase portrait of the given boundary-flow class and given δ, there also exist topologically non-equivalent phase portraits that belong to the same boundary-flow class and yield the same δ, but do not satisfy the symmetry property as such, i.e., they yield only a topologically equivalent boundary flow.

Figure S1a: $\delta = 0$

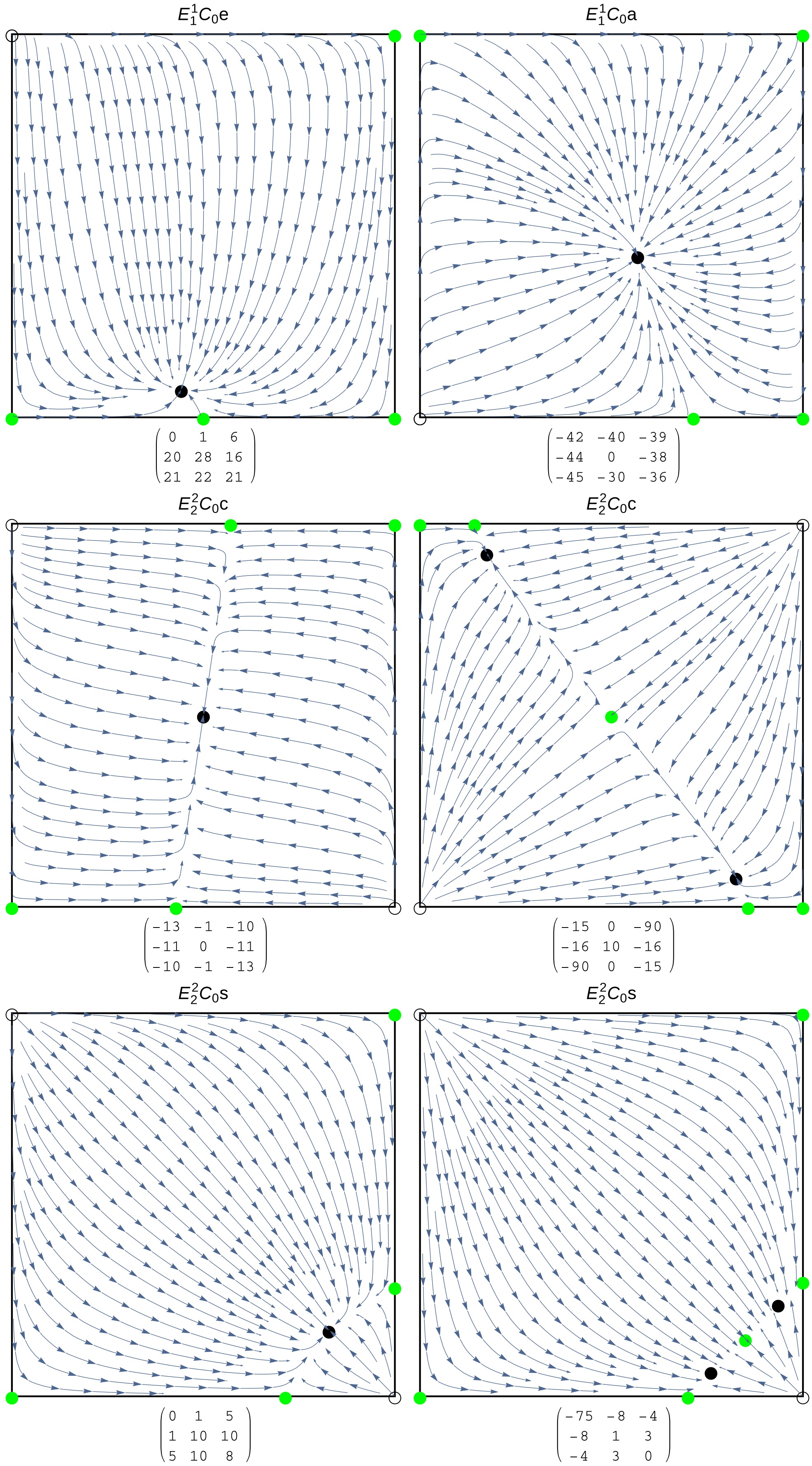

Figure S1b: $\delta = 0$

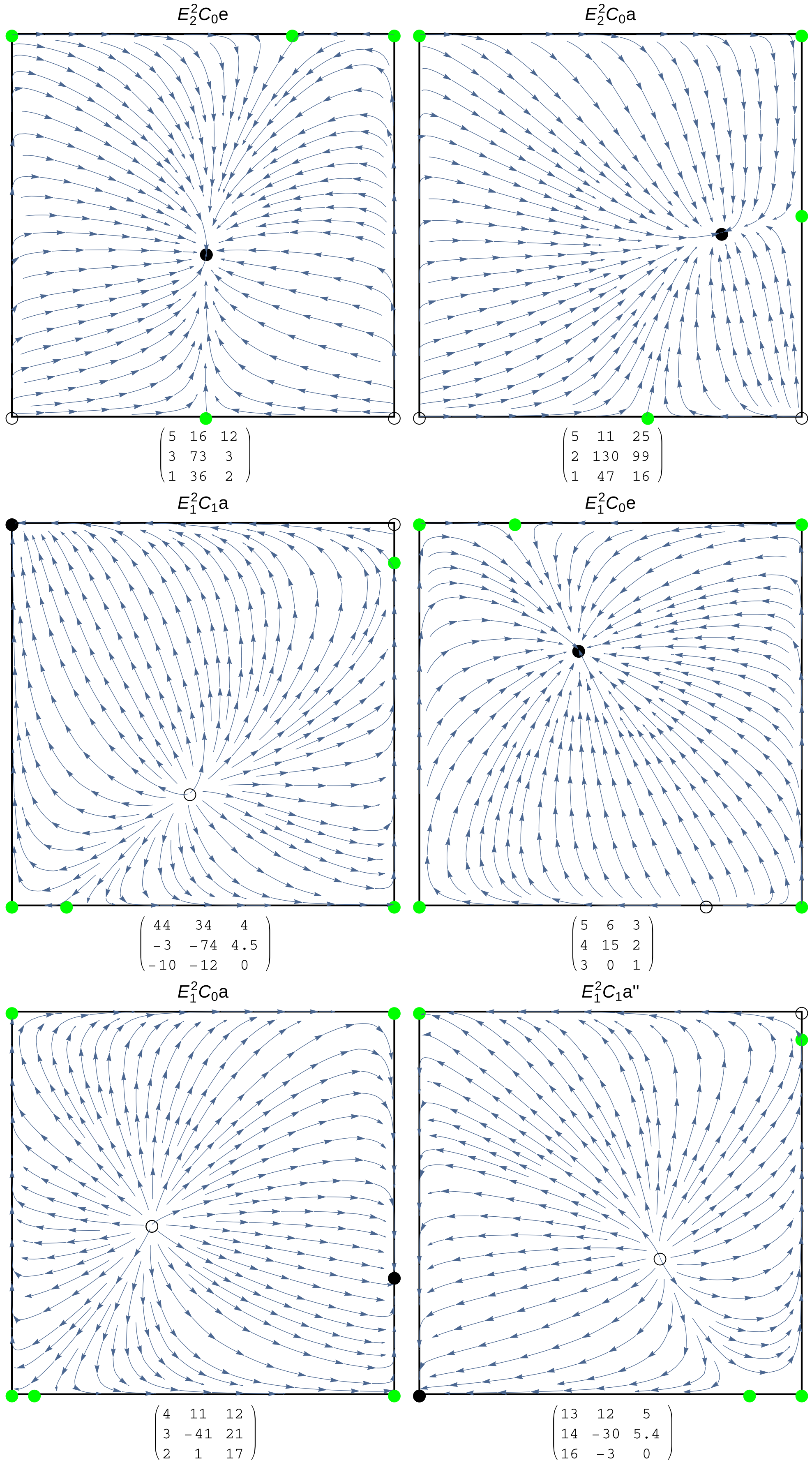

Figure S1c: $\delta = 0$

$E_1^2C_1\text{a}'''$

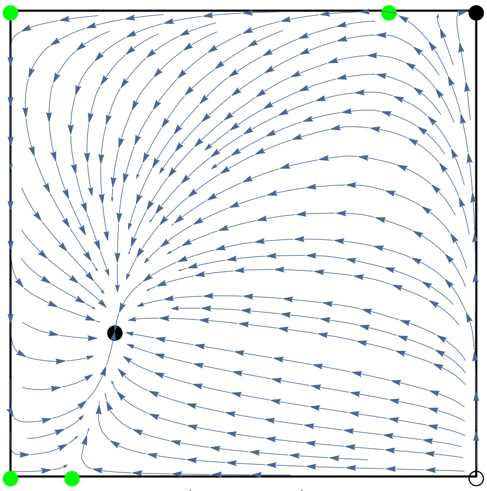

$$\begin{pmatrix} 37 & 24 & 27 \\ 38 & 65 & 5 \\ 39 & 46 & 0 \end{pmatrix}$$

$E_3^3C_0\text{e}$

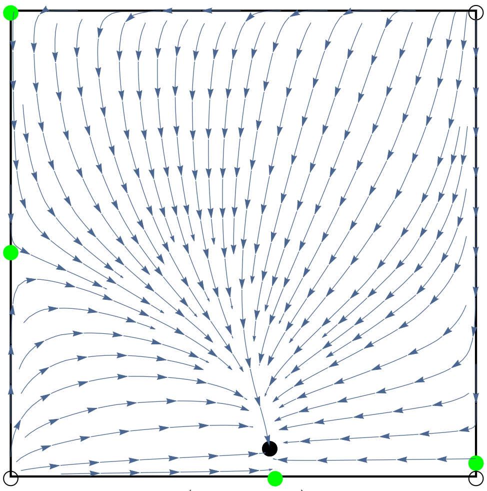

$$\begin{pmatrix} 15 & 9 & 8 \\ 50 & 90 & 37 \\ 17 & 96 & 36 \end{pmatrix}$$

$E_2^3C_0\text{e}$

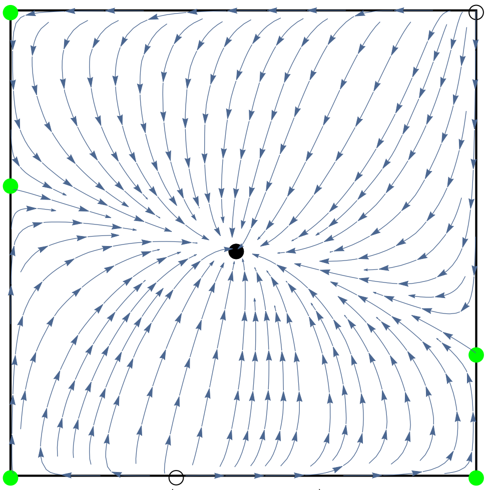

$$\begin{pmatrix} 65 & -1 & -15 \\ 106 & 394 & 113 \\ 37 & 0 & 67 \end{pmatrix}$$

$E_2^3C_0\text{e}$

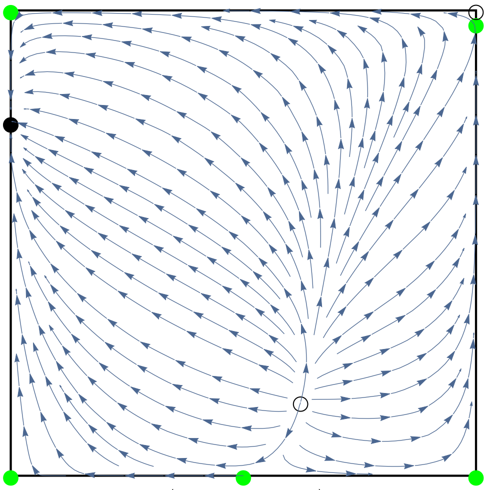

$$\begin{pmatrix} 30 & 14 & 10 \\ 44 & -34 & 10.3 \\ 0 & -4 & 0 \end{pmatrix}$$

$E_2^3C_0\text{e}$

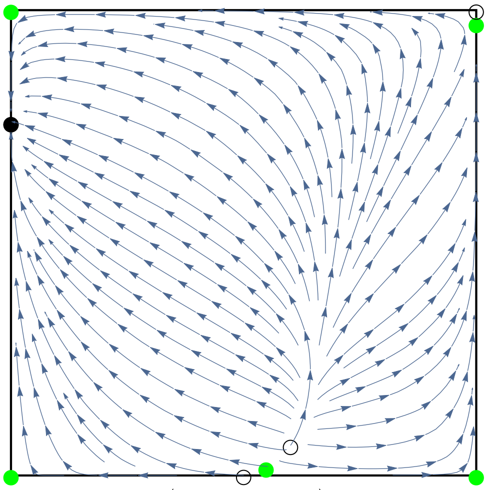

$$\begin{pmatrix} 30 & 14 & 10 \\ 44 & -29 & 10.3 \\ 0 & -4 & 0 \end{pmatrix}$$

$E_2^3C_0\text{a}$

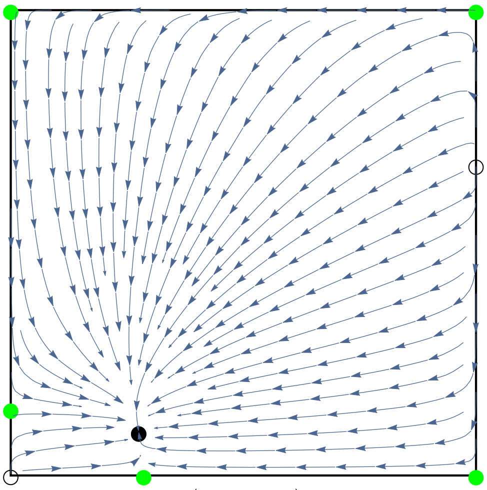

$$\begin{pmatrix} 4 & 3 & 2 \\ 10 & 14 & 1 \\ 9 & 13 & 3 \end{pmatrix}$$

Figure S1d: $\delta = 0$

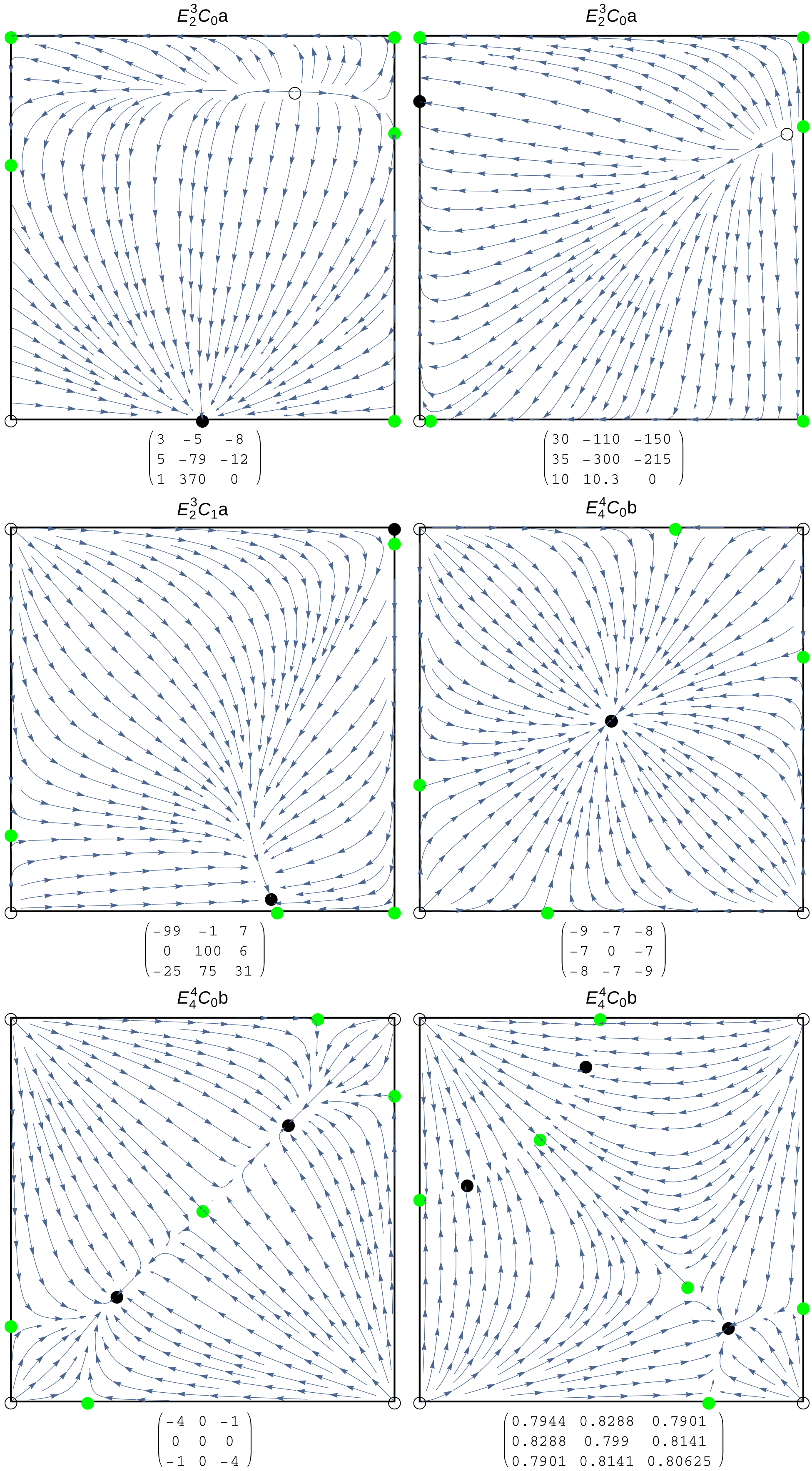

Panel 6 is the example given by Moran. It exhibits a saddle connection which is robust within the class of symmetric matrices, (8.1).

Figure S1e: $\delta = 0$

$E_3^4C_0$e

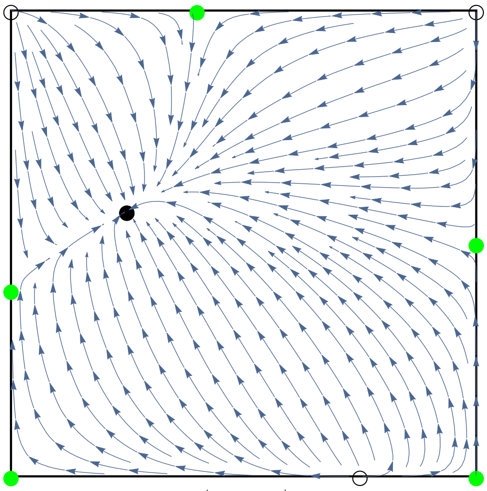

$$\begin{pmatrix} 2 & 4 & 1 \\ 5 & 7 & 2 \\ 3 & 0 & 1 \end{pmatrix}$$

$E_3^4C_0$e

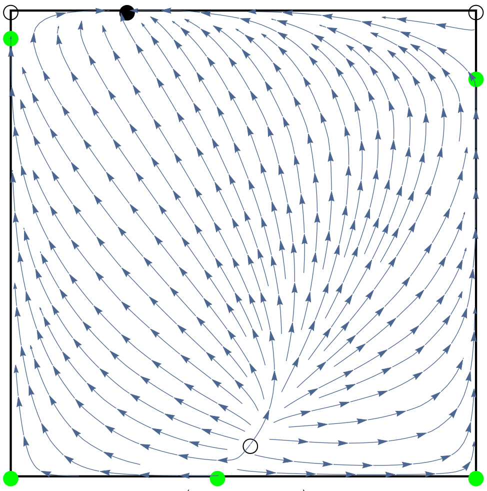

$$\begin{pmatrix} 15 & 20 & 5 \\ 16 & -19 & 6 \\ -1 & -5 & 0 \end{pmatrix}$$

$E_3^4C_0$e

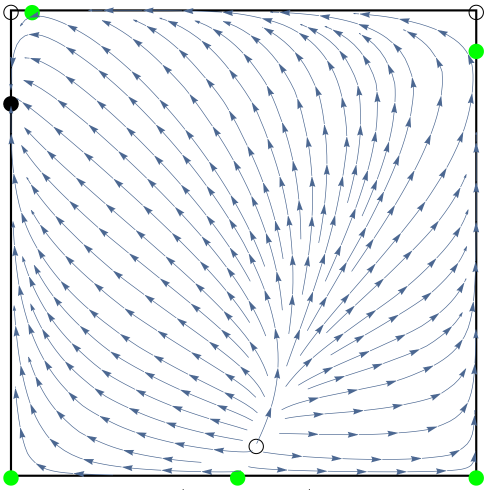

$$\begin{pmatrix} 30 & 31 & 10 \\ 40 & -50 & 11 \\ -1 & -20 & 0 \end{pmatrix}$$

$E_2^4C_0$c

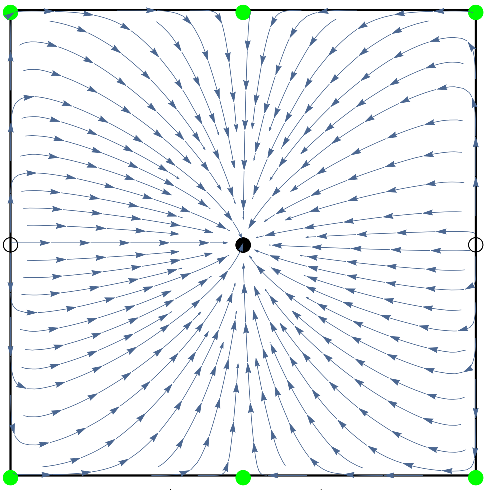

$$\begin{pmatrix} -11 & -10 & -11 \\ -12 & 0 & -12 \\ -11 & -10 & -11 \end{pmatrix}$$

$E_2^4C_1$s

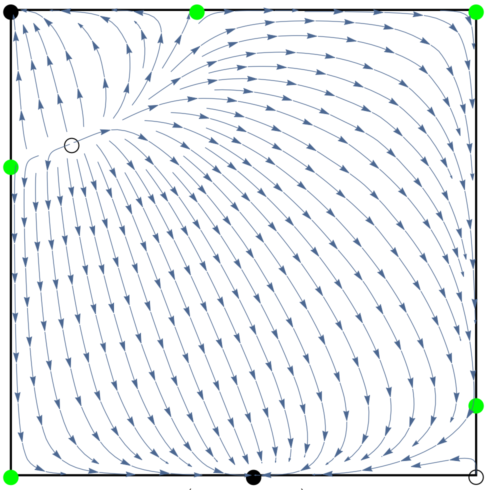

$$\begin{pmatrix} 12 & 8 & 14 \\ 2 & 0 & 25 \\ 22 & 34 & 23 \end{pmatrix}$$

Figure S2a: $\delta = 1$

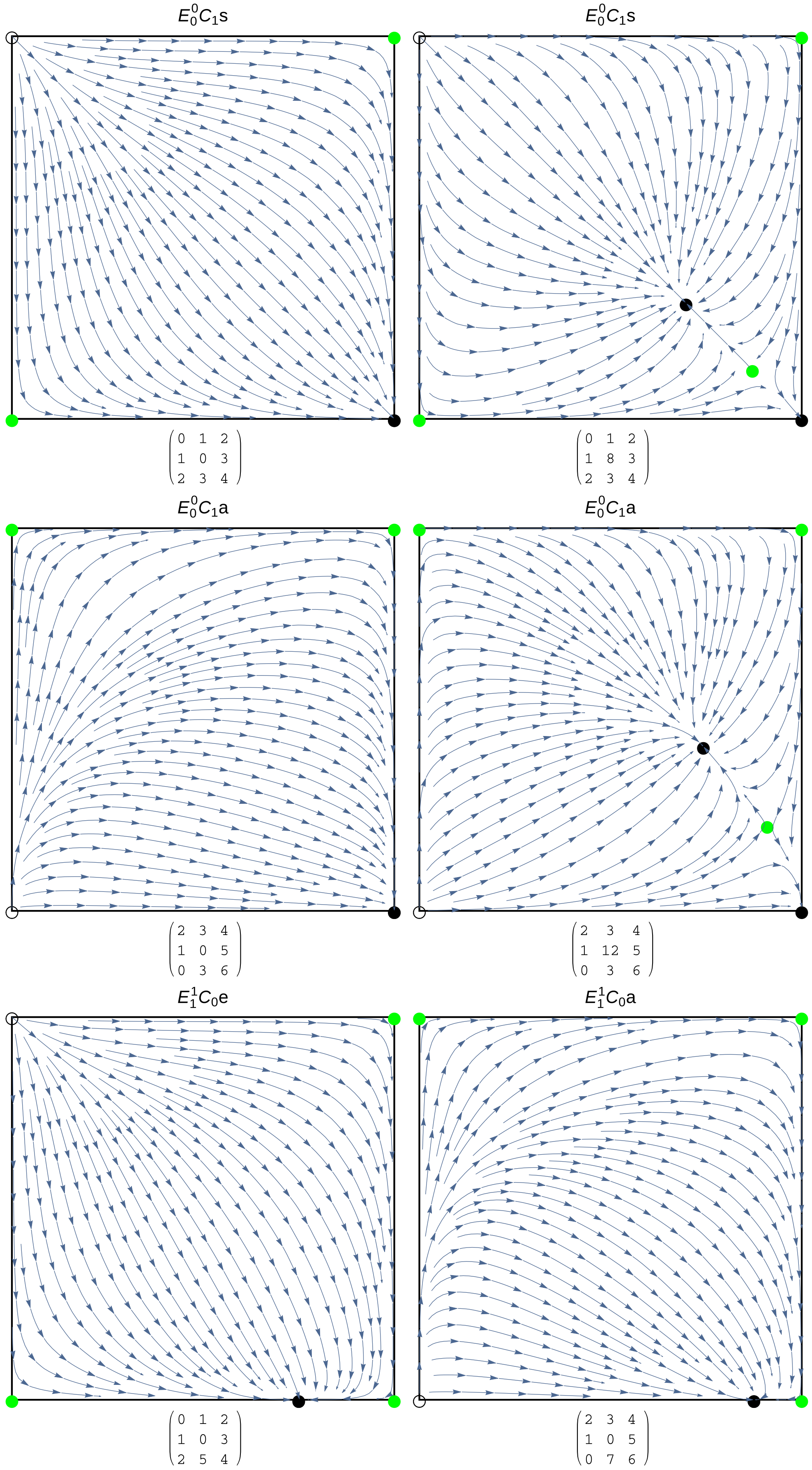

Figure S2b: $\delta = 1$

$E_1^1 C_0$a

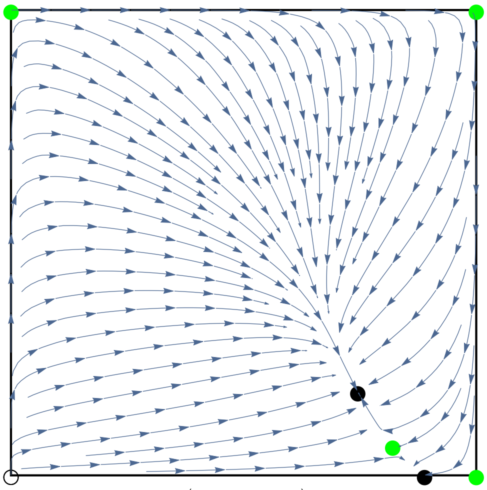

$$\begin{pmatrix} 2 & 3 & 4 \\ 1 & 13.3 & 5 \\ 0 & 8 & 7 \end{pmatrix}$$

$E_1^1 C_1$e

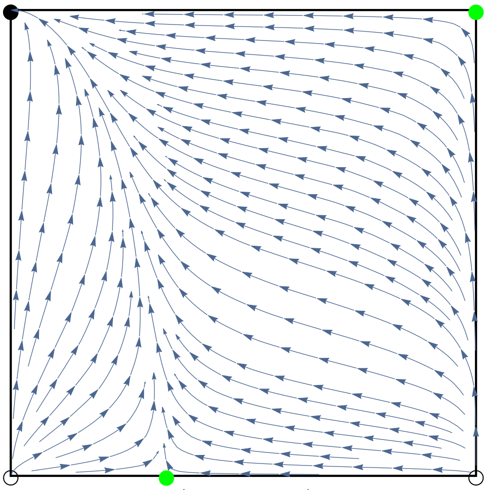

$$\begin{pmatrix} 81 & 71 & 0 \\ 50 & 93 & -41 \\ 0 & 84 & -83 \end{pmatrix}$$

$E_1^1 C_1$a

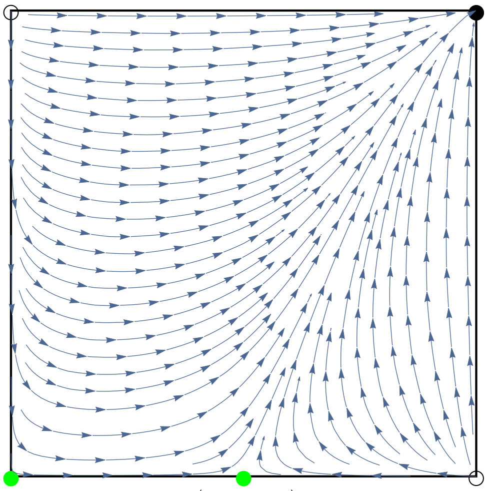

$$\begin{pmatrix} -2 & 4 & 6 \\ -1 & 4 & 3 \\ 0 & 2 & 0 \end{pmatrix}$$

$E_1^1 C_1$a

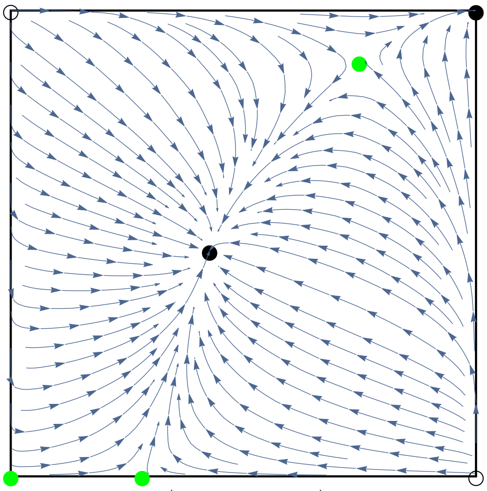

$$\begin{pmatrix} -10 & 0 & 59 \\ -8 & 240 & -86 \\ 0 & 63 & -97 \end{pmatrix}$$

$E_2^2 C_0$c

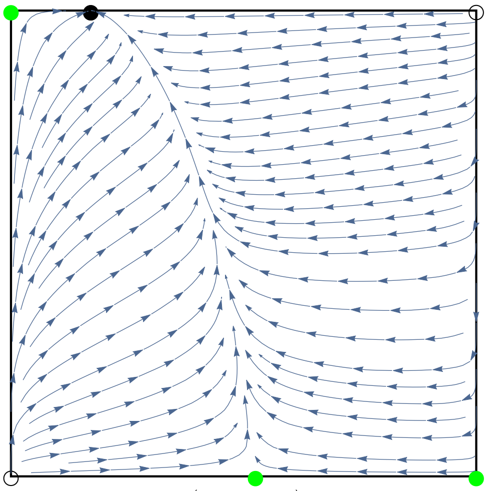

$$\begin{pmatrix} 46 & 58 & 0 \\ 15 & 86 & 2 \\ 1 & 63 & 7 \end{pmatrix}$$

$E_2^2 C_0$s

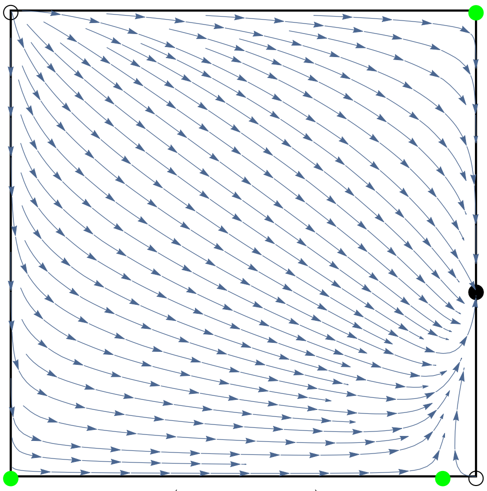

$$\begin{pmatrix} -19 & -18 & -3 \\ -17 & -1 & 0 \\ -14 & -1 & -2 \end{pmatrix}$$

Figure S2c: $\delta = 1$

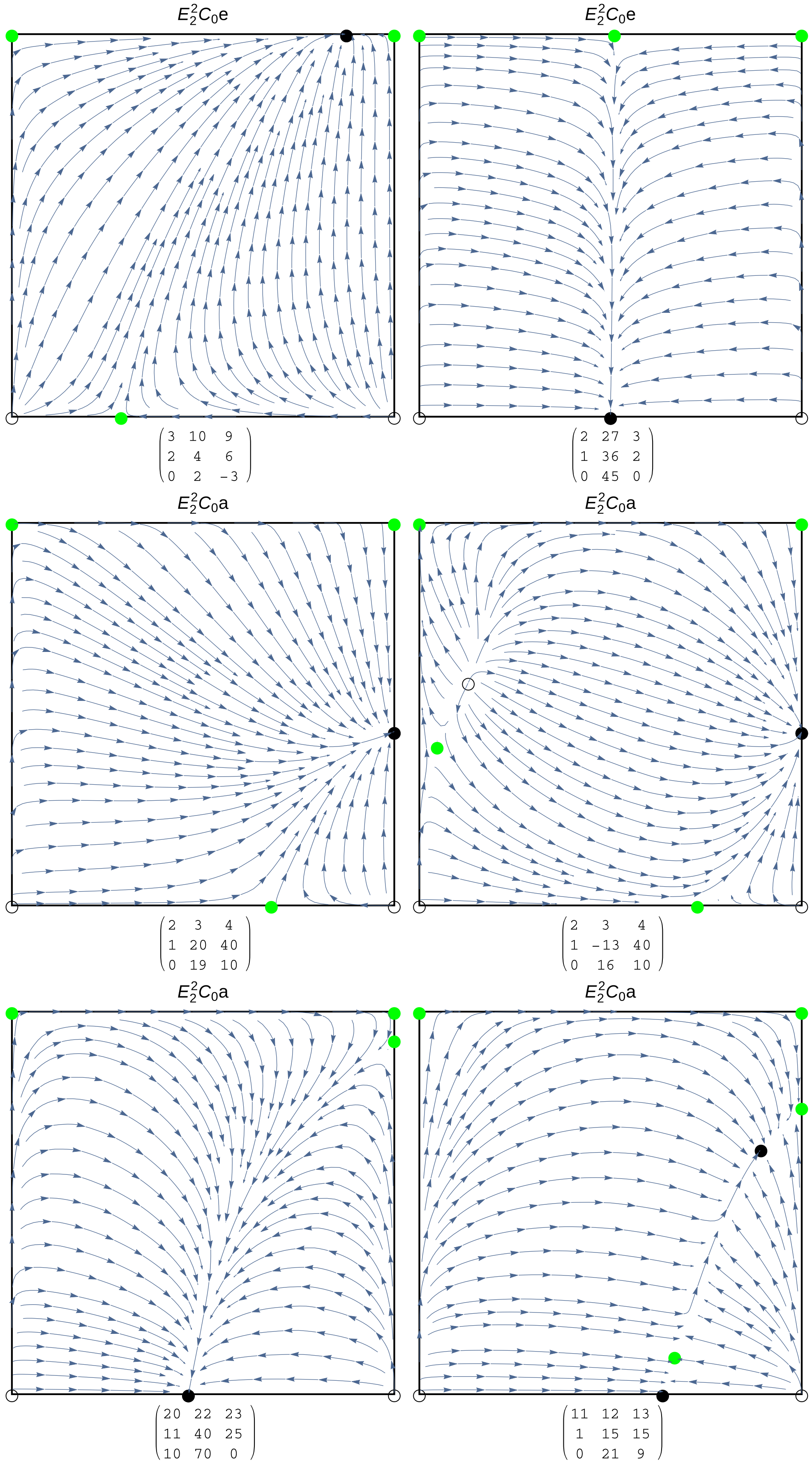

Figure S2d: $\delta = 1$

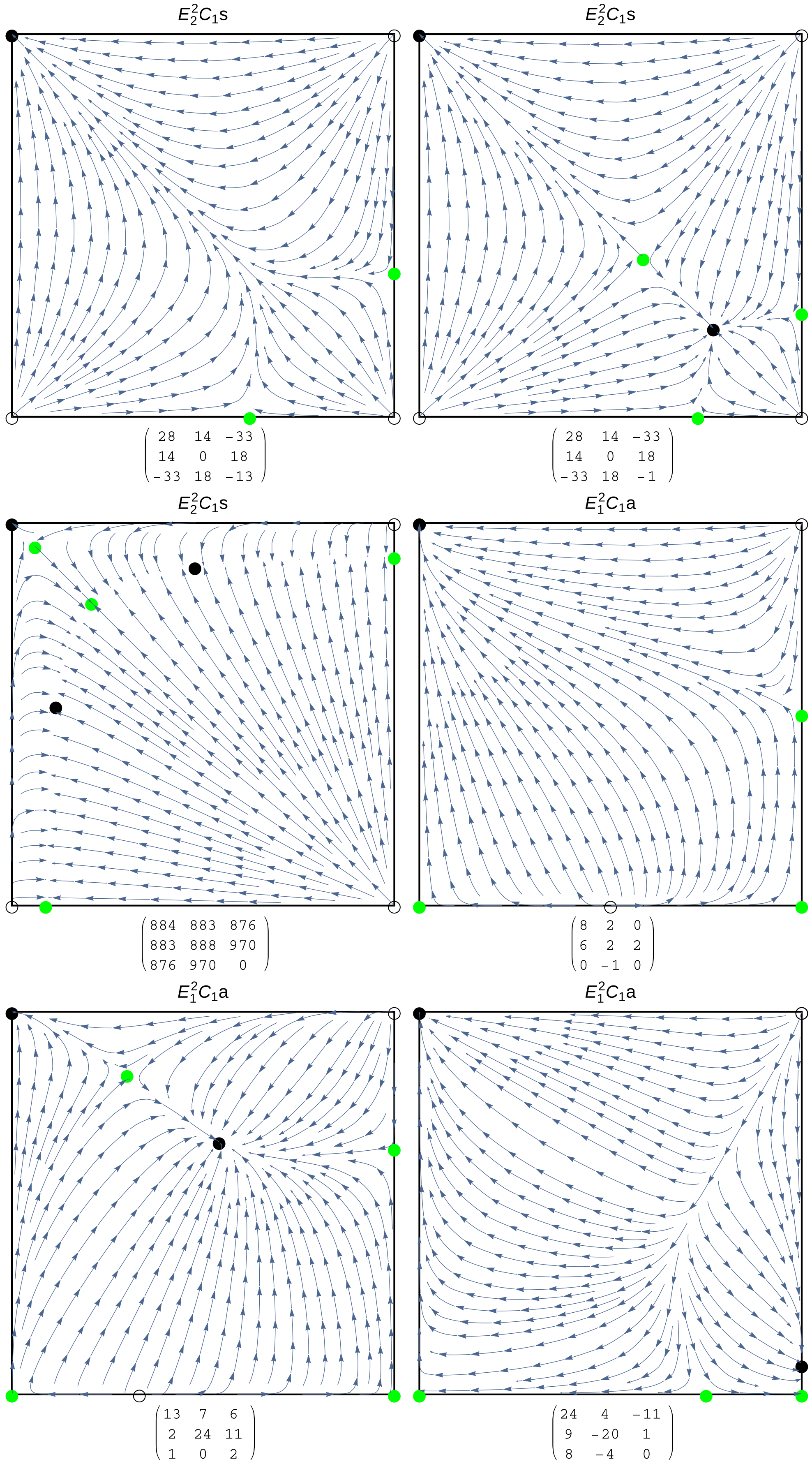

Figure S2e: $\delta = 1$

$E_1^2C_1$a

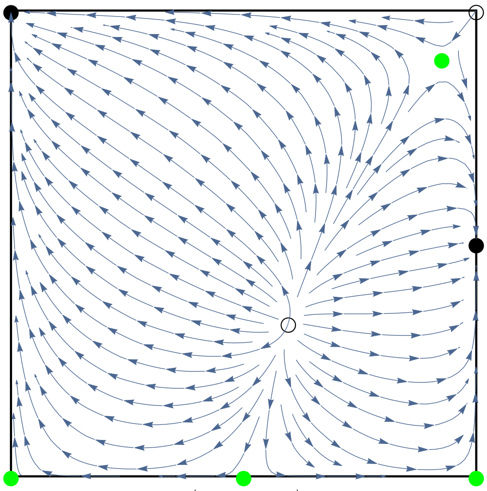

$$\begin{pmatrix} 8 & 2 & 0 \\ 6 & -10 & 2 \\ 0 & -1 & 0 \end{pmatrix}$$

$E_1^2C_0$e

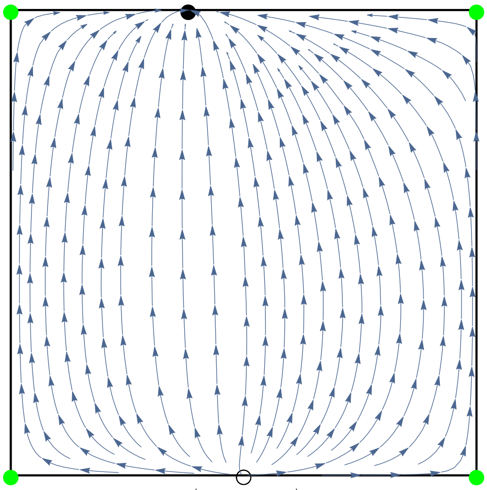

$$\begin{pmatrix} 10 & 18 & 5 \\ 2 & 0 & 3 \\ 1 & 0 & 1 \end{pmatrix}$$

$E_1^2C_0$a

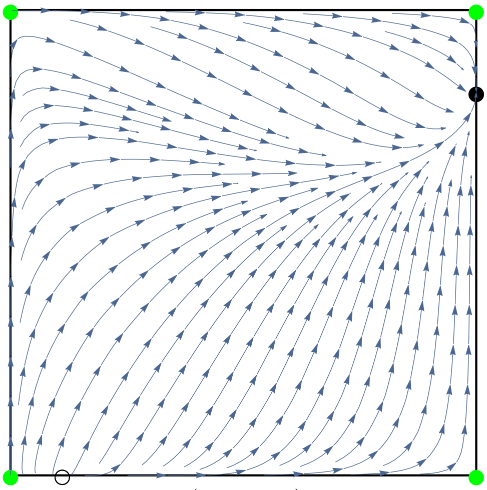

$$\begin{pmatrix} 8 & 9 & 19 \\ 7 & 29 & 22 \\ 1 & 0 & 8 \end{pmatrix}$$

$E_1^2C_1$a''

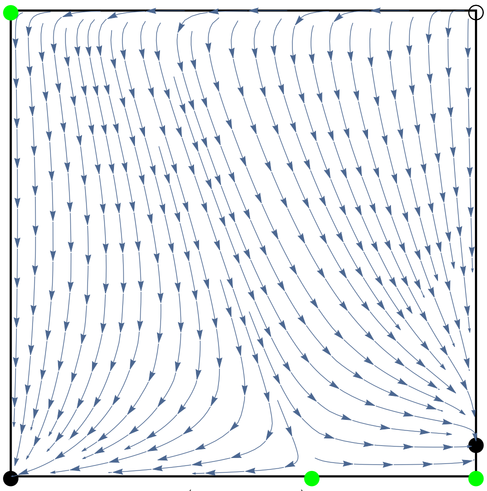

$$\begin{pmatrix} 4 & 3 & 1 \\ 5 & 14 & 27 \\ 35 & 13 & 25 \end{pmatrix}$$

$E_1^2C_1$a''

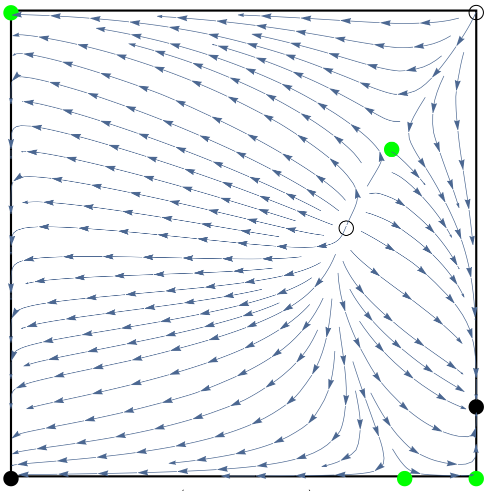

$$\begin{pmatrix} 18 & 7 & -7 \\ 19 & -26 & 4 \\ 20 & -2 & 2 \end{pmatrix}$$

$E_1^2C_1$a'''

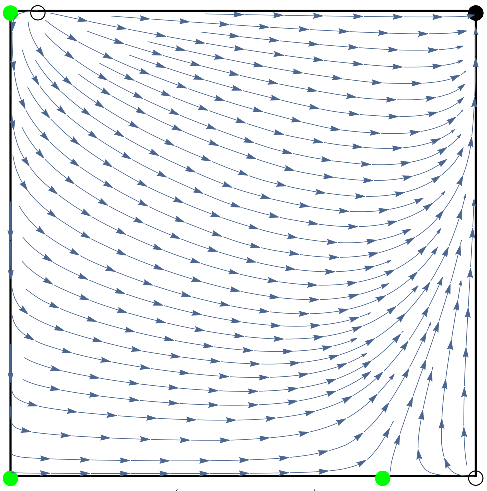

$$\begin{pmatrix} -14 & -16 & 16 \\ -13 & 2 & 12 \\ -12 & 4 & 0 \end{pmatrix}$$

Figure S2f: $\delta = 1$

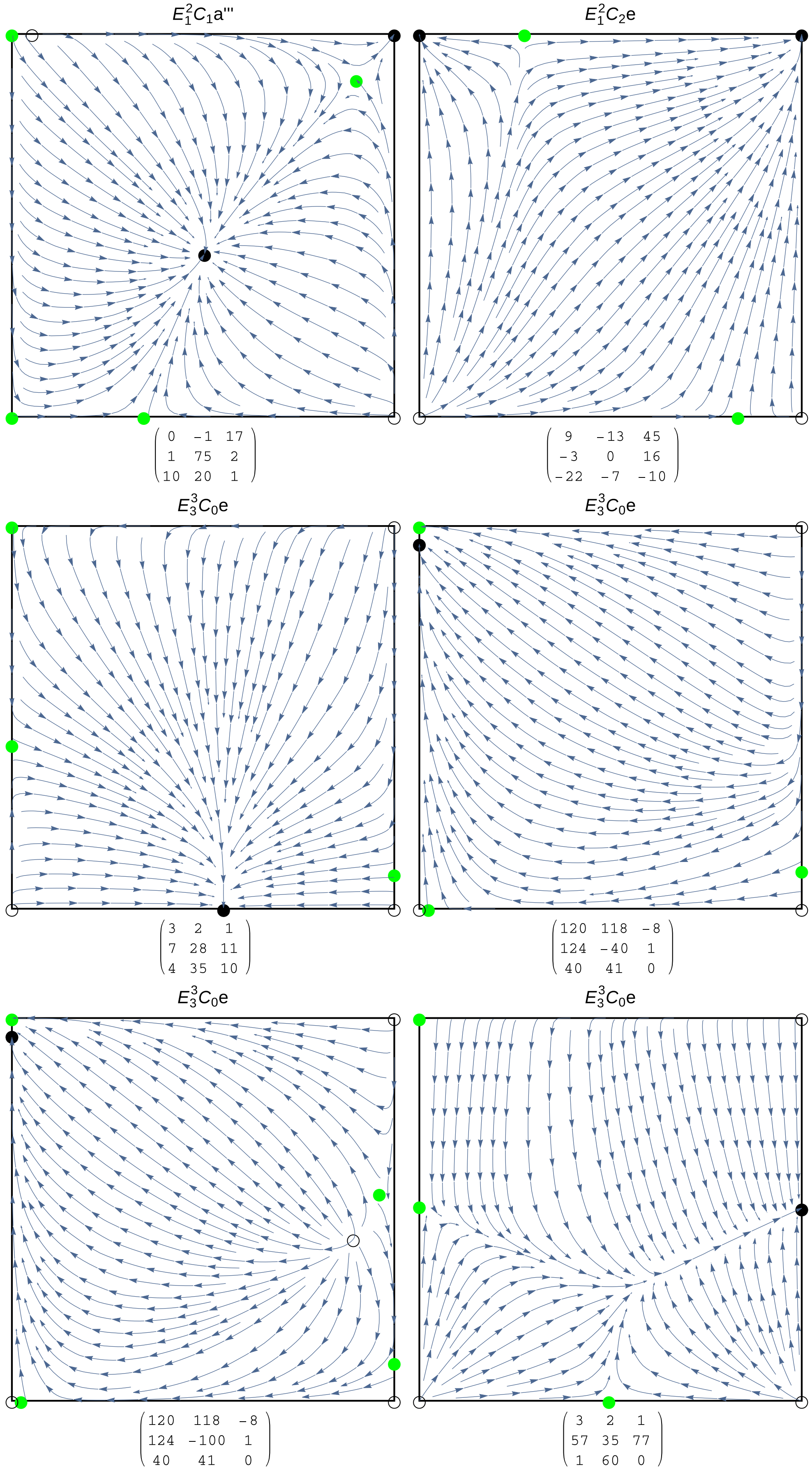

Figure S2g: $\delta = 1$

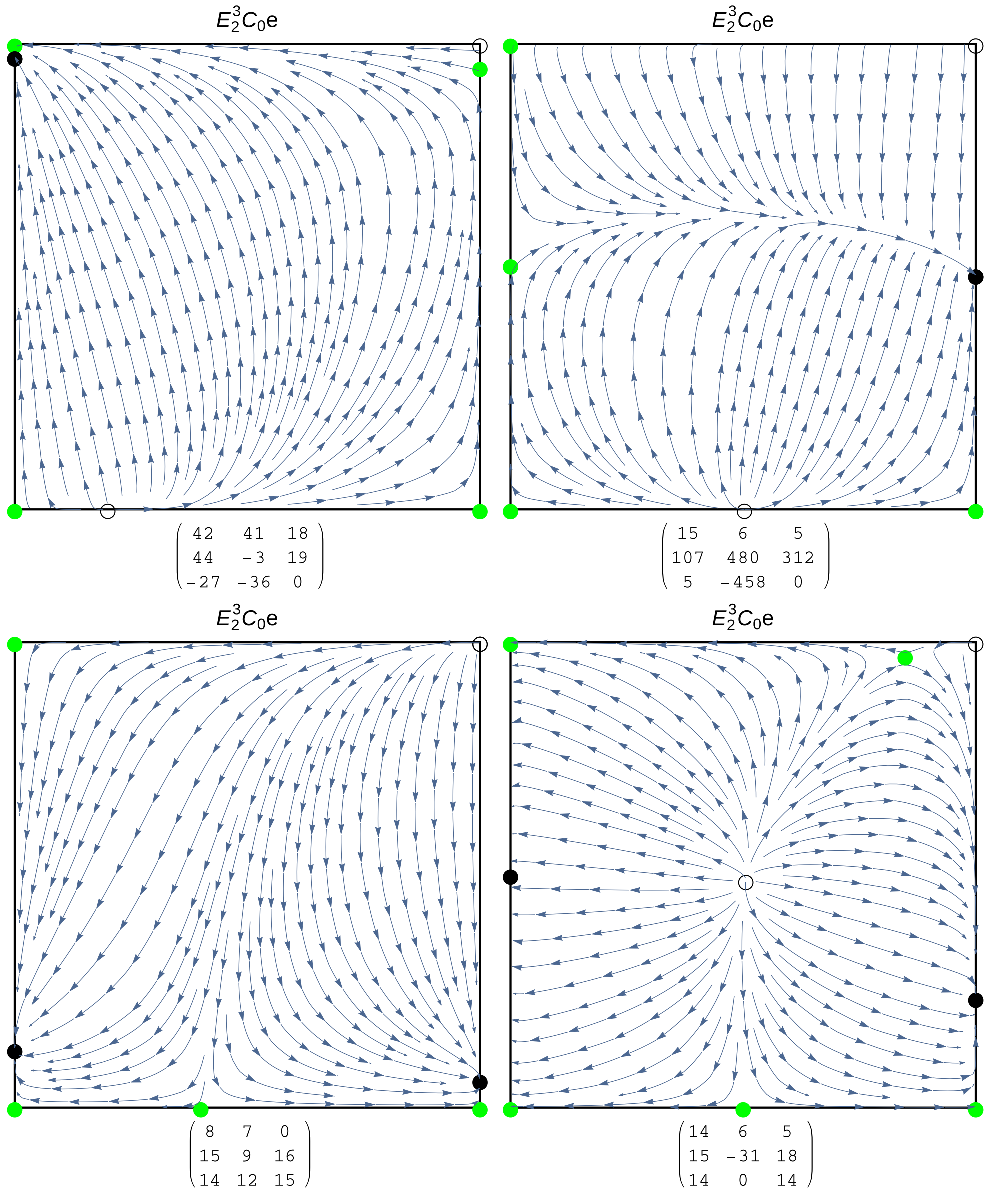

Figure S2h: $\delta = 1$

$E_2^3C_0$a

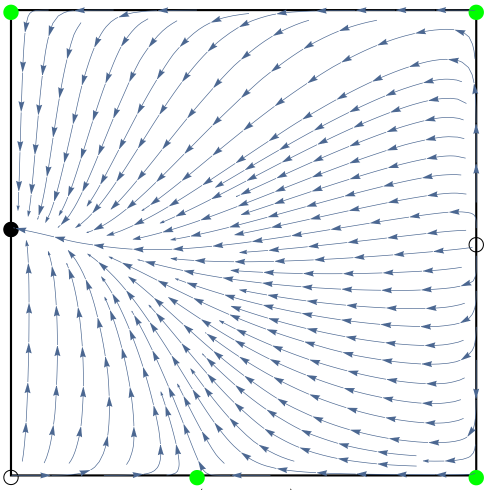

$$\begin{pmatrix} 3 & 2 & 1 \\ 10 & 9 & 0 \\ 2 & 4 & 1 \end{pmatrix}$$

$E_2^3C_0$a

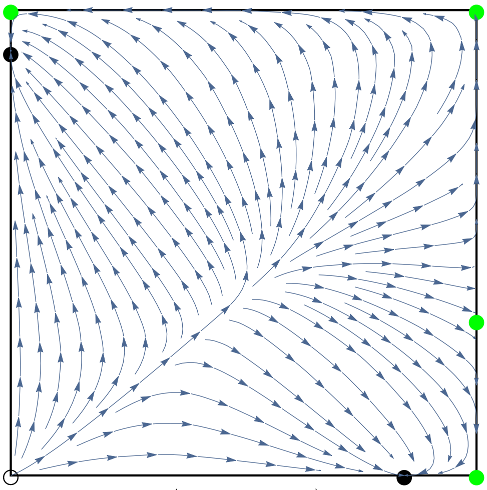

$$\begin{pmatrix} 14 & 11 & 1 \\ 20 & -60 & -1 \\ -40 & 9 & 0 \end{pmatrix}$$

$E_2^3C_0$a

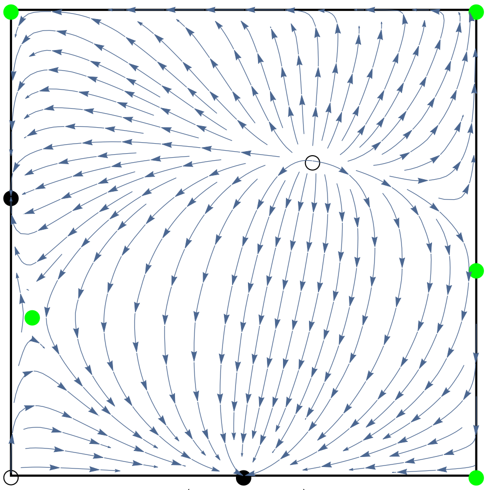

$$\begin{pmatrix} 3 & 2 & 1 \\ 9 & -34 & -4 \\ 0 & 46 & 0 \end{pmatrix}$$

$E_2^3C_0$a

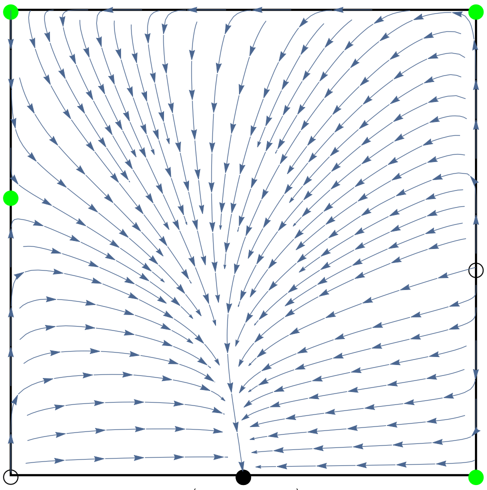

$$\begin{pmatrix} 3 & 2 & 1 \\ 9 & 43 & -4 \\ 0 & 46 & 0 \end{pmatrix}$$

$E_2^3C_1$a

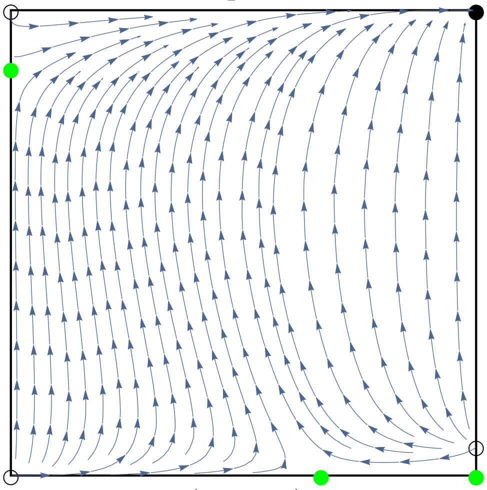

$$\begin{pmatrix} 6 & 11 & 15 \\ 7 & 2 & 0 \\ 0 & 2 & 1 \end{pmatrix}$$

Figure S2i: $\delta = 1$

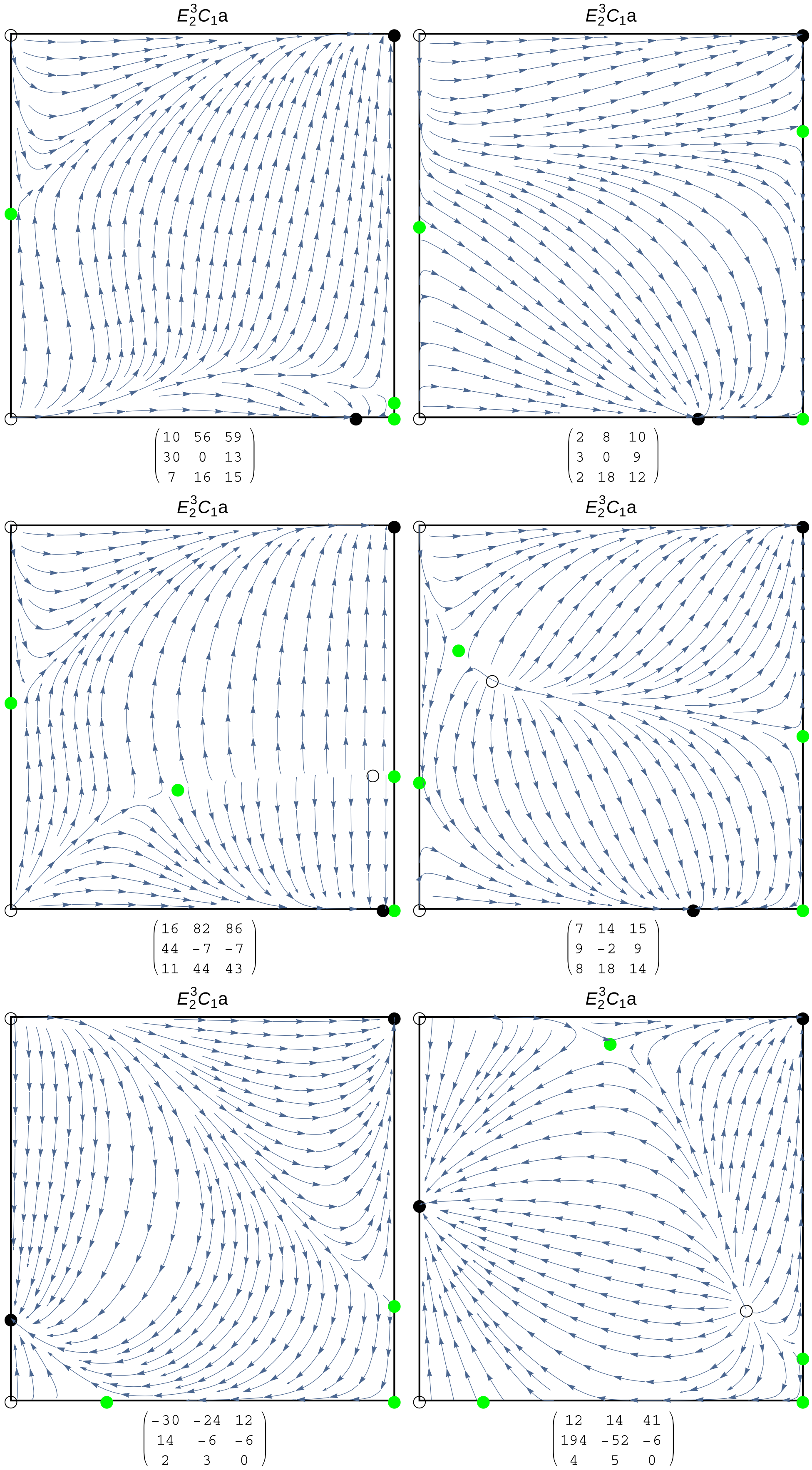

Panels 1 and 2 show different phase portraits for the same equilibrium structure, and so do panels 3 and 4.

Figure S2j: $\delta = 1$

$E_4^4C_0$b

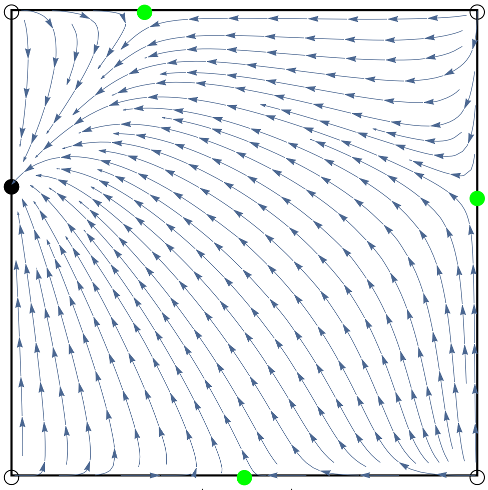

$$\begin{pmatrix} 4 & 6 & 1 \\ 10 & 2 & 3 \\ 0 & 1 & 0 \end{pmatrix}$$

$E_3^4C_0$e

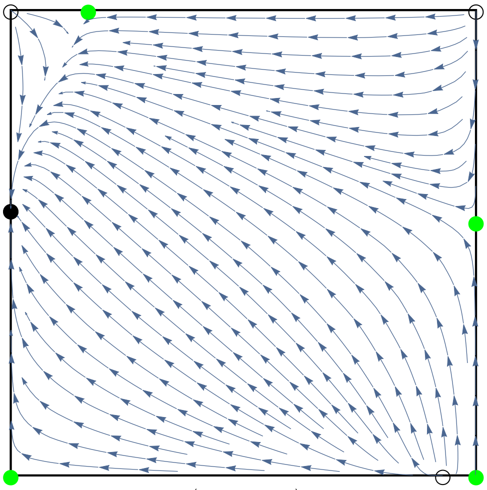

$$\begin{pmatrix} 14 & 17 & 2 \\ 17 & 12 & 7 \\ 13 & 0 & 1 \end{pmatrix}$$

$E_3^4C_0$e

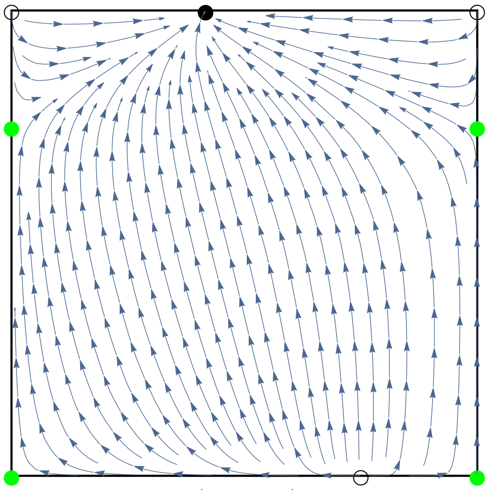

$$\begin{pmatrix} 6 & 11 & 4 \\ 8 & 5 & 6 \\ 2 & -1 & 0 \end{pmatrix}$$

$E_3^4C_0$e

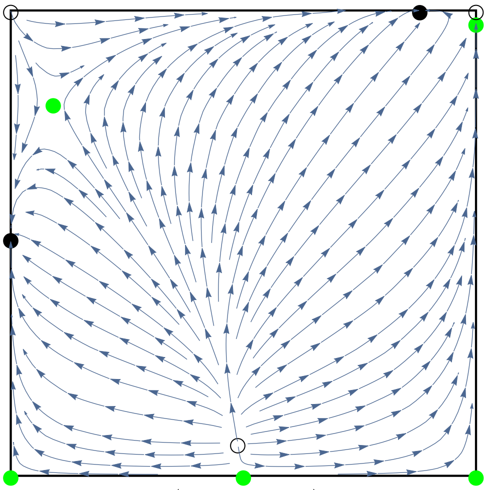

$$\begin{pmatrix} 2 & 82 & 71 \\ 52 & -120 & 73 \\ 0 & -45 & 0 \end{pmatrix}$$

$E_3^4C_0$e

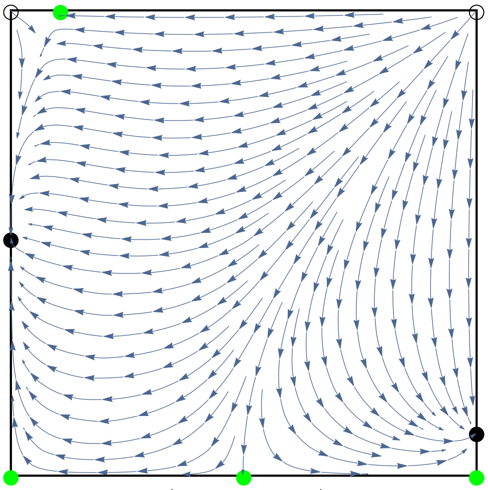

$$\begin{pmatrix} 1 & 33 & -235 \\ 24 & -70 & 27 \\ 0 & -12 & 0 \end{pmatrix}$$

Figure S2k: $\delta = 1$

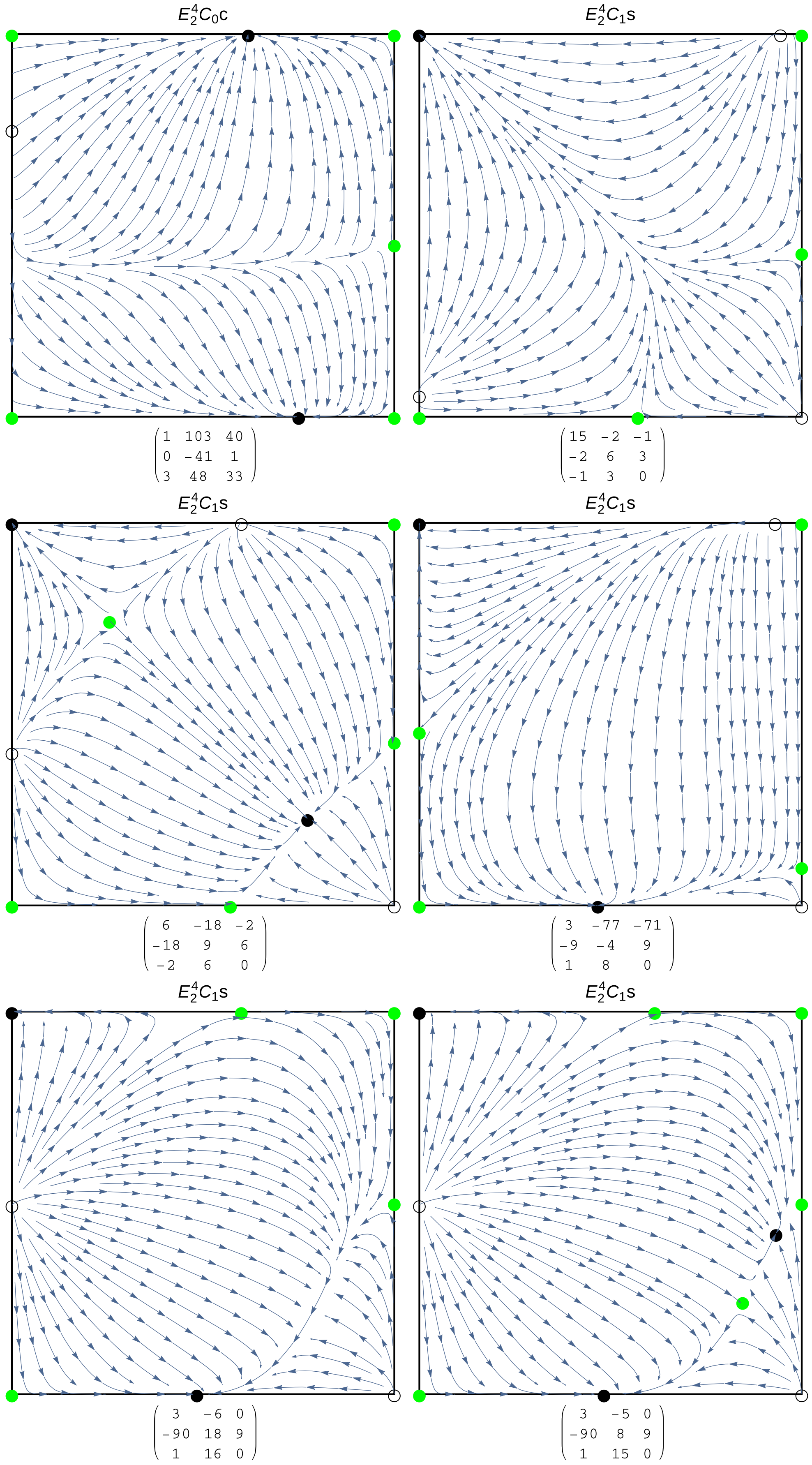

Figure S3a: $\delta = 2$

$E_0^0C_2$b

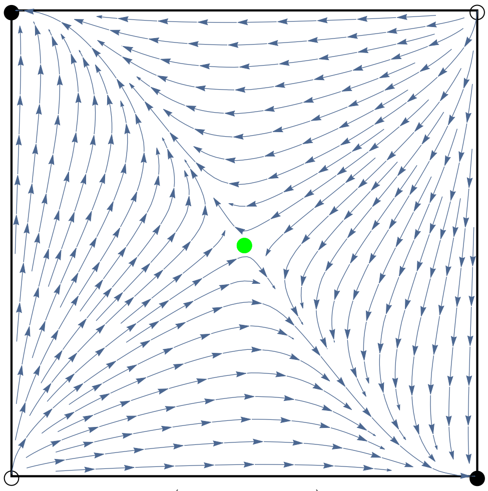

$$\begin{pmatrix} 0 & -1 & -10 \\ -5 & 0 & -5 \\ -10 & -1 & 0 \end{pmatrix}$$

$E_0^0C_2$b

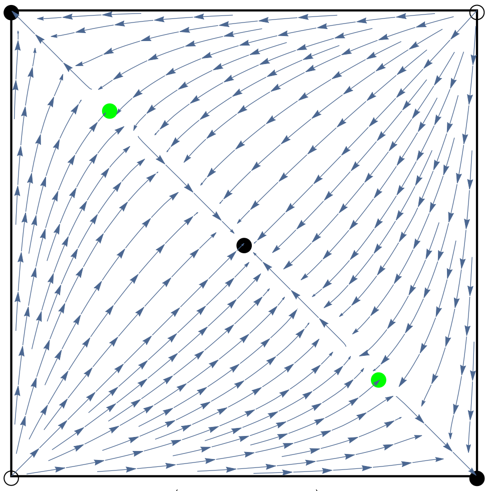

$$\begin{pmatrix} -8 & -9 & -24 \\ -9 & 1 & -9 \\ -24 & -9 & -8 \end{pmatrix}$$

$E_1^1C_1$e

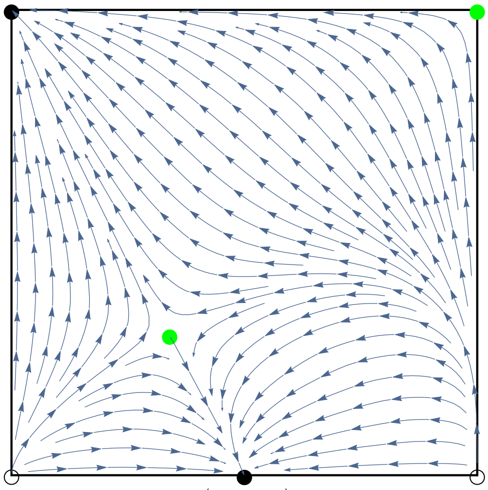

$$\begin{pmatrix} 4 & 3 & 2 \\ 3 & 0 & 1 \\ 0 & 5 & 0 \end{pmatrix}$$

$E_1^1C_1$a

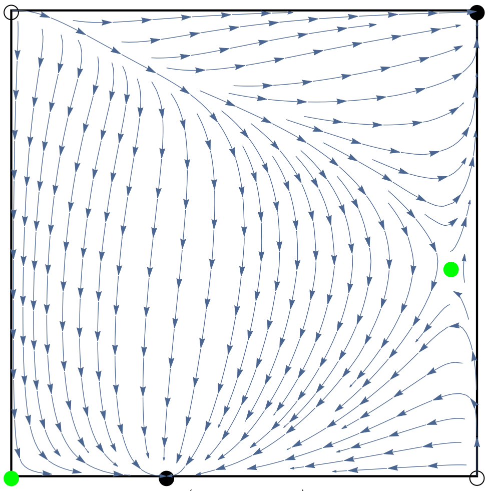

$$\begin{pmatrix} 9 & 10 & 16 \\ 13 & 0 & 15 \\ 26 & 40 & 12 \end{pmatrix}$$

$E_2^2C_0$c

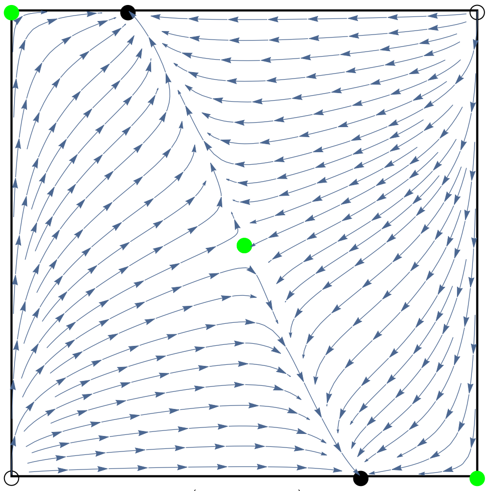

$$\begin{pmatrix} 1 & 2 & -1 \\ 0 & 2 & 0 \\ -1 & 2 & 1 \end{pmatrix}$$

$E_2^2C_0$c

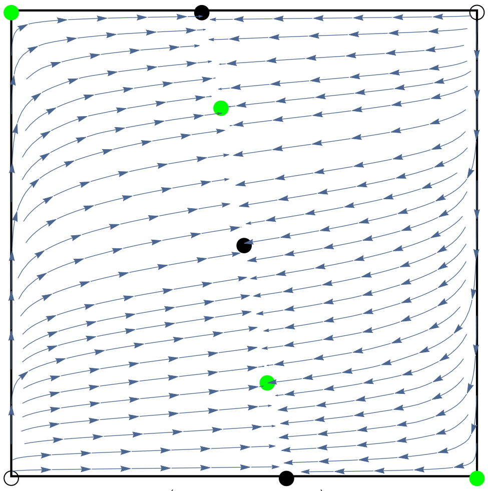

$$\begin{pmatrix} 0 & 90 & -40 \\ -30 & 107 & -30 \\ -40 & 90 & 0 \end{pmatrix}$$

Figure S3b: $\delta = 2$

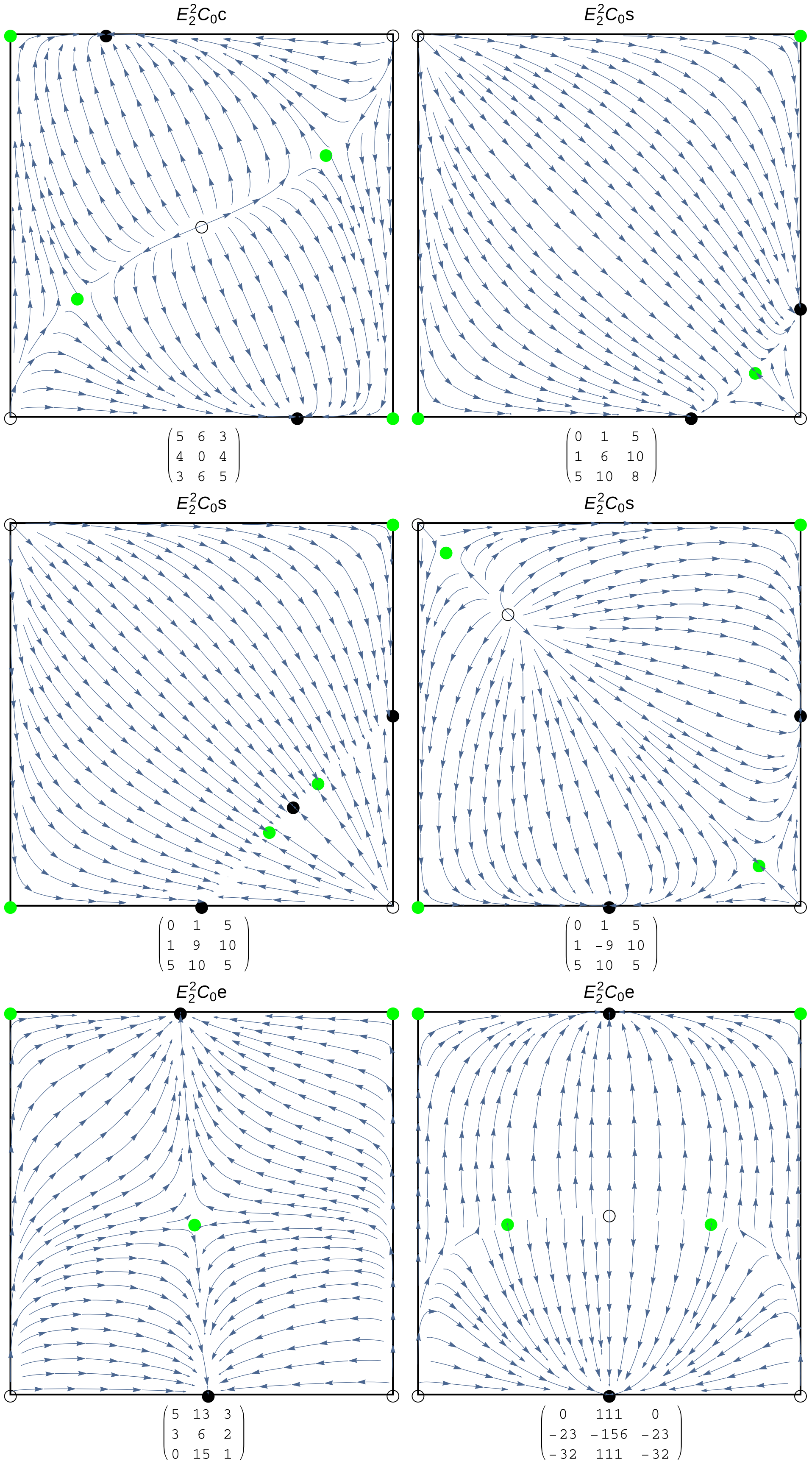

Panel 6 of Fig. S3a and this panel 1 have the same extended boundary flow and the same number of equilibria, but different stability of the central equilibrium. The same holds for panels 3 and 4, which differ only in the sign of m_{14}.

Figure S3c: $\delta = 2$

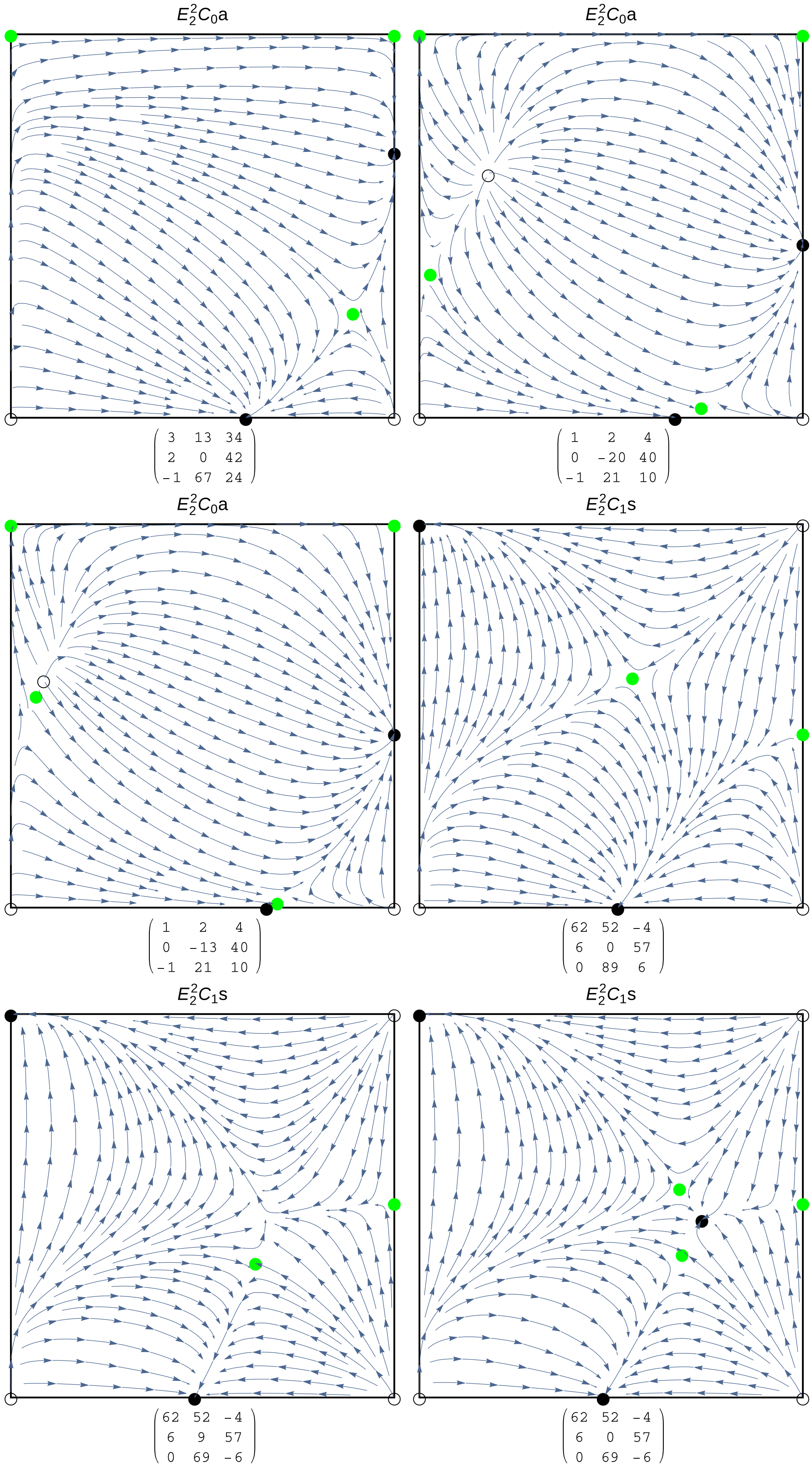

Panels 2 and 3 show different phase portraits for the same equilibrium structure, and so do panels 4 and 5.

Figure S3d: $\delta = 2$

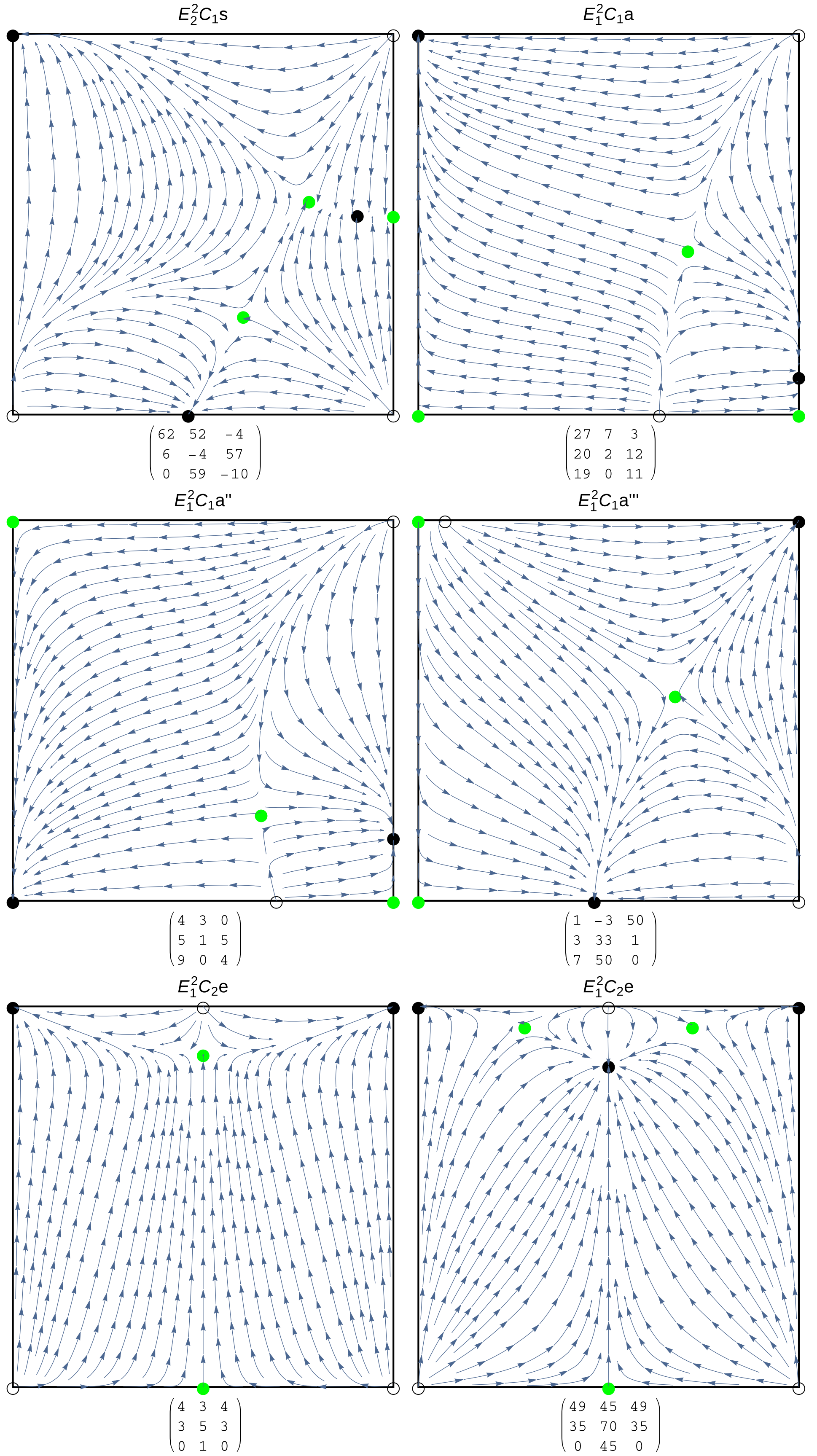

Panel 6 of Fig. S3c and this panel 1 show different phase portraits for the same equilibrium structure. There is a saddle connection in panel 5 due to the equality of the outer columns of the fitness matrix.

Figure S3e: $\delta = 2$

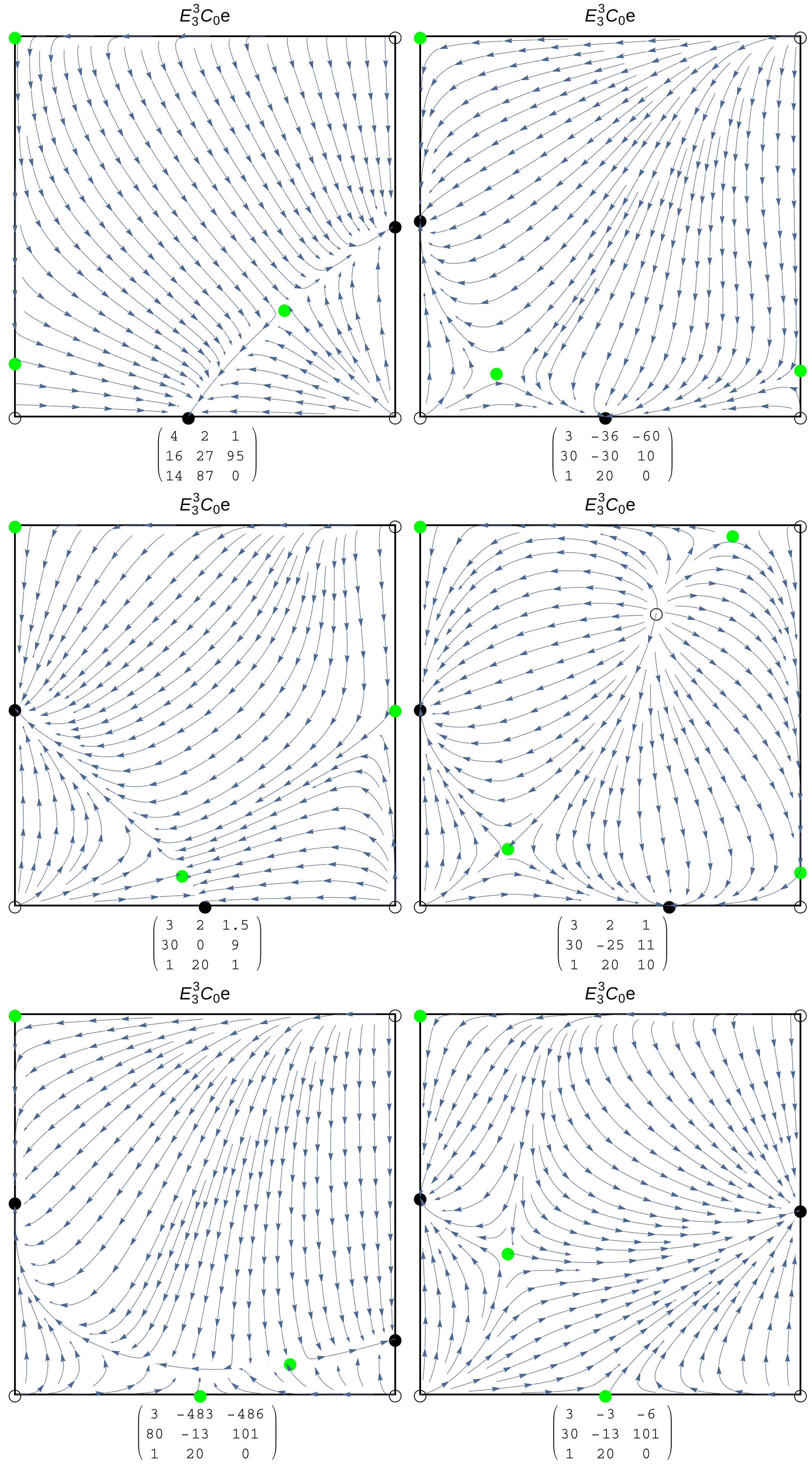

Panels 2 and 3 show different phase portraits for the same equilibrium structure, and so do panels 5 and 6.

Figure S3f: $\delta = 2$

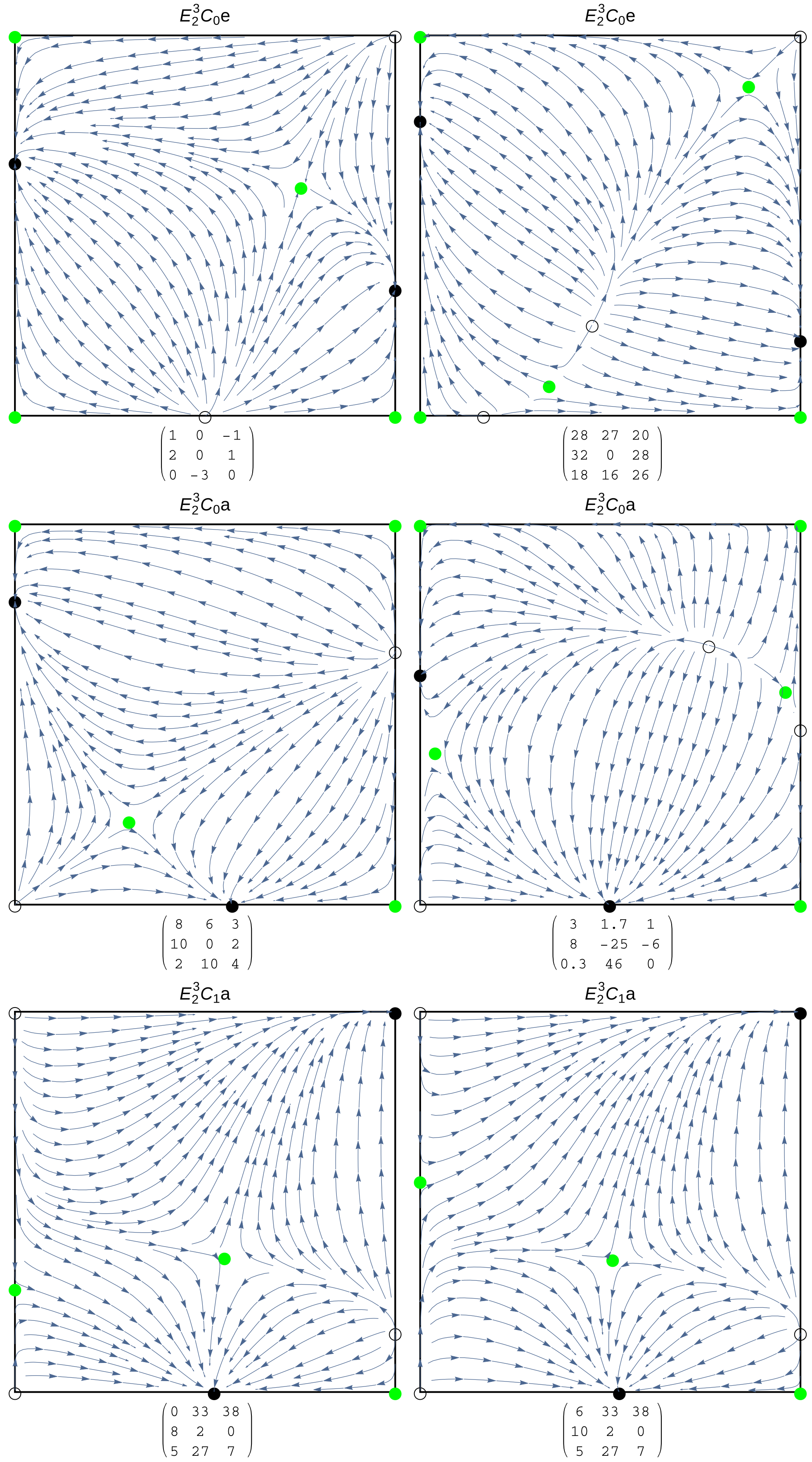

Panels 5 and 6 show different phase portraits for the same equilibrium structure.

Figure S3g: $\delta = 2$

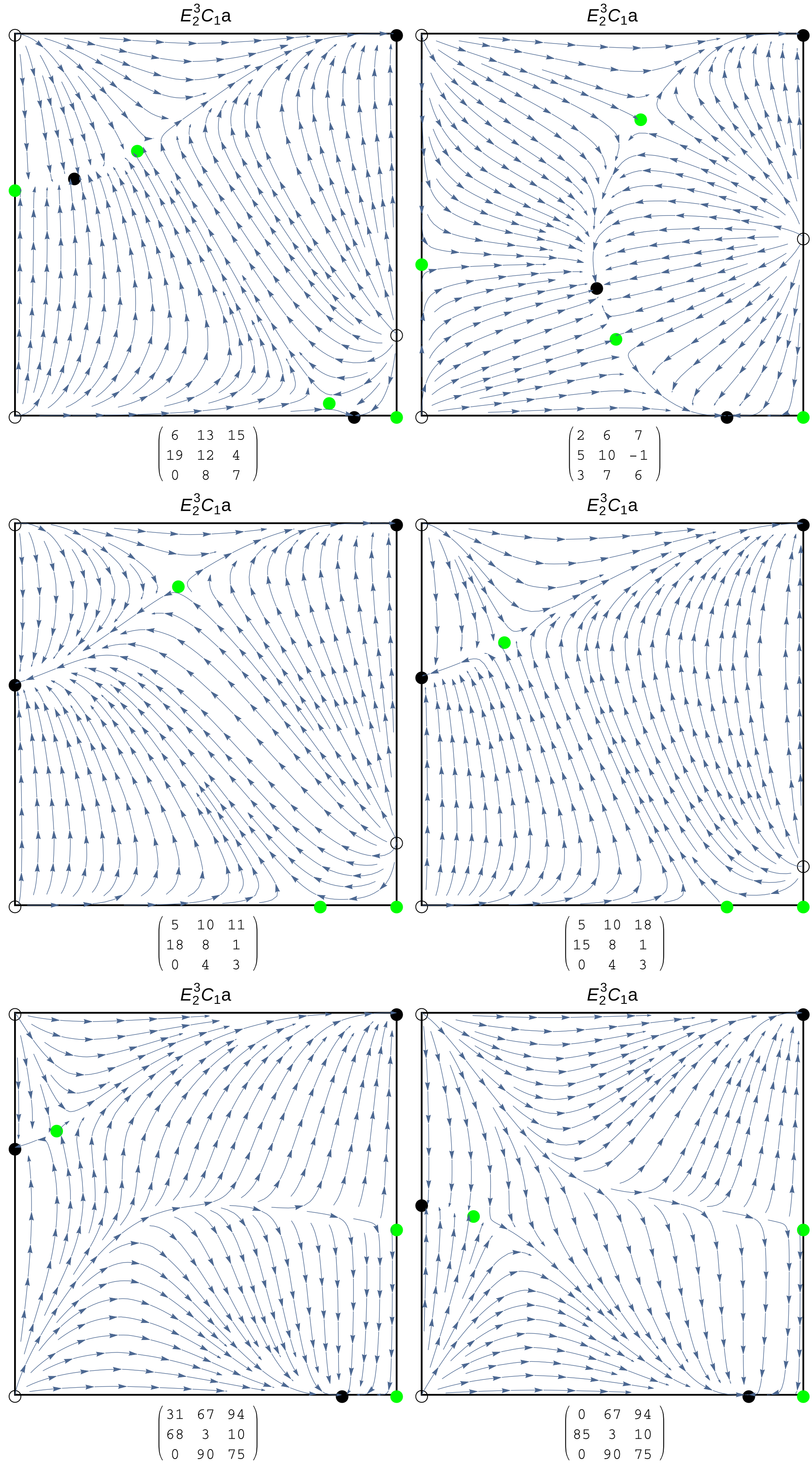

Each row of panels shows a pair with the same equilibrium structure but different phase portraits.

Figure S3h: $\delta = 2$

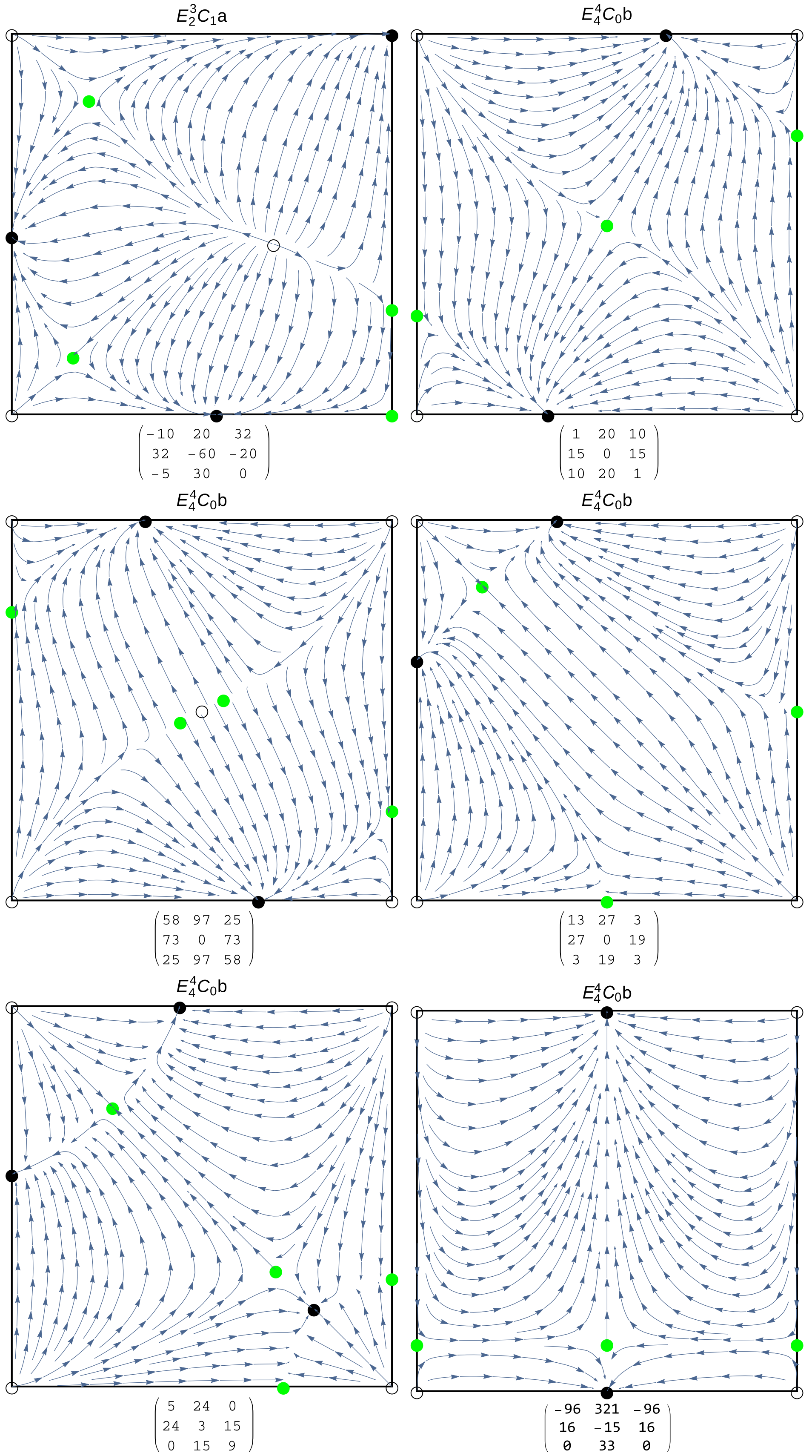

Panel 5 shows a saddle connection which is robust within the class of symmetric matrices, (8.1). Panel 6 shows two saddle connections which are robust within the class of matrices exhibiting multilinear epistasis, (7.2).

Figure S3i: $\delta = 2$

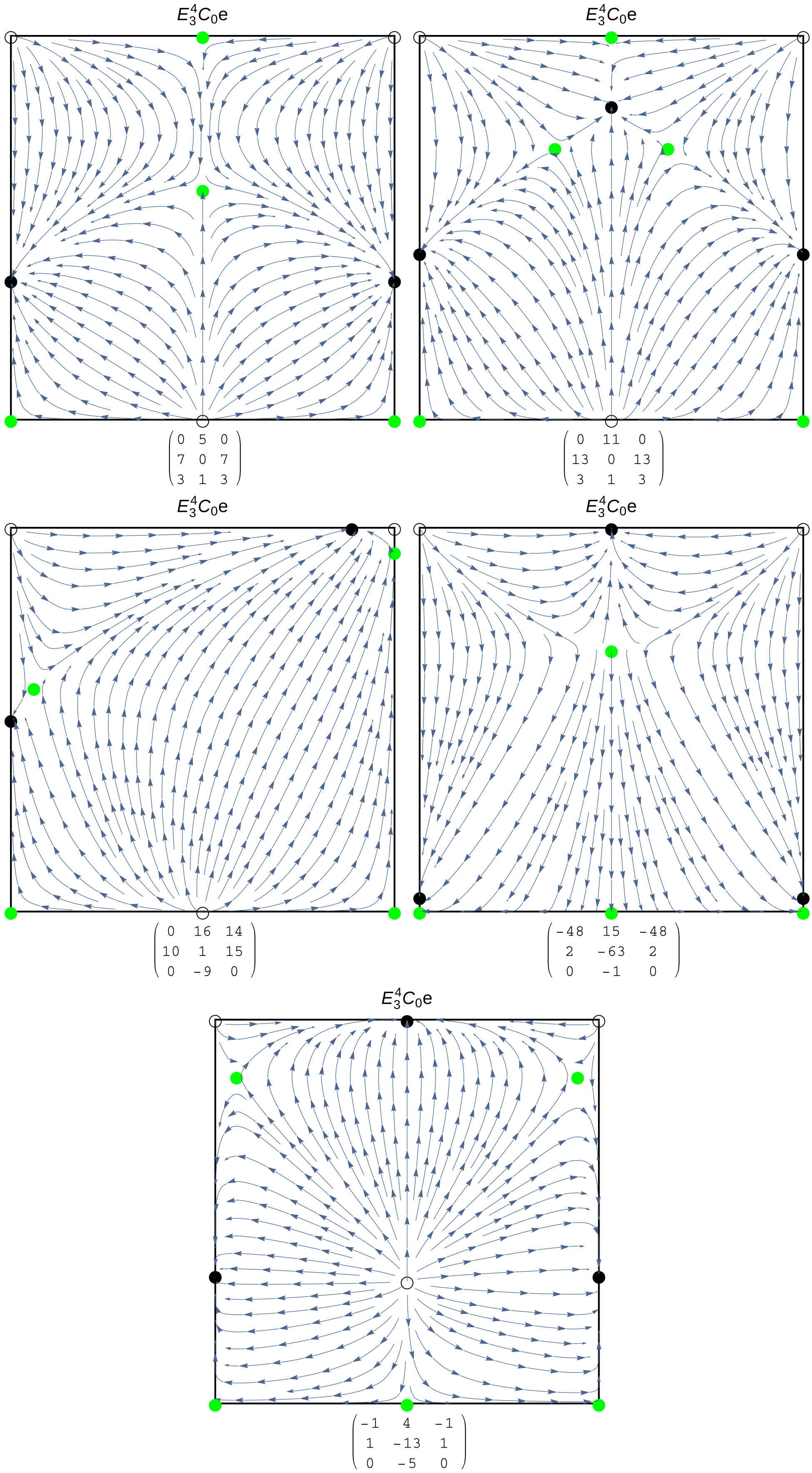

Panels 1 and 4 show a saddle connection which is due to the equality of the outer columns.

Figure S3j: $\delta = 2$

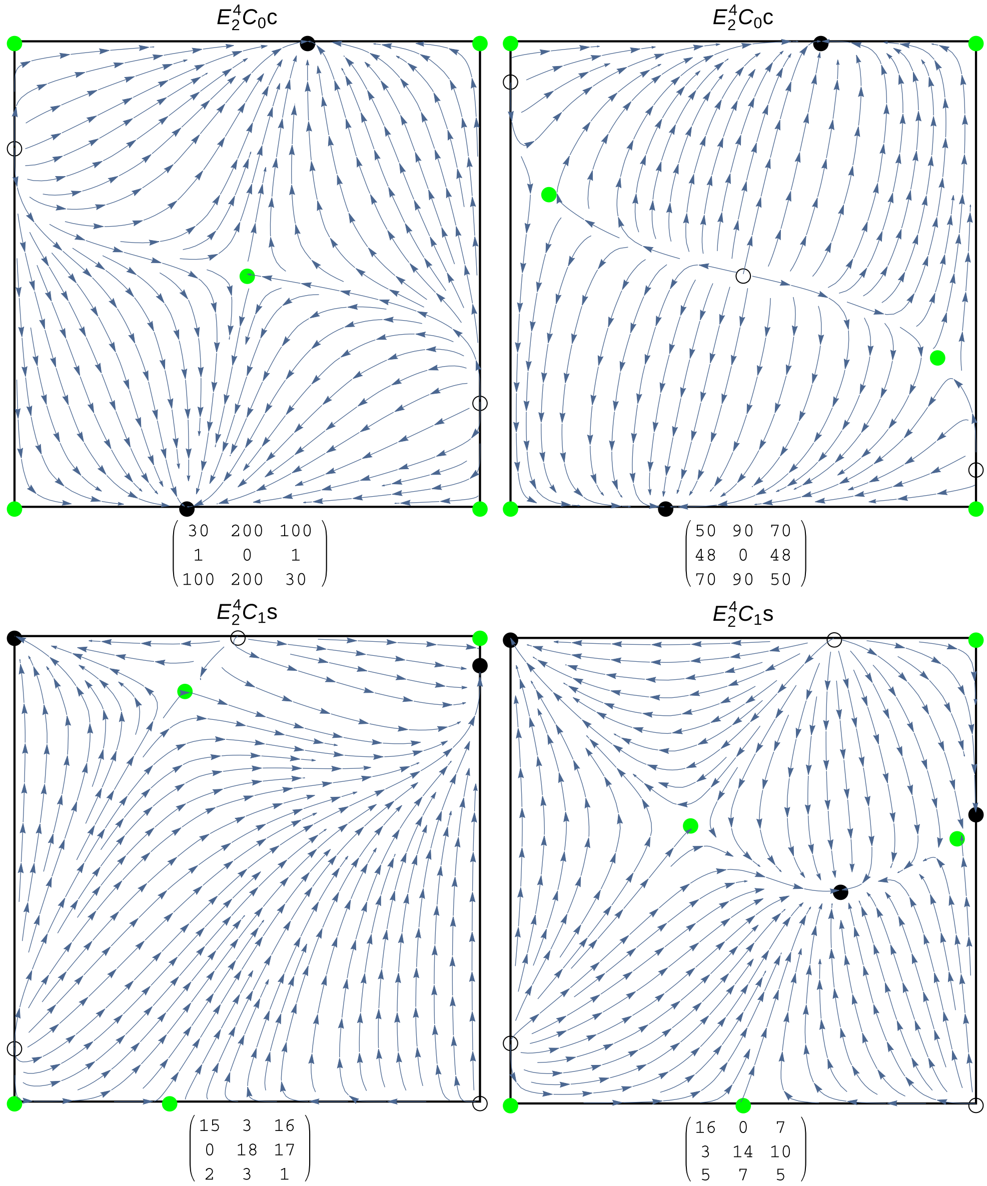

Figure S4a: $\delta = 3$

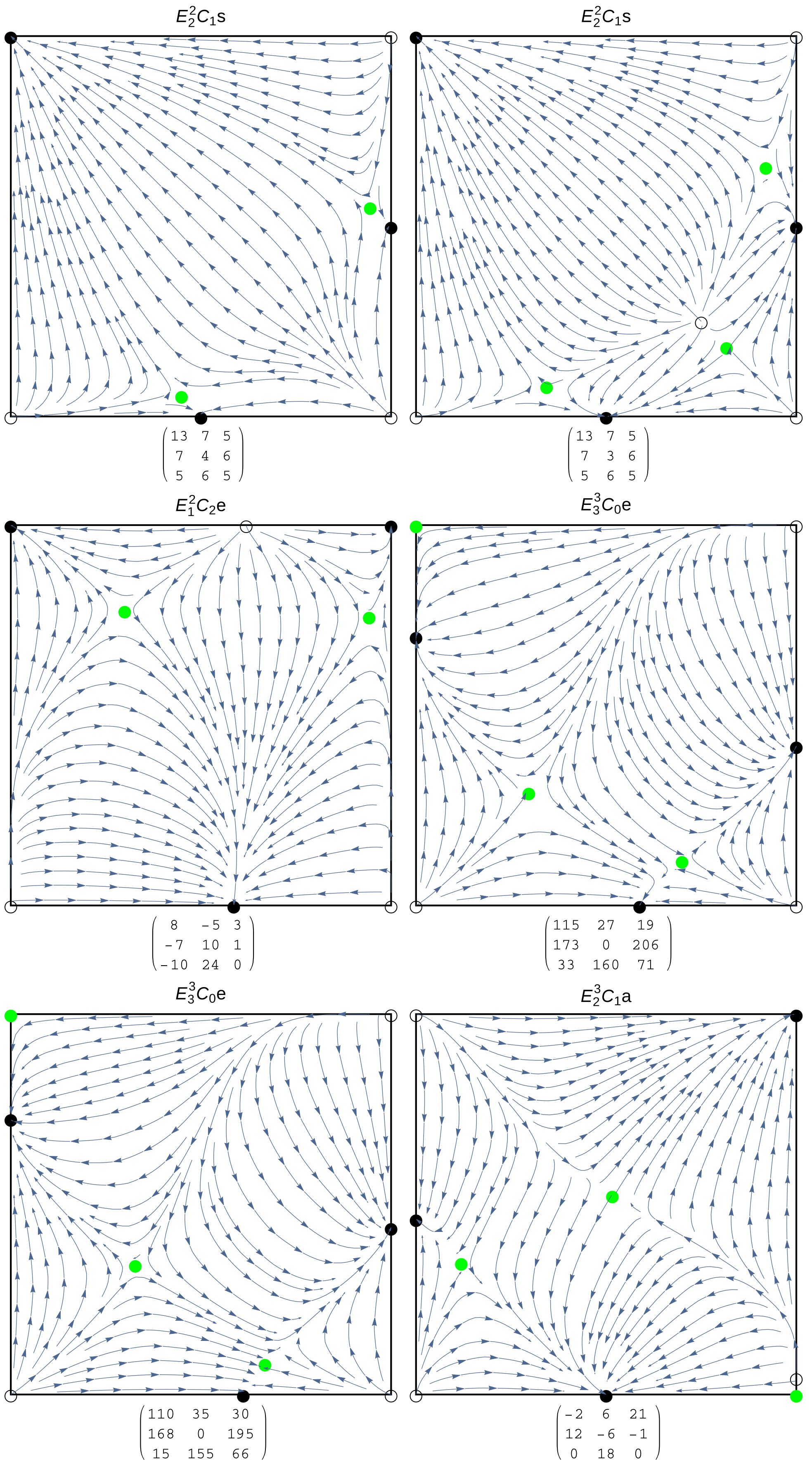

Panels 4 and 5 show different phase portraits for the same equilibrium structure.

Figure S4b: $\delta = 3$

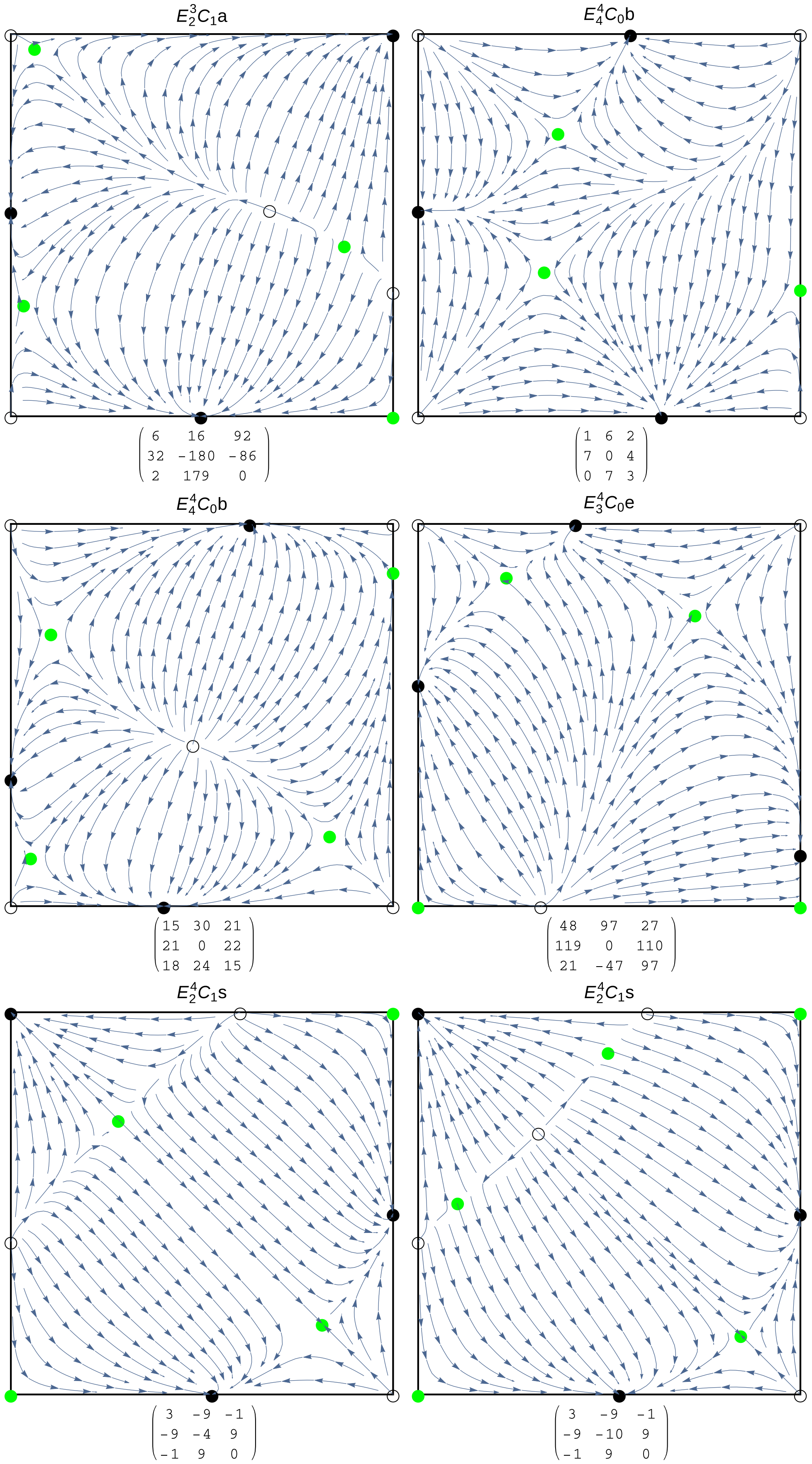

There is a saddle connection in panel 5 which is robust within the class of symmetric matrices, (8.1).

Figure S4c: $\delta = 4$

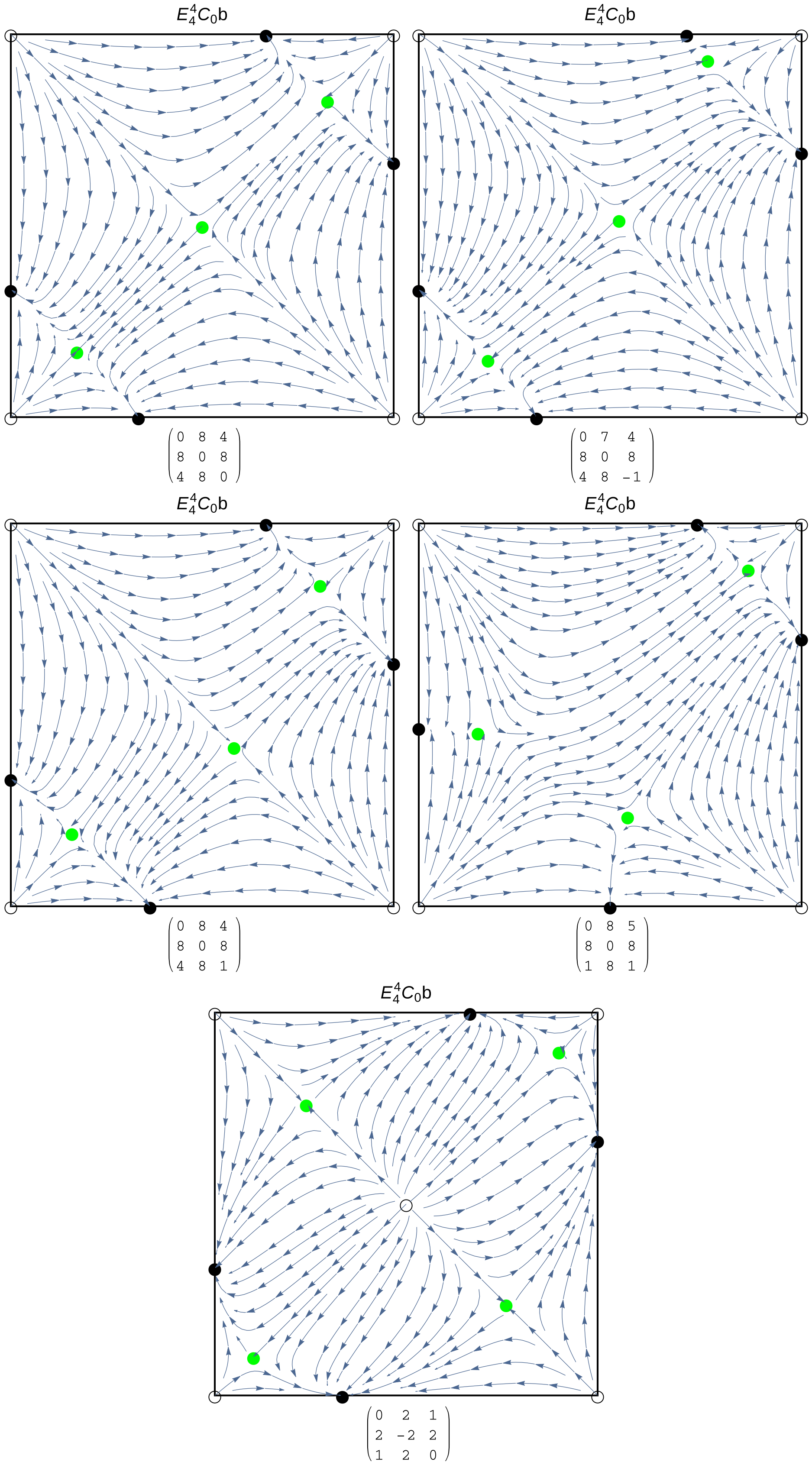

The first panel shows a double saddle connection, and the following three panels show different phase portraits that emerge from it by perturbation. The last panel shows the only phase portrait with five internal equilibria that differs from Moran's example (cf. Fig. S1d, panel 6). It is generated by a matrix of symmetry class b.

S2 Supplementary Information: Tables

Table S1: List of all potential extended boundary flows

$E^0_0C_1s$:	1
$E^0_0C_1a$:	1
$E^0_0C_2b$:	2
$E^1_1C_0e$, $E^1_0C_1e$:	0, 1
$E^1_1C_0a$, $E^1_0C_1a$:	0, 1
$E^1_1C_1e$, $E^1_0C_2e$:	1, 2
$E^1_1C_1a$, $E^1_0C_2a$:	1, 2
$E^2_2C_0c$, $E^2_0C_2c$:	0, 1, 2
$E^2_2C_0s$, $E^2_0C_2s$:	0, 1, 2
$E^2_2C_0e$, $E^2_0C_2e$:	0, 1(2), 2
$E^2_2C_0a$, $E^2_0C_2a$:	0, 1(2), 2
$E^2_2C_1s$, $E^2_0C_3s$:	1, 2, 3
$E^2_1C_1a$, $E^2_1C_1a'$:	0, 1(2), 2
$E^2_1C_0e$:	-1*, $0(2^{\dagger})$, 1
$E^2_1C_0a$:	-1*, $0(2^{\dagger})$, 1
$E^2_1C_1a''$:	0, $1(2^{\dagger})$, 2
$E^2_1C_1a'''$:	0, $1(2^{\dagger})$, 2
$E^2_1C_2e$:	1, $2(2^{\dagger})$, 3
$E^3_3C_0e$, $E^3_0C_3e$:	0, 1(3), 2(3), 3
$E^3_2C_0e$, $E^3_1C_1e$:	-1*, 0(1*+2), 1(3), 2
$E^3_2C_0a$, $E^3_1C_1a$:	-1°, 0(3), 1(3), 2
$E^3_2C_1a$, $E^3_1C_2a$:	0, 1(3), 2(3), 3
$E^4_4C_0b$, $E^4_0C_4b$:	0, 1, 2(2), 3, 4
$E^4_3C_0e$, $E^4_1C_2e$:	-1°, 0(3), 1(4), 2(3), 3
$E^4_2C_0c$:	-2*, $-1(2^{\dagger,*})$, $0(1^*+2^{\dagger})$, $1(2^{\dagger})$, 2
$E^4_2C_1s$:	-1°, $0(2^{\dagger})$, $1(2+2^{\dagger})$, $2(2^{\dagger})$, 3

For each boundary-flow class the potential values of δ are given. The number in parentheses gives the number of different extended boundary flows resulting in the same δ. For the flow-reversal pairs, the given numbers apply to each member of the pair. An * indicates an extended boundary flow whose existence was disproved. A ° indicates an extended boundary flow whose non-existence is conjectured. A † indicates a pair of extended boundary flows, where the number of saturated edge equilibria is different but, up to symmetry operations, they are in fact flow reversals of each other (cf. Fig. 3).

Table S2: List of all extended boundary flows and their occurrence in the special cases

$\mathsf{E}_0^0\mathsf{C}_1\mathsf{s}$:	$1^{a,h,s}$
$\mathsf{E}_0^0\mathsf{C}_1\mathsf{a}$:	1^h
$\mathsf{E}_0^0\mathsf{C}_2\mathsf{b}$:	$2^{h,b}$
$\mathsf{E}_1^1\mathsf{C}_0\mathsf{e}$, $\mathsf{E}_0^1\mathsf{C}_1\mathsf{e}$:	0, 1^l
$\mathsf{E}_1^1\mathsf{C}_0\mathsf{a}$, $\mathsf{E}_0^1\mathsf{C}_1\mathsf{a}$:	0, 1
$\mathsf{E}_1^1\mathsf{C}_1\mathsf{e}$, $\mathsf{E}_0^1\mathsf{C}_2\mathsf{e}$:	1^l, 2
$\mathsf{E}_1^1\mathsf{C}_1\mathsf{a}$, $\mathsf{E}_0^1\mathsf{C}_2\mathsf{a}$:	1^l, 2^l
$\mathsf{E}_2^2\mathsf{C}_0\mathsf{c}$, $\mathsf{E}_0^2\mathsf{C}_2\mathsf{c}$:	$0^{l,c}$, $1^{l,m}$, $2^{l,c}$
$\mathsf{E}_2^2\mathsf{C}_0\mathsf{s}$, $\mathsf{E}_0^2\mathsf{C}_2\mathsf{s}$:	$0^{l,s}$, 1^l, $2^{l,s}$
$\mathsf{E}_2^2\mathsf{C}_0\mathsf{e}$, $\mathsf{E}_0^2\mathsf{C}_2\mathsf{e}$:	0, $1(1^m+1^a)$, 2
$\mathsf{E}_2^2\mathsf{C}_0\mathsf{a}$, $\mathsf{E}_0^2\mathsf{C}_2\mathsf{a}$:	0, 1(2), 2
$\mathsf{E}_2^2\mathsf{C}_1\mathsf{s}$, $\mathsf{E}_0^2\mathsf{C}_3\mathsf{s}$:	$1^{l,s}$, 2, 3^s
$\mathsf{E}_1^2\mathsf{C}_1\mathsf{a}$, $\mathsf{E}_1^2\mathsf{C}_1\mathsf{a}'$:	0, $1(1+1^l)$, 2^l
$\mathsf{E}_1^2\mathsf{C}_0\mathsf{e}$:	0, 1^m
$\mathsf{E}_1^2\mathsf{C}_0\mathsf{a}$:	0, 1
$\mathsf{E}_1^2\mathsf{C}_1\mathsf{a}''$:	0, 1, 2
$\mathsf{E}_1^2\mathsf{C}_1\mathsf{a}'''$:	0, 1, 2^m
$\mathsf{E}_1^2\mathsf{C}_2\mathsf{e}$:	1^m, 2, 3^m
$\mathsf{E}_3^3\mathsf{C}_0\mathsf{e}$, $\mathsf{E}_0^3\mathsf{C}_3\mathsf{e}$:	0^l, $1(2+1^l)$, 2(3), 3
$\mathsf{E}_2^3\mathsf{C}_0\mathsf{e}$, $\mathsf{E}_1^3\mathsf{C}_1\mathsf{e}$:	0(2), $1(2+1^l)$, 2^l
$\mathsf{E}_2^3\mathsf{C}_0\mathsf{a}$, $\mathsf{E}_1^3\mathsf{C}_1\mathsf{a}$:	0(3), 1(3), 2
$\mathsf{E}_2^3\mathsf{C}_1\mathsf{a}$, $\mathsf{E}_1^3\mathsf{C}_2\mathsf{a}$:	0, 1(3), 2(3), 3
$\mathsf{E}_4^4\mathsf{C}_0\mathsf{b}$, $\mathsf{E}_0^4\mathsf{C}_4\mathsf{b}$:	$0^{ou,a,b}$, 1, $2(1^s+1^{m,c})$, 3, $4^{m,b}$
$\mathsf{E}_3^4\mathsf{C}_0\mathsf{e}$, $\mathsf{E}_1^4\mathsf{C}_2\mathsf{e}$:	$0(1+2^m)$, 1(4), $2(1+2^m)$, 3
$\mathsf{E}_2^4\mathsf{C}_0\mathsf{c}$:	$0^{m,c}$, 1, $2^{ou,a,c}$
$\mathsf{E}_2^4\mathsf{C}_1\mathsf{s}$:	0, $1(2+1^{m,s})$, 2, 3^s

For each boundary-flow class the values of δ are given. The number in parentheses gives the number of different extended boundary flows resulting in the same δ. The superscripts stand for assumptions on the fitness parameters, under which the indicated extended boundary flow can occur: ou, l, a, h, m, s, c, and b indicate marginal over- or underdominance (Sect. 5), linear isoclines (Sect. 6), additive fitnesses (Sect. 6.1), the haploid model (Sect. 6.2), the multilinear epistasis model (Sect. 7), the symmetric model (Sect. 8), the centrosymmetric model (Sect. 9), and bisymmetric fitness matrices, respectively. A superscript a or h implies that this extended boundary flow can be generated by both the model with linear isoclines and that with multilinear epistasis. A superscript b implies that this extended boundary flow can be generated by both the symmetric and centrosymmetric model. Therefore, in the first case (a or h) the superscripts l and m are omitted; in the second case (b), s and c are omitted.

Table S3: List of parameter combinations for Theorem 7.1

Boundary flow	$\delta = 0$	$\delta = 1$	$\delta = 2$	$\delta = 3$	$\delta = 4$
$\mathsf{E}_0^0\mathsf{C}_1\mathsf{s}$	–	$(1, 2, 0, 0, -\frac{2}{5})$	–	–	–
$\mathsf{E}_0^0\mathsf{C}_1\mathsf{a}$	–	$(3, 2, 0, -1, \frac{5}{8})$	–	–	–
$\mathsf{E}_0^0\mathsf{C}_2\mathsf{b}$	–	–	$(1, 1, 0, 0, -1)^\dagger$	–	–
$\mathsf{E}_2^2\mathsf{C}_0\mathsf{c}$	–	$(2, 1, 4, 0, -2)$	–	–	–
$\mathsf{E}_2^2\mathsf{C}_0\mathsf{e}$	–	$(4, 3, -2, 4, 0)$,<br>$(1, 1, \frac{1}{4}, -\frac{9}{5})$	–	–	–
$\mathsf{E}_1^2\mathsf{C}_0\mathsf{e}$	–	$(3, 4, -4, 2, \frac{7}{8})$	–	–	–
$\mathsf{E}_1^2\mathsf{C}_1\mathsf{a}'''$	–	–	$(3, 2, 4, -1, -2)$	–	–
$\mathsf{E}_1^2\mathsf{C}_2\mathsf{e}$	–	$(3, 6, 4, -5, -1)$	–	$(6, 9, 25, -\frac{17}{2}, -1)$	–
$\mathsf{E}_4^4\mathsf{C}_0\mathsf{b}$	$(0, 0, 1, 1, 0)^\dagger$	–	$(0, 0, 1, 5, -6)^\dagger$	–	$(0, 0, 1, 1, -3)^\dagger$
$\mathsf{E}_3^4\mathsf{C}_0\mathsf{e}$	$(2, 1, 4, -4, 8)$	–	$(1, 2, 3, 5, -10)$ or<br>$(1, 2, 3, 5, -11)$	–	–
$\mathsf{E}_2^4\mathsf{C}_0\mathsf{c}$	$(0, 0, 1, -5, 11)^{*,\dagger}$	–	$(0, 0, 1, -1, 0)^\dagger$	–	–
$\mathsf{E}_2^4\mathsf{C}_1\mathsf{s}$	–	$(1, 2, -2, 4, 4)^*$	–	–	–

Parameter combinations yielding the equilibrium structures stated in Theorem 7.1. The table has the same structure as Table 1, except that only one of a pair of flow-reversal cases is listed. The numbers in parentheses are $(a_1, a_2, d_1, d_2, e_{22})$. The other parameters can be inferred from (7.2). An asterisk, *, indicates that by reversing the signs of all parameters, the second possible equilibrium structure listed in Table 1 is obtained, i.e., the one with a source instead of a sink. A dagger, †, indicates that this parameter combination is consistent with the symmetric viability model, (9.1).
